# HIV-1 Gag, Pol, and Env diversified with limited adaptation since the 1980s

**DOI:** 10.1128/mbio.01749-23

**Published:** 2024-02-08

**Authors:** Eric Lewitus, Yifan Li, Hongjun Bai, Phuc Pham, Morgane Rolland

**Affiliations:** 1 U.S. Military HIV Research Program, Walter Reed Army Institute of Research, Silver Spring, Maryland, USA; 2 Henry M. Jackson Foundation for the Advancement of Military Medicine Inc., Bethesda, Maryland, USA; University of North Carolina at Chapel Hill, Chapel Hill, North Carolina, USA; University of California, San Diego, San Diego, California, USA

**Keywords:** HIV-1, evolution, diversity

## Abstract

Knowledge of HIV-1 global sequence diversity is critical for developing an effective prophylactic against HIV-1 infection. We developed the Hervé platform to analyze and visualize trends in HIV-1 diversification. Using Hervé, we analyzed 4,830 Env, 4,407 Gag, and 3,002 Pol publicly available independent sequences corresponding to subtypes A1, A6, B, C, D, F1, and G and circulating recombinant forms (CRFs) 01_AE, 02_AG, and 07_BC; sequences were sampled between 1980 and 2020 from 82 countries. HIV-1 diversified with a median of 1.82 amino acid substitutions per year in Env, 0.297 in Gag, and 0.779 in Pol. Yet, Env subtype B diversification plateaued post-2000. Pairwise diversity within subtypes and CRFs increased by 41.82% (range = 24.85%–54.41%) in Env, 56.93% (15.38%–89.16%) in Gag, and 46.12% (11.70%–70.57%) in Pol. Consensus sequences based on sequences sampled in each decade remained relatively stable over time. Similarly, at antibody epitope sites, only 0–8 residues that were minority variants became consensus over time in any subtype/CRF and only one known drug resistance mutation site differed from the reference (subtype G). The apparent contradiction between the fast diversification of HIV-1 and its limited adaptation illustrates that HIV-1 evolution is not directional and its consensus is at the intersection of millions of within-host selective processes occurring in a star-like manner. While a consensus sequence is a better representation of HIV-1 diversity than any individual sequence, consensus sequences have progressively become more distant from the circulating sequences they represent.

**IMPORTANCE:**

Global surveillance of HIV-1 sequences is critical for designing relevant prophylactic and therapeutic interventions to infection. We designed an open-source platform, Hervé, for analyzing and visualizing the diversification dynamics of HIV-1 protein sequences. We characterized the evolution of over 12,000 HIV-1 Env, Gag, and Pol protein sequences from 1980–2020 and found that, despite a steady increase in intra-subtype and circulating recombinant form diversity, the most frequent residue at each site, i.e., the consensus, has varied only moderately.

## INTRODUCTION

In 2022, 39 million people were living with HIV-1 and 630,000 died of AIDS-related illnesses (1). While nearly three-quarters of people living with HIV-1 (PLWH) are estimated to have access to antiretroviral therapy, an HIV-1 vaccine remains the best strategy for limiting new infections. Surveillance of HIV-1 diversity is integral to the development of a vaccine, as vaccine efficacy can be modified by specific mutations (2). It is as important for therapies as certain mutations can abrogate the effect of antiretrovirals (3).

Since its transmission to humans over a century ago in central Africa (4, 5), HIV-1’s main group (group M) has diversified into 10 subtypes (6) and 132 circulating and numerous unique recombinant forms (CRFs/URFs) (https://www.hiv.lanl.gov/components/sequence/HIV/crfdb/crfs.comp), with some lineages spreading globally. The 10 subtypes of group M (A, B, C, D, F, G, H, J, K, and L) account for 75% of infections, and recombinant forms account for a growing proportion of infections (CRFs = 16.7% by 2015) (7). HIV-1 diversification was already manifested by 1960 as the few partial *env* nucleotide sequences corresponding to subtypes A1 and D showed a distance of 11.7% (8). By 2001, when more sequences were available and 11 CRFs had been documented, median within-subtype amino acid (AA) distances were 8% (range 2%–15%) in Gag and 17% (4%–30%) in Env, while the median distances between subtypes A and B were 17% (15%–22%) in Gag and 25% (20%–36%) in Env (9). The larger distance in Env compared with that in Gag is reflected across analyses: within individuals, within subtypes, and across subtypes. Gag and Pol are more conserved while Env and non-structural proteins are more variable.

HIV-1 is a rapidly evolving virus due to a combination of features: high substitution rates (caused by a low fidelity polymerase), a strong propensity to recombine, and rapid turnover rates (1–2 days) combined with a relatively large population size and the life-long duration of infection in PLWH (10). HIV-1 has been shown to adapt in response to host immune, antiretroviral, and vaccine pressure (11–14). The rapid development of drug resistance mutations that was observed when monotherapies became available in the 1990s has been mitigated by the use of a combination of antiretrovirals, although drug resistance mutations have increased in some populations (15). For T cell immune responses, the observation of T cell escape mutations at the individual level (12) was followed by evidence of HIV-1 adaptation at the population level with HLA-associated polymorphisms spreading in populations where a given HLA is more frequent (16–18), leading to partially predictable HIV-1 escape pathways (19). Similarly, for B cell immune responses, the co-evolution between HIV-1 and neutralizing antibody responses observed at the individual level (20) translates to HIV-1 adaptation to B cell immunity at the population level with an increase in resistance to antibody neutralization observed for recent isolates when compared with HIV-1 isolates from the 1980s (21). Thus, HIV-1 diversification patterns reflect the impact of common HLA alleles, while Env evolution is also imprinted by B cell-specific responses.

Assessing global trends in HIV-1 diversification and site-specific adaptations among circulating strains is critical for designing optimized vaccines and reagents for HIV-1 studies. Recent molecular epidemiology studies systematically evaluated the proportion of each subtype and CRF globally and regionally and shifts in these proportions over time (6, 7). Linchangco and colleagues at the LANL HIV database, which maintains curated data sets of HIV-1 sequences (among other resources), recently updated the consensus for all HIV-1 subtypes and CRFs using 3,470 high-quality, representative HIV-1 genomes selected from the LANL HIV database 2019 filtered web alignments (22). They showed that an average of 2.3% (range 0.8%–10%) of sites changed across the consensus genomes designed in 2002 and in 2021 (with similar proportions of substitutions vs. insertions or deletions) and concluded that 2021 consensus sequences were good representations of the typical subtype/CRF genomes. Similar to the study by Linchangco and colleagues (on genome nucleotide sequences), we analyzed HIV-1 diversification globally and generated consensus sequences. We focused on prominent subtypes and CRFs and considered three specific proteins—Env, Gag, and Pol—which are key targets for interventions and for which more sequences are available than for genomes. Then, we compared patterns across decades by deriving consensus sequences that were specific to a given time period (between 1980 and 1990, between 1991 and 2000, between 2001 and 2010, or between 2011 and 2020) to estimate the scope and properties of HIV-1’s characteristic diversity between 1980 and 2020. Our consensus sequences from the past decade make proteins that can be used as reagents reflecting contemporary HIV-1 sequences. Our open-source platform, Hervé, integrates data and bioinformatic tools for exploring subtype/CRF diversity and is accessible to scientists and the public.

## RESULTS

### Hervé: an open-source platform for exploring HIV-1 sequence diversity

**Fig 1:**
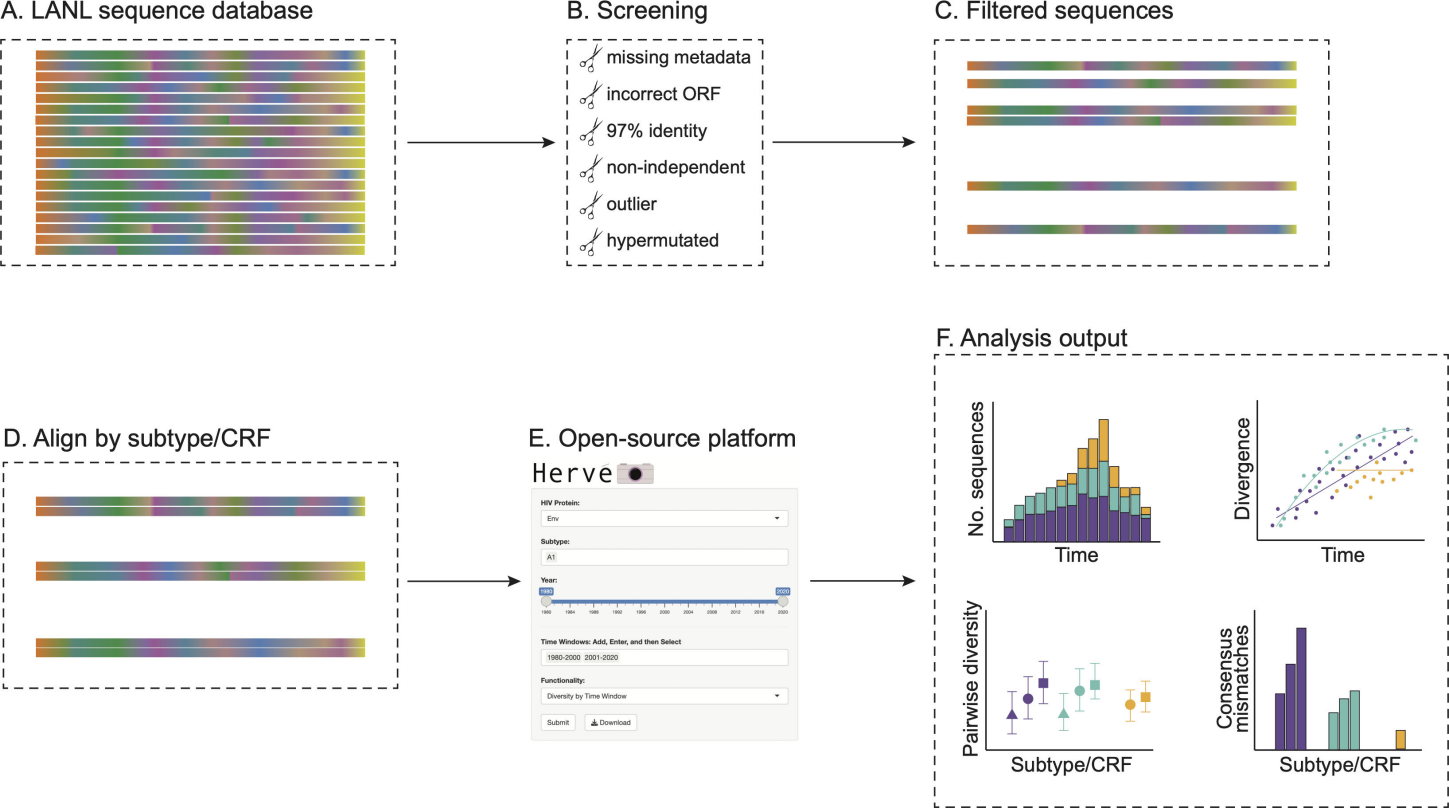
Workflow for the Hervé platform. (**A**) Protein sequences were downloaded from the Los Alamos National Laboratory HIV sequence database. (**B**) The downloaded sequences were passed through data-filtering criteria (see Fig. S1) until a subset of (**C**) complete and independent sequences remained. (**D**) The subset of filtered sequences was aligned by subtype or CRF. (**E**) Each alignment and associated metadata were analyzed on the Hervé open-source web platform. (**F**) Results and visualizations were output.

We developed an open-source platform, Hervé, to analyze and visualize HIV-1 protein sequence diversity (https://hiv1.shinyapps.io/Herve/). The database compiles Env, Gag, and Pol amino acid sequences sourced from LANL and metadata relating to sequence subtype/CRF, sample date, and sample country (Fig. 1). The platform was coded in R and provides a suite of tools for analyzing temporal trends in sequence sampling, estimating pairwise diversity and divergence from the most recent common ancestor (MRCA) over time, calculating amino acid substitutions per site per year, reconstructing consensus sequences for user-specified sampling time windows, and comparing consensus sequences reconstructed for different sampling time windows and subtypes/CRFs. These analyses can be plotted in dynamic formats that allow users to visualize aspects of HIV-1 subtype/CRF diversity, including the exploration of phylogenetic trees. Consensus and MRCA sequence alignments and results tables relating to subtype/CRF diversity analyses can be downloaded. Tutorials for using each tool are included on the platform to help users navigate the suite and interpret their results. The sequence and metadata will be updated annually to account for new sequences deposited in LANL.

### A large and representative data set of Env, Gag, and Pol sequences sampled between 1980 and 2020

**Fig 2:**
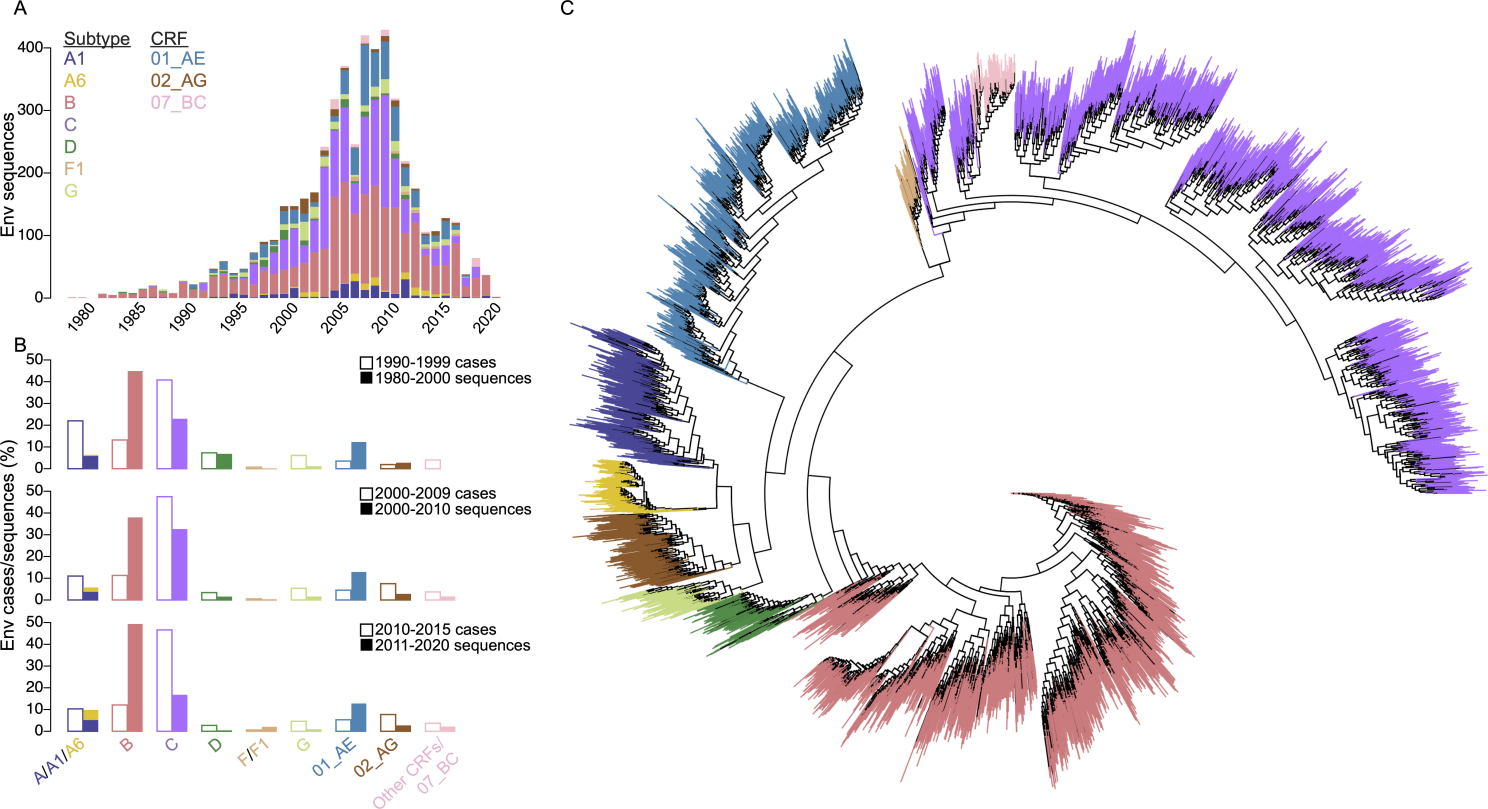
HIV-1 Env subtype and CRF sequences over time. (**A**) The number of Env sequences in our data set sampled between 1980 and 2020 by subtype and CRF. (**B**) The number of PLWH with a given subtype/CRF (as a percentage of all PLWH) and number of Env sequences of that subtype/CRF (as a percentage of Env sequences of all subtypes/CRFs in the data set) in our data set between 1990–1999, 2000–2009, and 2010–2015. (**C**) The reconstructed Env phylogeny with tips colored by subtype and CRF.

Our data collection yielded 34,944 eligible AA sequences sampled between 1980 and 2020. After screening for independent sequences that had a functional open reading frame, 4,830 Env, 4,407 Gag, and 3,002 Pol sequences remained from subtypes A1, A6, B, C, D, F1, and G and CRFs 01_AE, 02_AG, and 07_BC sampled from 82 countries (Table S1; Fig. S1). Across proteins, sequences corresponded to subtypes B (24.9%–41.9%), C (24.3%–27.6%), CRF01_AE (13.2%–19.9%), A1 (5.0%–6.6%), CRF02_AG (3.2%–3.7%), D (2.5%–3.8%), A6 (1.3%–2.1%), 07_BC (1.8%–4.1%), G (1.5%–1.8%), and F1 (1.1%–1.2%) (Fig. S2 to S4). For analyses, sequences were split into four sampling periods: 1980–1990 (2.3%–3.1% of sequences), 1991–2000 (11.0%–16.1%), 2001–2010 (55.8%–60.8%), and 2011–2020 (20.0%–30.9%) (Fig. 2A; Fig. S5A and S6A). We analyzed the numbers of PLWH and compared the proportion corresponding to each subtype/CRF (6) with the proportion of sequences in our data set for a given subtype across time periods. Across proteins, sequence data sets were biased toward subtype B and against subtype C. For Env, the sequence data set included 41.9% of subtype B sequences while only 11.3%–13.2% of PLWH had subtype B infections. Conversely, subtype C represented 27.6% of our sequences but 40.8%–47.5% of PLWH had subtype C infections (Fig. 2B). On average, the difference between the proportion of sequences in our data set and the proportion of documented cases for a subtype/CRF was smaller: ±4.81% for Env, ±3.72% for Gag (Fig. S5B), and ±4.58% for Pol (Fig. S6B).

Phylogenies were constructed with the best-fit models inferred using IQ-TREE 2 (23) and ModelFinder (24): the HIV between-patient matrix for Env (Fig. 2C; File S1) and Pol (Fig. S6C;File S3) and the Jones-Taylor-Thornton matrix for Gag (Fig. S5C; File S2), all with the FreeRate model (25) with 10 classes. Subtype and CRF sequences were segregated into monophyletic clades, except CRF07_BC, which was nested in subtype C, as seen in Env, Gag and Pol, and A6 which was nested in A1 in Pol. One recombination breakpoint was identified in the Env alignment between HXB2 sites 442 and 475, in Gag between 150 and 302, and in Pol between 126 and 683. The distribution of subtypes/CRFs varied across continents. While subtype B has been dominant in different continents (Americas, Europe, and Australia), other subtypes/CRFs are principally represented in specific regions, e.g., subtype G and CRF02_AG in West Africa, subtype C in Southern Africa and India, and CRF01_AE in Southeast Asia. Nonetheless, globalization is blurring early geographic distinctions with subtypes A1, B, C, D, and G and CRFs 01_AE and 02_AG each represented in Africa, the Americas, Asia, and Europe (Fig. S7).

### Divergence from the MRCA has leveled off since 2000 in Env subtype B

**Fig 3:**
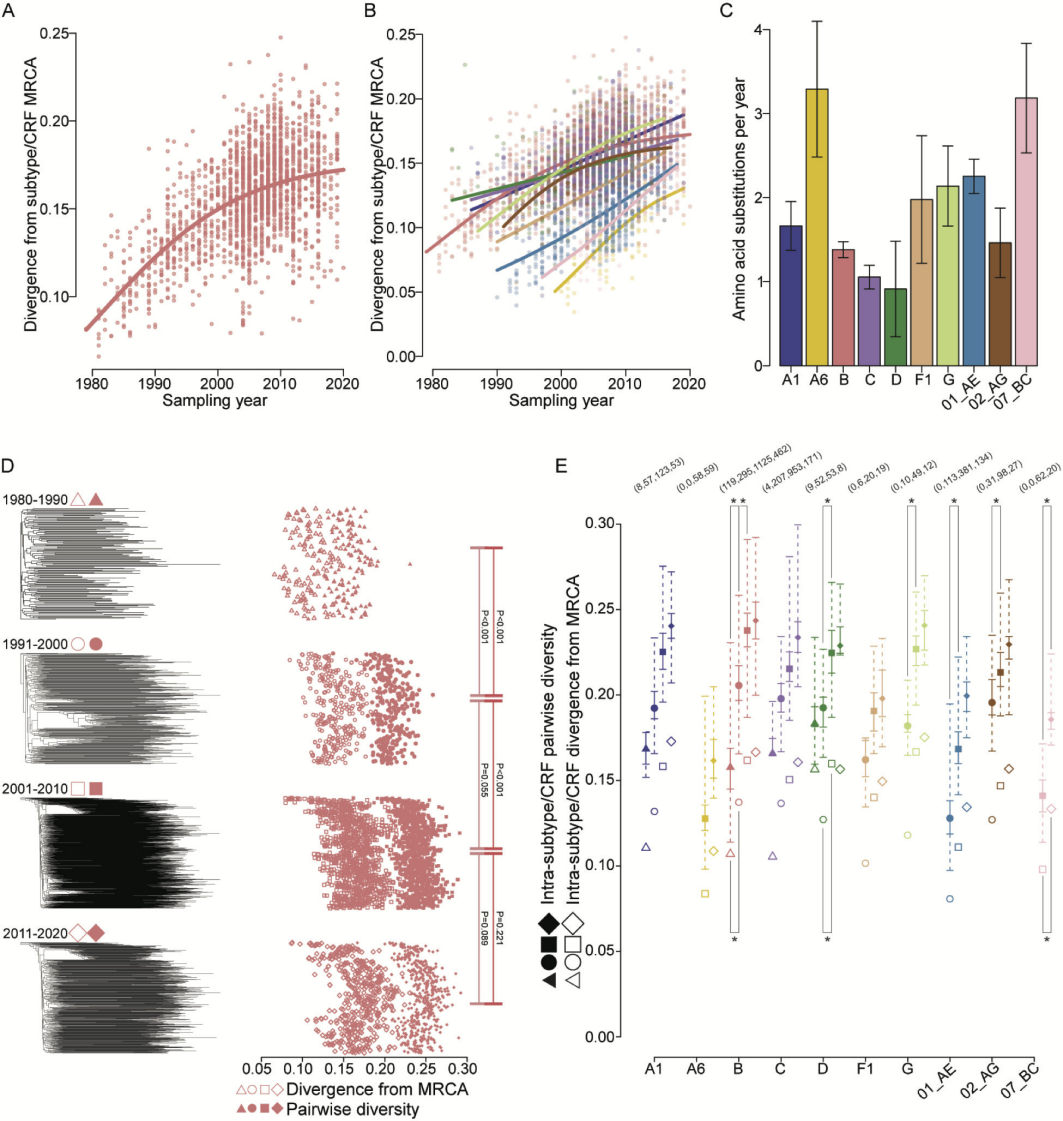
Subtype/CRF divergence and diversity in HIV-1 Env. (**A**) Divergence of each subtype B Env sequence from the subtype B MRCA by sampling year. A best-fit curve is shown. (**B**) Best-fit curves for each subtype/CRF for divergence from each subtype/CRF MRCA by sampling year. (**C**) Amino acid substitutions per year for each subtype/CRF with 95% confidence intervals estimated from a linear regression of the median uncorrected hamming distance per year. (**D**) Reconstructed phylogenies for subtype B Env sequences sampled during each sampling period; the divergence from the MRCA (open shapes) and median pairwise diversity (filled shapes) for each sequence corresponding to a tip in the phylogeny. P-values for Mann-Whitney U tests of divergence from the MRCA (lighter lines) and pairwise diversity (darker lines) between sampling periods are shown. (**E**) Median divergence from the MRCA (open shapes) and median pairwise diversity (filled shapes) for each subtype/CRF at each sampling period. Solid bars indicate IQR1 and IQR3 and dashed bars indicate minimum and maximum values of pairwise diversity. Pairwise significant differences (Mann-Whitney U test, *P* < 0.05) between sampling periods of divergence from the MRCA (below) and pairwise diversity (above) and the number of sequences corresponding to each subtype/CRF for sampling periods are shown.

We computed sequences for the MRCAs of each subtype and CRF and fitted growth models to the divergence of sequences from the MRCA as a function of sampling year. Best-fitted growth models varied between gamma and normal across proteins for each subtype/CRF. For subtype B Env, divergence was best fit by a gamma growth model corresponding to an average of 1.39 AA substitutions per year (s/y) and a projected maximum median pairwise AA divergence of 0.179, projecting that median divergence will increase 0.021 (17.9%) before reaching equilibrium (Fig. 3A). Subtype B Gag and Pol were also best fit with gamma models corresponding to an average of 0.52 (Fig. S8A) and 0.69 (Fig. S9A) AA s/y, respectively. In total, 4/9 subtypes/CRFs were best fit by a normal model in Env (Fig. 3B, Table S2), 5/9 in Gag (Fig. S8B), and only 4/9 in Pol (Fig. S9B). The median number of AA s/y was 1.82 (min = 0.913, max = 3.29) across Env subtypes/CRFs (Fig. 3C), whereas the median number of AA s/y was considerably lower in Gag (0.297, 0.170, and 0.859) (Fig. S8C) and Pol (0.779, 0.607, and 0.920) (Fig. S9C). The projected maximum median pairwise AA divergence inferred for Env subtypes/CRFs best fit by a gamma growth model estimated an average increase in divergence of 0.080 (28.2%), ranging from 0.021 (17.9%) in B to 0.217 (60.4%) in CRF01_AE. Projected average increases were lower in Gag (0.009%, 10.5%) and in Pol (0.014%, 14.2%). This suggests that, for most subtypes, median Env MRCA divergence will increase its current value by 28% before reaching maximum median divergence, whereas Gag and Pol divergence will increase by only 10%–15%. Best-fit models on the full alignment were retrieved 75% (SD 15%) of the time when alignments were downsampled by 50% (Fig. S10). Additionally, we calculated the root-to-tip distance for Env, Gag, and Pol phylogenies rooted on the MRCA and fitted growth models to the root-to-tip distance as a function of sampling year. We found that these model fits were generally concordant with those fitted to sequence divergence (Fig. S11). In particular, subtype B Env and Gag were both fit with gamma models and demonstrated a plateauing toward the present, whereas B Pol showed a linear increase.

We then estimated, for each sampling period, the divergence from the MRCA and the pairwise diversity between sequences. Both the divergence from the MRCA and the intra-subtype/CRF diversity increased over time across subtypes (Fig. 3; Fig. S8 and S9). For subtype B Env, the median divergence from the MRCA was significantly smaller (Mann-Whitney U test, *P* < 0.001) for 1980–1990 (median = 0.107, IQR = 0.094–0.123) compared with 1991–2000 (0.139, IQR = 0.126–0.156), but there was no significant difference between 1991–2000 and 2001–2010 (0.163, IQR = 0.149–178) or between 2001–2010 and 2011–2020 (0.168, IQR = 0.151–0.184) (Fig. 3D); pairwise diversity was significantly smaller in 1980–1990 (0.158, IQR = 0.145–0.169) compared with 1991–2000 (0.206, 0.197–0.217), as was 1991–2000 compared with 2001–2010 (0.238, IQR = 0.228–0.249), but there was no difference between 2001–2010 and 2011–2020 (0.244, IQR = 0.233–0.254). Similar patterns were seen for subtype B Gag and Pol (Fig. S8D and S9D). Across other subtypes/CRFs, median MRCA divergence was only significantly different between 1991–2000 and 2001–2010 for subtype D (*P* < 0.001) and pairwise diversity increases were significant between 1991–2000 and 2001–2010 for subtypes D and G and CRFs 01_AE and 02_AG (*P* < 0.01); both MRCA divergence and pairwise diversity were significantly higher in 2011–2020 than in 2001–2010 for CRF07_BC (Fig. 3E). Significant differences between time periods were more common for Gag (Fig. S8E) and Pol (Fig. S9E). There was a significant positive relationship between the number of new infections per year in the Americas, where subtype B is dominant, and the rate of change of B MRCA divergence (*R*^2^ = 0.513, *P* < 0.001), but no such relationship between new infections in the Republic of South Africa (RSA), where subtype C is dominant, and the rate of change of C MRCA divergence (*P* = 0.605) (Fig. S12). The fact that the number of new infections declined in both countries while only subtype B divergence plateaued suggests that the rate of decline of new infections cannot fully explain the decrease in divergence for subtype B. Iteratively downsampled Env alignments for subtypes B, C, and CRF01_AE demonstrated a minor effect of undersampling on intra-subtype/CRF diversity estimates: the mean of median (i.e., the mean of all the median estimates calculated for each subtype/CRF) difference between pairwise diversity for the downsampled alignments and the full alignment was 0.001–0.004 at 5% and 0 at ≥15%–35%, and the standard deviation of the difference between pairwise diversity for the downsampled alignments and the full alignment was 0.006–0.012 at 5% and <0.001 at ≥50%–75% (Fig. S13).

### Variance in Env consensus sequences over time

We generated consensus sequences for subtypes/CRFs for each sampling period, except when there were fewer than 10 sequences available (Table S2; File S4 to S6). We compared our derived consensus sequences with the consensus sequences available at LANL: the recently generated LANL 2021 consensus sequences (based on 3,470 high-quality, representative HIV-1 genomes selected from the 2019 filtered web alignment) (22) and the previously available LANL 2004 consensus sequences (similarly derived from a filtered set of sequences available at the time). Compared with the LANL 2004 Env consensus sequence, our consensus sequences showed a mean of 37 (only subtype B), 30.29 (min = 11, max = 49), 33.50 (18,52), and 35.86 (27,43) mismatched residues between the consensuses we generated for 1980–1990, 1991–2000, 2001–2010, and 2011–2020, respectively, which decreased to 24, 26 (17,33), 24.80 (11,40), and 30.33 (15,46) when compared with the new LANL 2021 consensus sequences (11) (Fig. S14). Importantly, most of the mismatches were at variable sites, where the consensus residue represented on average 59.48% for LANL 2004 and 59.51% for LANL 2021 of sequences in the subtype/CRF alignment (Fig. S14B and D). Considering only sites where the consensus residue represented >50% of sequences, a mean of 6, 8.86 (1,17), 10.7 (2,24), and 15.11 (1,27) sites were mismatched with the LANL 2004 consensus for 1980–1990, 1991–2000, 2001–2010, and 2011–2020, respectively, and 5, 7.71 (3,12), 5.9 (0,12), and 8.67 (0,18) with the LANL 2021 consensuses. Across Gag and Pol consensuses over all sampling periods, there was a mean of 5–11.25 (4.5–6.75 at sites with >50% consensus) and 1.25–9.75 (1–17.5) mismatched residues, respectively, with the LANL 2004 consensuses, and 3–11 (1.83–11) and 0.63–3.75 (1–4.75) when compared with the LANL 2021 consensuses (Fig. S15 and S16). The mean percentage for the consensus residue at mismatched sites was 45.24% in the subtype/CRF alignments for Gag and 44.4% for Pol.

**Fig 4:**
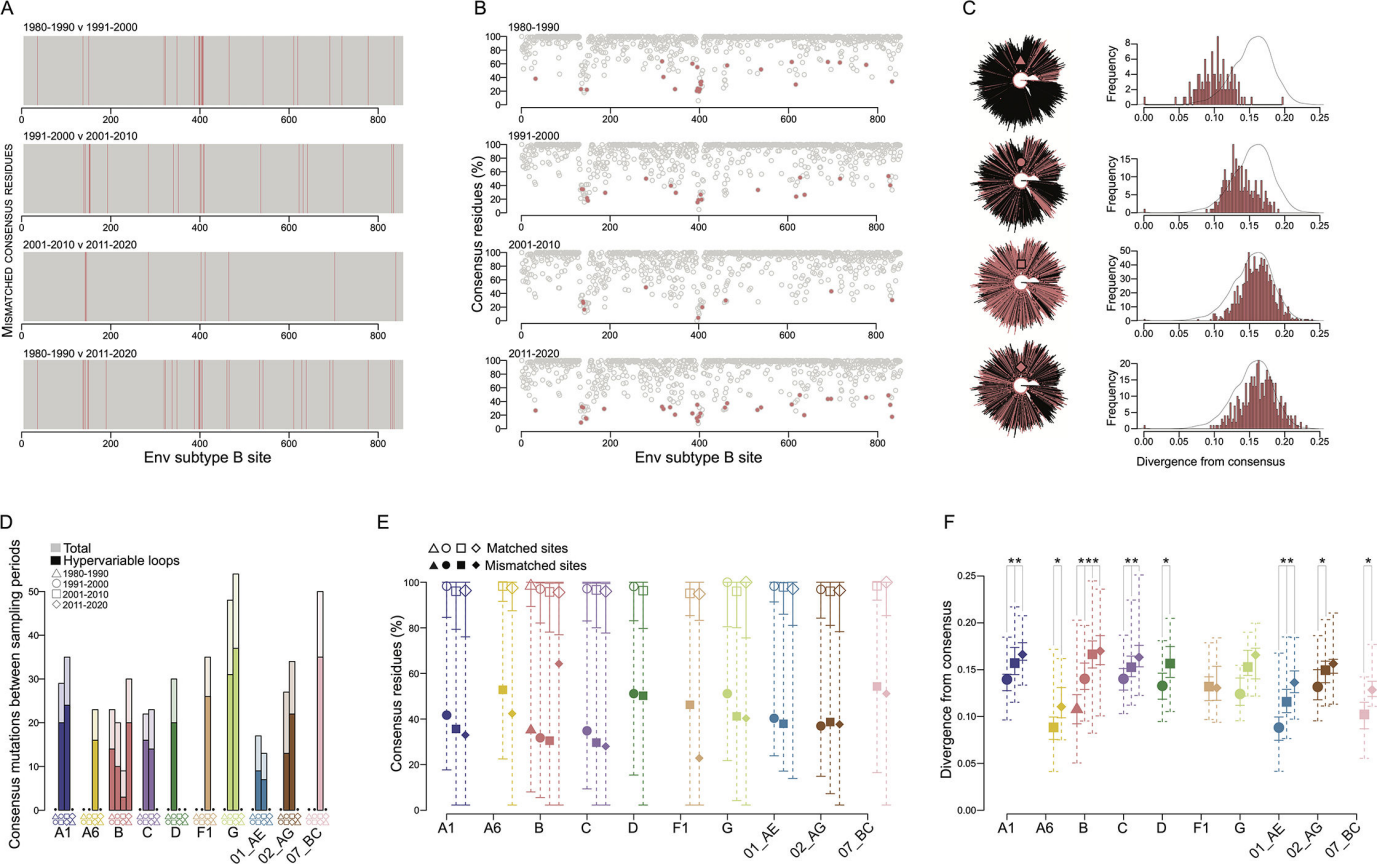
Subtype/CRF mutations over time in HIV-1 Env. (**A**) Subtype B Env sites mismatched (red lines) between consensuses from sequential sampling periods and (bottom) between the first and last sampling periods. (**B**) The percentage of consensus residues at each site in the subtype B Env alignment for each sampling period. Sites mismatched between consensuses (see panel A) are shown in red. (**C**) Phylogeny of subtype B Env sequences with branches colored red for sequences sampled in (top to bottom) 1980–1990, 1991–2000, 2001–2010, and 2011–2020, with the position of the consensus sequence for each sampling period noted; corresponding histograms of divergence from the consensus at each sampling period (red) and a density plot of divergence across all sampling periods (black line). (**D**) Bar plots of the number of sites with mismatched residues between sampling periods for each Env subtype/CRF; shapes in each row below bars indicate the two sampling periods compared. Black dots are shown when the consensus was not calculated due to too few sequences. (**E**) The percentage of consensus residues, as measured from the sampling period consensus, at all sites matched (open shapes) or mismatched (filled shapes) between consensus sequences. Open shapes indicate median values, solid bars indicate IQR1 and IQR3 for matched sites, and dashed bars indicate minimum and maximum values for matched sites; closed shapes indicate median values for mismatched sites; shapes correspond to sampling periods in panel D. (**F**) Divergence from the consensus at each sampling period; solid bars indicate IQR1 and IQR3, dashed bars indicate minimum and maximum values; significant pairwise differences between sequential sampling periods (shapes correspond to sampling periods in panel D) are indicated with asterisks (Mann-Whitney U test, *P* < 0.001). Subtype/CRF consensuses were only calculated for sampling periods with at least 10 available sequences.

Looking at variation in subtype B Env over time, there were 23 mismatched residues between consensuses for 1980–1990 and 1991–2000, 20 between 1991–2000 and 2001–2010, and 9 between 2001–2010 and 2011–2020, amounting to 30 mismatches between 1980–1990 and 2011–2020 (Fig. 4A). Sites that differed over time across consensus sequences were variable: consensus residues at mismatched sites represented 27.7%–62.2% of residues at those sites compared with 95.0%–97.4% at sites conserved in consensus sequences over time (Fig. 4B). When all the sequences from a sampling period were compared with their consensus, divergence from the consensus increased significantly (Mann-Whitney U test, *P* < 0.001) from 0.101 (IQR = 0.087–0.118) in 1980–1990 to 0.135 (0.123–0.151) in 1991–2000 to 0.161 (0.146–0.175) in 2001–2010, but then only marginally (*P* = 0.081) to 0.164 (0.150–0.181) in 2011–2020 (Fig. 4C). For all subtype/CRF Env consensuses, the average number of mismatches between consensus sequences for 1991–2000 and 2001–2010 was 27.57 (min = 17, max = 48) and between 2001–2010 and 2011–2020 was 30.67 (9,54) (Fig. 4D). An average of 21.1%–62.2% of residues at mismatched sites was conserved compared with 97.1%–98.1% at matched sites (Fig. 4E). Compared with Env, Gag and Pol consensus sequences remained more stable over time. For Gag, there were 3–18 mismatched sites between consensuses for 1980–1990 and 1991–2000, 2–10 for 1991–2000 and 2001–2010, and 1–18 for 2001–2010 and 2011–2020 (Fig. S17A) and the percentage of consensus residues at mismatched sites was 17.5%–78.6% compared with 91.7%–96.5% at matched sites (Fig. S17B). For Pol, there were four mismatched sites between consensuses for 1980–1990 and 1991–2000, 1–32 for 1991–2000 and 2001–2010, and 3–20 for 2001–2010 and 2011–2020 (Fig. S18A) and the percentage of consensus residues at mismatched sites was 42.3%–88.5% compared with 94.7%–97.8% at matched sites (Fig. S18B). Similar to patterns in Env of MRCA divergence and pairwise diversity at each sampling period, the AA divergence from the Env consensus increased across subtypes/CRFs, on average, by 18.72% (IQR = 13.59%–21.89%) between 1991–2000 and 2001–2010 and by 8.72% (IQR = 6.12%–26.42%) between 2001–2010 and 2011–2020 (Fig. 4F). In Gag, median divergence increased by 38.16% (IQR = 36.18%–86.27%) between 1980–1990 and 1991–2000, 25.14% (IQR = 23.60%–27.38%) between 1991–2000 and 2001–2010, and 11.66% (IQR = 9.24%–12.47%) between 2001–2010 and 2011–2020 (Fig. S17C); and in Pol, median divergence increased by 46.70% (only subtype B), 26.46% (IQR = 19.63%–39.22%), and 16.06% (IQR = 9.17%–21.09%) for the same time periods (Fig. S18C).

### Limited evidence of global HIV-1 adaptation over time

**Fig 5:**
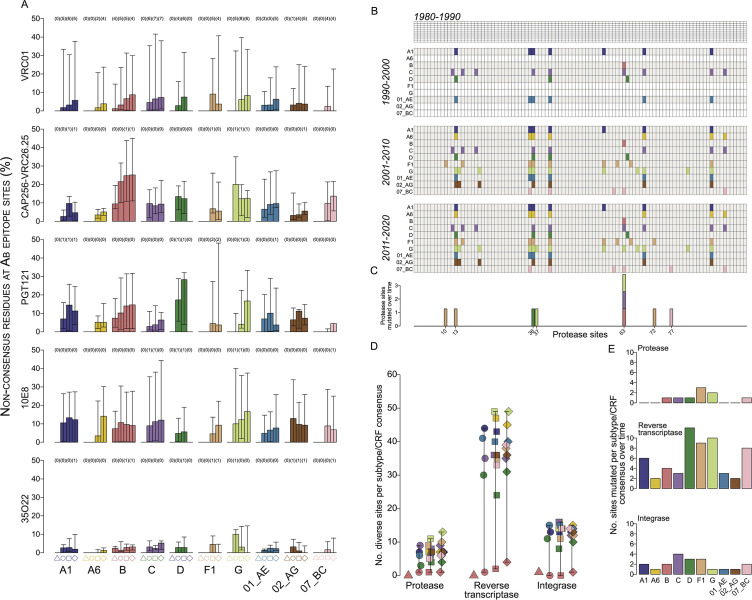
Limited global HIV-1 adaptation over time in HIV-1 Env and Pol. (**A**) Bar plots of the median percentage of non-consensus residues within each subtype/CRF for 1980–1990 (triangle), 1991–2000 (circle), 2001–2010 (square), and 2011–2020 (diamond) at VRC01, CAP256-VRC26.25, PGT121, 10E8, and 35O22 Ab epitope sites; bars indicate IQR1 and IQR3, the number of ambiguous sites (<50% consensus) is shown parenthetically. (**B**) Map of mismatched residues between the reference protease (PR) sequence and each subtype/CRF for each sampling period. (**C**) The number of subtypes/CRFs with a mutation across sampling periods at a PR site. (**D, E**) The number of PR, reverse transcriptase (RT), and integrase (IN) sites that showed (**D**) mismatches with the reference sequence and (**E**) changes over sampling periods in each subtype/CRF at each sampling period. Subtypes/CRFs are colored according to panel A; shapes correspond to sampling periods in panel A.

We wanted to test if diversification occurred at sites of functional importance. First, we analyzed epitopes for antibodies representative of key Env targets. There were few mismatched sites over time between Env consensuses at Ab epitope sites. For subtype B, between 1980–1990 and 1991–2000, there were no mismatched sites for the 36 sites in the VRC01 epitope, 0 for CAP256-VRC26.25 (*n* = 6), 1 for PGT121 (*n* = 13), 0 for 10E8 (*n* = 10), and 0 for 35O22 (*n* = 12). On average across subtypes/CRFs, the number of sites with mismatches between 1991–2000 and 2001–2010 and between 2001–2010 and 2011–2020, respectively, for VRC01 was 1.71 (max = 4) and 1.78 (6), for CAP256-VRC26.25 was 0.29 (2) and 0.22 (1), that for PGT121 was 0.14 (1) and 0.44 (2), that for 10E8 was 0.43 (2) and 0, and that for 35O22 was 0 and 0.22 (1) (Fig. S19 to S23). Nonetheless, diversity at Ab epitope sites increased slightly over time. The mean of median percentage represented by non-consensus residues at Ab epitope sites within each subtype/CRF increased on average by 4.92% (max = 9.1%) for VRC01, 9.1% (15.3%) for CAP256-VRC26.25, 8.8% (28.3%) for PGT121, 7.8% (13.3%) for 10E8, and 2.1% (4.5%) for 35O22 (Fig. 5A). Across sampling periods, the average number of ambiguous Ab epitope sites (i.e., <50% consensus) was 3.5 (min = 1.0, max = 6.5) at VRC01 sites, with an average addition of 4.0 ambiguous sites between the first and last sampling periods, but ≤1 site in other Ab epitope groups. Importantly, the consensus residue changed over time only at a few Ab epitope sites, which for subtypes B, C and CRF01_AE included: N279D in subtypes B and C, T461N in subtype C, N462T in subtype B, and N463T in CRF01_AE in VRC01; and T308R in CRF01_AE and T319A in subtype B for PGT121 (for other subtypes/CRF: Table S3 to S7).

Second, we evaluated diversification at Pol sites associated with drug resistance mutations. Within the Pol region that encodes the PR enzyme, the median numbers of sites mismatched with the PR reference per subtype/CRF consensus were 0 for 1980–1990, 2.6 (max = 9) for 1991–2000, 6.5 (11) for 2001–2010, and 7.0 (13) for 2011–2020 (Fig. 5B), with one site each in subtypes B, C, and D and CRF07_BC and two sites in subtype G changed between the first and last sampling periods (Fig. 5C). Between 1980 and 1990, zero RT and one IN site were mismatched in subtype B and the median numbers of mismatched RT and IN sites, respectively, across subtypes/CRFs, were 15.1 (max = 44) and 5.4 (15) for 1991–2000, 34.5 (33) and 12.1 (16) for 2001–2010, and 5.7 (33) and 11.8 (15) for 2011–2020 (Fig. 5D), with an average of 5.9 (12) and 2.0 (4) sites changed between the first and last sampling periods (Fig. 5E; Fig. S24 to S27). Only one site associated with a drug-resistant mutation was mismatched between the reference and a consensus: PR V82I in subtype G in all sampling periods. The mean of the median percentage for each subtype/CRF of non-consensus residues at sites associated with drug resistance mutations was also low: 1.11% (max = 12%) for PR, 0.77% (29.2%) for RT, and 0.03% (20%) for IN across sampling periods.

Third, we analyzed sites corresponding to predicted T cell epitopes for Env, Gag, and Pol consensuses. We predicted cytotoxic T lymphocyte (CTL) epitopes for HLA alleles with >5% frequency in five major human subpopulations for each subtype/CRF. The average number of CTL epitopes across Env subtypes/CRFs was 14.2 for 1980–1990, 9.4 (min = 8.8, max = 14.4) for 1991–2000, 9.6 (7.8,15) for 2001–2010, and 9.4 (7,15.8) for 2011–2020 (Fig. S28A); 4.8 (4.2,5.4), 4.4 (3.6,5.4), 4.4 (2.6,6.4), and 4 (2.6,6) for Gag (Fig. S28B); and 15.8, 14.2 (13,15.8), 13.9 (12.8,16.8), and 14.4 (12,16) for Pol (Fig. S28C). On average, predicted CTL epitopes covered between 18% and 25% of Env proteins, 20% and 23% of Gag, and 25% and 30% of Pol. We then counted the number of mismatched sites between sampling period consensuses occurring inside versus outside of predicted CTL epitopes. For Env (excluding hypervariable regions), 38.5% (5/13) of mismatched sites between subtype B consensus sequences for 1980–1990 and 1991–2000 fell within predicted CTL epitopes; across subtypes/CRFs, an average of 21.5% (min = 5.3%, max = 33.3%) and 25.4% (6.1%, 66.7%) of mismatched sites between consensus sequences for 1991–2000 and 2001–2010 and for 2001–2010 and 2011–2020, respectively, fell within predicted CTL epitopes (Fig. S29A). For Gag, an average of 8.3% (0%, 16.7%) of mismatched sites fell within CTL epitopes for 1980–1990 and 1991–2000, 36.7% (20%, 50%) for 1991–2000 and 2001–2010, and 22.4% (0%, 75%) for 2001–2010 and 2011–2020 (Fig. S29B); and for Pol, the average percentages of mismatched sites within CTL epitopes were 25%, 30.8% (0%, 50%), and 28.8% (0%, 60%), respectively (Fig. S29C). When we compared the percentages of mismatches found within CTL epitopes with the fraction of the corresponding protein that was covered by CTL epitopes, we found no strong evidence that mismatches between consensus sequences over time were preferentially found in CTL epitopes. Across all subtypes/CRFs, the percentage of mismatched sites falling within CTL epitopes in Env was on average 20.15% higher than the fraction of Env covered by CTL epitopes; this ratio was 2% lower for Gag and 27.08% higher for Pol (Fig. S30). However, these values were not significantly different from 0% (one-sided Mann-Whitney U test, *P* > 0.22) and did not typically increase over sampling periods. Therefore, the general lack of increase in consensus mismatches in CTL epitopes over time across subtypes/CRFs suggests that T cell escape was not a strong enough population-wide phenomenon to lead to substantial AA changes in Env, Gag, or Pol consensus sequences.

## DISCUSSION

Our comprehensive sequence analysis of the diversity of structural proteins for HIV-1 subtypes and CRFs included 12,239 Env, Gag, and Pol sequences spanning 1980 to 2020. We found that, between 1980 and 2020, consensus sequences changed by an average of 5.3% AA in Env, 2.1% in Gag, and 2.9% in Pol, while sampled sequences became more distant from the consensus by 61%, 79%, and 98%, respectively. The minimal changes we observed in consensus sequences for Env, Gag, and Pol over time were consistent with a recent analysis of genome nucleotide sequences which used a different approach to consensus construction (22). Our results indicate no qualitative shift in the mutational landscape of structural proteins for the most prominent HIV-1 subtypes/CRFs, despite a marked increase in intra-subtype/CRF diversity, amounting to an accumulation of neutral variation rather than adaptation. The extreme directionless diversification of HIV-1 can be considered as noise for researchers trying to identify targets for vaccine design. This also illustrates that, while using the consensus as a reference or as a reagent for experimental assays is better than using any random isolate (9, 26–28), the consensus masks a considerable genetic space covered by circulating sequences.

Subtype-specific consensus sequences shifted minimally over time (due to limited HIV-1 adaptation to immune pressure at the population level) and correspond to the best single sequence to represent all circulating sequences. Nonetheless, each consensus integrates a continuously increasing array of variation, meaning that each individual sequence has unique features. Restricting consensuses to only the last decade may better capture these changes in circulating diversity. Our 2011–2020 consensuses differ by ~4% from consensuses recently generated by LANL, which include all sequences sampled up to 2021 (22), and thus provide a complementary set for use in HIV-1 research and immunogen design. Roughly 2/3 of these differences reflect sites lacking a majority consensus. We found more modest variability between our and LANL consensuses for Gag (~1%) and Pol (<1%). The accumulation of diversity we observed across sequences presents a growing challenge as consensus sequences must integrate an expanding genetic space. Consensus sequences optimized to reflect circulating HIV-1 diversity are important reagents for studying HIV-1-induced immunity and may help the development of a vaccine against HIV-1. While a prophylactic against infection is likely the only course for ending the pandemic (29), the genetic diversity of HIV-1 is a challenge for eliciting immune responses that are cross-reactive across subtypes/CRFs (9, 26). Most vaccine efficacy trials tested vaccines corresponding to natural isolates typically corresponding to a single clade (subtype B: VAX004 [30], HVTN 502 [31], and HVTN 503 [32] and subtype C: HVTN 702 [33]) or two clades (subtype B and CRF01_AE: VAX003 [34)] and RV144 [35]), reflecting the dominant subtype/CRF circulating in the target population. More recent vaccine trials tested multi-subtype vaccine candidates with either a set of natural strains from subtypes A, B, and C (HVTN 505 [36]) or artificially created immunogens, such as Mosaic designs (37), that may better represent HIV-1 diversity (HVTN 705, HVTN706). Polyvalent mosaic vaccines, which were initially designed as T cell vaccines (38), favor inclusion of common epitopes. These Mosaic vaccine candidates showed promising results in non-human primate challenge studies (39, 40) but failed to induce efficacious protection in humans (https://www.clinicaltrials.gov/study/NCT03964415 and https://www.clinicaltrials.gov/study/NCT03060629). Importantly, our consensus sequences specific for the past decade (thus reflecting contemporary circulating sequences) were successfully expressed as proteins, illustrating that they could be used as updated reagents for experimental assays and as potential vaccine candidates (41).

We found that subtype/CRF sequences diverged from their MRCA at about a threefold higher rate in Env compared with Gag and Pol and rates could vary widely across subtypes. While some differences may be attributed to the limited sampling available for some subtypes, previous work has shown that demographic differences can also influence the diversification of subtypes/CRFs over decades. For example, faster epidemic spread can occur among people who inject drugs or through frequent consecutive transmissions between individuals in acute HIV-1 infection (i.e., more likely to transmit with high viral loads). These rapid transmission chains with near-identical viruses lead to depressed evolutionary rates and would therefore limit diversification over time (42). The age of epidemics has also been shown to affect evolutionary rates (43), as in the relatively young subtype A6 epidemic in South Korea, where an early rapid burst of genetic novelty may have expedited diversification (44). Antiretroviral therapy (ART) has been described as potentially having antagonistic effects on evolution: limiting evolution as ART suppresses viral load and thereby the risk of transmission (45) and accelerating selection for drug resistance mutations (46); however, our sequence analysis did not show evidence of drug resistance mutations spreading sufficiently to be integrated in consensus sequences. In subtype B, Env and Gag divergence plateaued after 2000, while subtypes A1 and C and CRF01_AE saw steady climbs in divergence and diversity up to the present. While the rate of MRCA divergence in subtype B Env sequences and the number of new HIV-1 infections in the Americas have both decreased over time, there was no such relationship for subtype C and new infections in the RSA, suggesting that the decline in the number of new cases is not a sufficient explanation for the plateauing of subtype B divergence after 2000. Subtype B is distinguished from the other subtypes/CRFs by the fact that it had already spread globally by the 1980s. It is possible that the effect of genetic drift has abated as the epidemic has grown leading to a plateau in divergence. Another distinction with other subtypes/CRFs is that widespread access to ART occurred first for subtype B. The control of viral replication at the population level through ART has possibly limited subtype B divergence in the last decade. The plateauing of divergence in subtype B can be reminiscent of the time-dependent rate phenomenon reported for many viruses whereby evolutionary rates are higher when calculated over a short time period than over a longer time period (47, 48). The time-dependent rates reflect the presence of deleterious mutations that inflate short-term rates and are removed over time and the saturation of substitutions which deflates long-term rates. Because our analysis relied on protein sequences, it provided a limited understanding of saturation (which would be improved with the analysis of nucleotide sequences). Evidence seen for subtype B may be related to the fact that sampling of sequences is the most robust for subtype B. However, whether the time-dependent rate phenomenon will be manifested across all HIV-1 subtypes remains to be seen.

The subtle divergence from the MRCA coupled with increased pairwise diversity within subtypes/CRFs we observed led to an expansive exploration of genetic space but with only minor changes in consensus sequences. The paradox of stable consensus sequences despite rapid HIV-1 diversification is reminiscent of studies that showed that the increase in diversity observed at the individual level during an HIV-1 infection did not translate to the population level (49–51). Multiple studies have shown that HIV-1 diversification within an individual is driven by the host immune responses, both T cell and antibody mediated. At the population level, when examining consensus sequences over time, we found limited evidence of HIV-1 Env, Gag, and Pol adaptation to the selective pressure exerted by CTL. The number of mismatched sites between consensus sequences over time did not vary differently within or outside of predicted CTL epitopes. Similarly, there was limited evidence of Env adaptation to selective pressure from antibody responses. Env diversity increased at contact sites for critical bnAbs, yet there were only rare examples of changes in the consensus residue at a bnAb contact site (for subtype B: N279D, T461N, and N463T) and these happened at variable sites. These results contrast with the different studies that showed a decreased susceptibility of circulating sequences to bnAbs over time (21, 52–55). The pattern of evolved resistance appears to be both specific to bnAbs, such as a more than twofold increase in ${IC}_{50}$ for 3BNC117 compared with VRC01 in subtype B (56), and to subtypes/CRFs, with relatively increased resistance to PG9 in subtype C (53). While we could not link diversification patterns to typical sequence features of Ab and CTL pressure, it is possible that the seemingly neutral patterns of accumulating diversity at the population level may mask higher-order evolutionary patterns that our sequence analysis did not uncover.

Relatedly, at the consensus level, we found that only ~6% of Pol sites were mismatched with PR, RT, and IN reference sequences across subtypes/CRFs, while previous work has shown that over a third of PR, RT, and IN sites are variable across subtypes/CRFs (57, 58). More importantly, except for one site in subtype G, we found no evidence of subtype/CRF adaptation at sites associated with drug resistance mutations. This suggests that, while Pol has been slowly diversifying since 1980, the impact of antiretroviral drug selection pressure has remained limited at the population level.

Our study was limited by the underlying available HIV-1 sequences. While the proportion of available sequences by subtype/CRF largely reflected the global case distribution, our data set included a relative excess of subtype B and CRF01_AE and deficit of subtype C sequences, as expected from historical research biases. Additionally, the number of sequences deposited in the LANL HIV-1 sequence database has increased over 10-fold since 2000 (22), resulting in a sample skewed toward the present. Given that the percentage of PLWH who know their HIV-1 status has increased from 5.7% in 2000 to 84% in 2020 in sub-Saharan Africa (59), our sample likely underrepresents older cases. These biases could affect our estimates of intra-subtype diversity as well as our analysis of temporal trends.

Our work underscores the importance of continuing to track HIV-1 subtype/CRF sequence diversity over time. Such analyses are critical to understanding the trajectory of HIV-1 evolution, as well as identifying genetic novelty in outbreaks that may underlie differences in virulence or transmission (60). Our Hervé platform identifies a further need within the community of HIV-1 researchers and the interested public to have an accessible resource for exploring and visualizing HIV-1 diversity, both for tracking up-to-date changes that may have impact on vaccine and therapeutic design and for facilitating the public’s understanding of the ongoing challenges of mitigating the HIV-1 pandemic. Overall, our work shows largely unchanged consensus sequences over time despite the extensive non-directional diversification of HIV-1. This apparent contradiction illustrates the tension between neutral evolution and selection in HIV-1, with strong selective processes within host that do not translate similarly to the population level.

## MATERIALS AND METHODS

### Design and construction of a bioinformatic platform

The Hervé platform was written in Shiny R code using an RStudio Desktop integrated development environment version 2022.07.2 and R version 4.2.1.

The Hervé architecture includes server.R, ui.R, and R scripts defining each functionality. There are three input parameters (input, output, and session) to server.R. The backend R session is programmed to receive users’ input values through the user interface by the Event handler to trigger an R script defining a functionality. Input data are stored as fasta files and RData files. Output data are stored in a www folder and assigned a session token to avoid conflicting file names during simultaneous use. Graphics are displayed directly to the user interface by renderPlot. A download button triggers the Event to server.R to zip sequence alignments, tables, and graphics corresponding to the output of each functionality. The user interface includes a tutorial for navigating each functionality.

### Data collection and eligibility criteria

Env, Gag, and Pol AA alignments were retrieved from the Los Alamos National Laboratory HIV-1 sequence database on 7 November 2022 using the “one sequence per participant” download criteria. A sequence was retained if it (i) corresponded to a complete ORF, (ii) included a time stamp and country information, (iii) was not hypermutated or problematic (as defined by LANL), and (iv) was not sampled as part of a vaccine trial. Sequences were then removed if fewer than 100 sequences were available from that subtype/CRF for any protein.

Since HIV-1 is a chronic infection, some participants have been followed for up to 10 years and sequences can be available at multiple time points and can differ by a few percentage points, reflecting intra-host evolution. A sequence was removed if it had >95% identity for Env, 97% identity for Gag, or 98% identity for Pol with an earlier-sampled sequence. To confirm that these thresholds were sufficient to remove linked sequences, we plotted the distribution of pairwise sequence identity prior to deduplicating and counted the number of sequences in each subtype/CRF that exceed a 95% threshold in Env, 97% threshold in Gag, and 98% threshold in Pol (Fig. S31). We calculated median pairwise diversity for sequence subsets that corresponded to identity thresholds ranging between 90% and 99% (i.e., after removing sequences that shared higher identity than the threshold with another sequence in the set) to estimate whether the threshold differences affected median pairwise diversity estimates (Fig. S32 to S34).

Of the remaining data set, any hypermutated sequences not identified by LANL were removed. Gap-only columns were removed from the curated subtype alignments.

### Consensus and ancestral sequence generation

For each protein and subtype/CRF, we generated consensus sequences for samples between 1980–1990, 1991–2000, 2001–2010, and 2011–2020 (inclusive). Each consensus was determined by the most common residue at each site, and ties were broken in the order of frequency in HIV-1 sequences: LGIKEAVTRQSPNDWYFHCM (https://www.hiv.lanl.gov/content/sequence/CONSENSUS/AdvCon.html). Sites with >50% gaps in the subtype/CRF alignment were excluded. Ten sequences were required for generating a consensus. For comparisons, we used consensus sequences generated by the LANL HIV database and available at https://www.hiv.lanl.gov/content/sequence/NEWALIGN/align.html. These consensus sequences are labeled 2021 and 2004 (reflecting the year they were derived) and are based on a high-quality selection of the filtered web alignments of HIV-1 genome sequences.

Ancestral sequences were reconstructed at all internal nodes using FastML v.3.11 with branch-length optimization and a gamma distribution (61) for each protein and subtype/CRF. Differences between circulating sequences and the sequence of the MRCA were determined using the AA reconstructions with the highest marginal probability at each AA site.

### Diversity estimates within and between subtypes and CRFs

Intra-subtype/CRF diversity was estimated using the pairwise distance between all sequences within a subtype/CRF, between all sequences and the MRCA, and all sequences and the consensus. Pairwise diversity was calculated with the HIV-1 between-individual amino acid substitution model based on an HIV-1 subtype B-specific amino acid substitution rate matrix as determined in reference 62 in the R package *phangorn* (63). The percentage of residues in subtype/CRF alignments matching the consensus was counted at all sites, excluding gaps. Pairwise diversity and consensus residues were computed on sequences sampled from the same sampling period (1980–1990, 1991–2000, 2001–2010, or 2011–2020). Data on new HIV-1 infections in the Americas and the RSA were sourced from https://datacatalog.worldbank.org/search/dataset/0037712/World-Development-indicators. The rate of change of the number of new infections per year in the Americas and the RSA were regressed against MRCA divergence for subtypes B and C.

To assess the effect of sample size on diversity estimates, subtype B, C, and CRF01_AE sequences were iteratively downsampled and the pairwise distances were calculated on the downsampled alignments. Between 5% and 95% of sequences were randomly sampled without replacement, and pairwise distances were calculated from the downsampled sequences. This process was repeated 100 times to get an average of the difference from the pairwise distance using full alignments.

### Phylogenetic analysis

Subtype/CRF alignments were aligned to each other one at a time, beginning with subtype B, to construct an alignment for each protein that included all subtypes/CRFs. Alignments were done using MAFFT v7.475 (64), and recombination breakpoints were identified using GARD (65), which searches for phylogenetic incongruence among partitions of the alignment. For each protein, a phylogeny was constructed with IQ-TREE 2 (23) using ModelFinder (24) to identify the best-fit model selected based on a minimal Bayesian information criterion. When recombination breakpoints were identified, we used a partition model with proportional branch lengths to allow for separate substitution rates in non-recombinant regions.

### Divergence estimates of subtypes and CRFs

For each protein and subtype/CRF, pairwise distances between the MRCA and each sequence (i.e., divergence) were calculated with a HIV-1 between-participant matrix (62) and empirical base frequencies using the R package phangorn (63). Root-to-tip distances were calculated on subtype/CRF phylogenies rooted on the MRCA sequence using the R package adephylo (66). Non-linear least-squares parameter estimation was used to compute the growth rate of divergence estimates as a function of sampling time and the carrying capacity inferred by the model, which here was defined as the projected maximum median divergence estimate. Univariate distributions for normal, gamma, and exponential models were fit to each data set using maximum likelihood estimation; best-fit models were determined by likelihood ratio tests. To assess the ability of model fitting, divergence data were simulated under normal (α=0.01-0.1), gamma (κ=0.5-10, θ=1.5), and exponential (β=0.05-0.1) distributions and best-fit models were recovered as above (Fig. S35). To assess the effect of the number of sampled sequences on accurate model recovery, we iteratively downsampled 100 times each subtype/CRF alignment by 10%–50% and fit models to the divergence estimates of each downsampled data set. The number of AA substitutions per year was calculated based on a linear regression using the median uncorrected hamming distance per year.

Divergent sites were counted between the sampling period consensus sequences for each subtype/CRF, excluding gaps. The number of mismatched sites between consensus sequences for sequential sampling periods was calculated for all sites. To calculate the number of mismatched sites inside CTL epitopes, CTL epitopes were predicted on each Env, Gag, and Pol subtype/CRF consensus sequence for the most frequent alleles (>5% frequency) in five major human subpopulations: African/African-American, Asian/Pacific Islands, European/European descent, Middle East/North coast of Africa, and South or Central America/Hispanic/Latinx (67). Peptides with a predicted binding score ranked in the top 99.5% compared with a set of random natural peptides and with a nanomolar affinity < 50 were considered potential CTL epitopes (i.e., strong binders). Rank scores and affinity were predicted with netMHCpan4.1 (68). The percentage of each consensus sequence covered by predicted CTL epitopes was calculated by counting the number of unique epitopes that occurred within each consensus sequence multiplied by nine (as each epitope was a 9-mer). Within each subtype/CRF, we counted the number of mismatched sites between consensus sequences for sequential sampling periods that fell within at least one CTL epitope in the consensus sequence. The percentage of mismatched sites falling within CTL epitopes was then compared with the percentage of protein sites covered by CTL epitopes. For Env, mismatches were listed for contact sites for the broadly neutralizing antibodies VRC01, CAP256-VRC26, PGT121-10-1074, 10E8, and 35O22. For Pol, specific attention was also given to sites corresponding to known drug resistance mutations associated with inhibitors for protease, nucleoside reverse transcriptase, non-nucleoside reverse transcriptase, and integrase as described by the Stanford University HIV Drug Resistance Database (https://hivdb.stanford.edu/cgi-bin/PositionPhenoSummary.cgi) (57).

## Data Availability

Sequences were downloaded from the HIV-1 LANL database. The code and alignments generated during this study are available at https://www.hivresearch.org/publication-supplements. The Hervé platform can be accessed at http://hiv1.shinyapps.io/Herve/.

## SUPPLEMENTAL MATERIAL

File S1 mbio.01749-23-s0001.txt Phylogenetic tree for Env sequences sampled between 1980-2020.

File S2 mbio.01749-23-s0002.txt Phylogenetic tree for Gag sequences sampled between 1980-2020.

File S3 mbio.01749-23-s0003.txt Phylogenetic tree for Pol sequences sampled between 1980-2020.

Supplemental information mbio.01749-23-s0004.pdf Figures S1 to S35, Tables S1 to S7, legends for Files S1 to S3, and Files S4 to S6.

ASM does not own the copyrights to Supplemental Material that may be linked to, or accessed through, an article. The authors have granted ASM a non-exclusive, world-wide license to publish the Supplemental Material files. Please contact the corresponding author directly for reuse.
