# Supplementary material for: HIV-1 Gag, Pol, and Env diversified with limited adaptation since the 1980s
Source: mBio. 2024 Feb 8;15(3):e01749-23. doi: 10.1128/mbio.01749-23 (PMC10936417; doi:10.1128/mbio.01749-23)
Supplement: Supplemental information — Figures S1 to S35, Tables S1 to S7, legends for Files S1 to S3, and Files S4 to S6. [file mbio.01749-23-s0004.pdf]

SI Appendix: HIV-1 proteins diversify with limited adaptation over decades

Eric Lewitus[a,b,1], Yifan Li[a,b,1], Hongjun Bai[a,b], Phuc Pham[a,b], Morgane Rolland[a,b]*

[a]U.S. Military HIV Research Program, Walter Reed Army Institute of Research, Silver Spring, MD

[b]Henry M. Jackson Foundation for the Advancement of Military Medicine, Inc., Bethesda, MD

[1]Equal contribution

*Corresponding author: Morgane Rolland

Email: mrolland@hivresearch.org

This PDF file includes:

Figures S1 to S35

Tables S1 to S7

Files S1 to S6

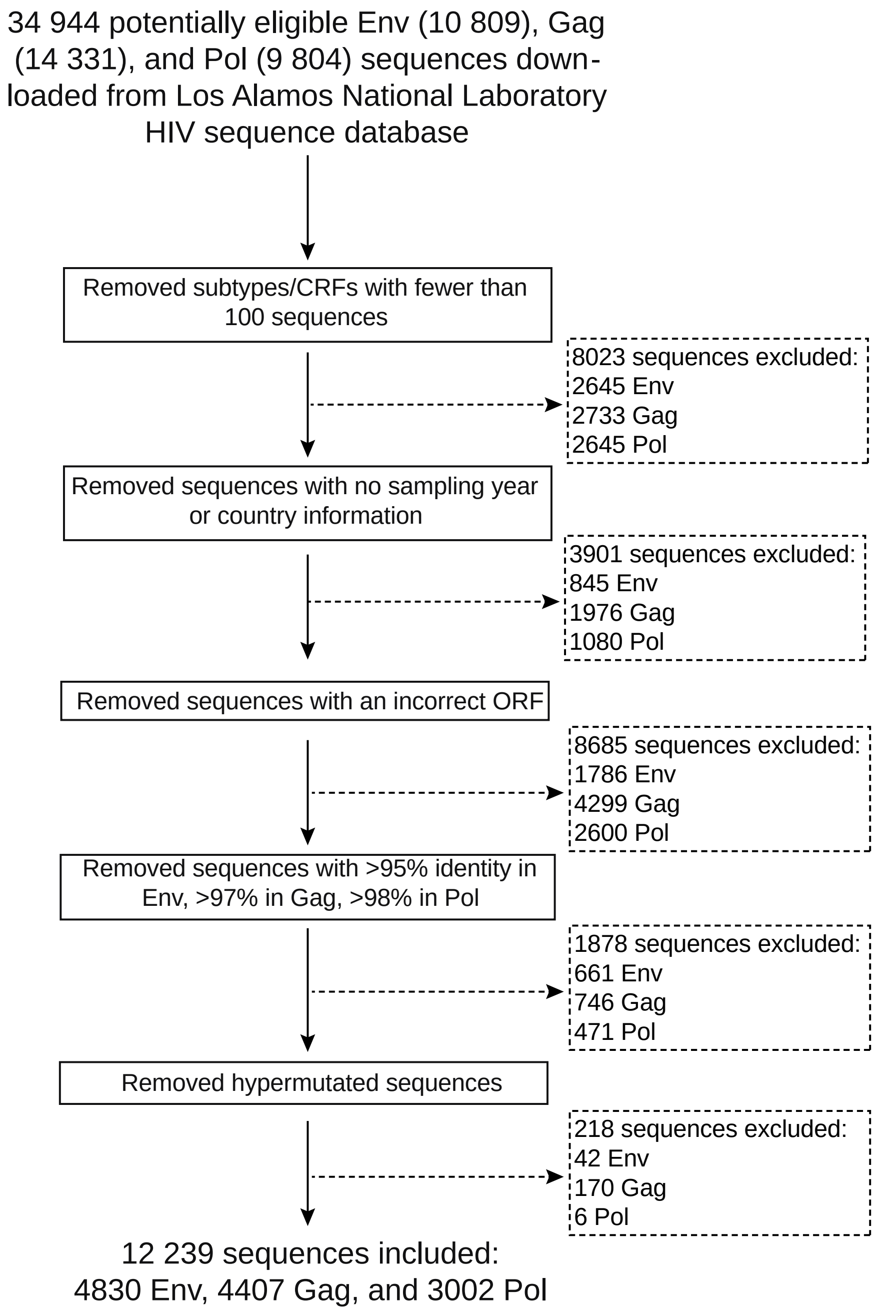

Fig. S1. Curation protocol for filtering Env, Gag, and Pol amino acid sequences.

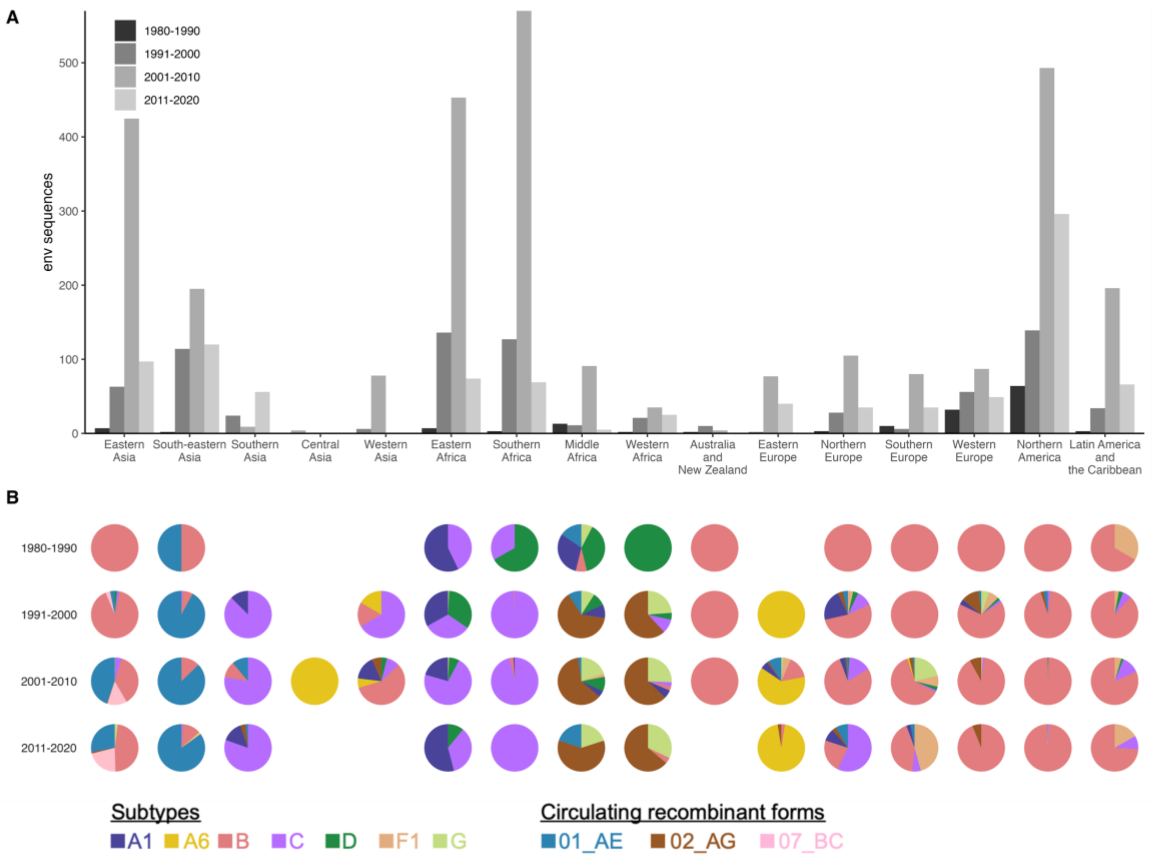

Fig. S2. Regional and temporal distribution of Env HIV-1 subtypes and CRFs. (A) The number of Env sequences sampled in different geographic regions by decade. (B) The proportion of seven subtypes and three CRFs in each geographic region by decade.

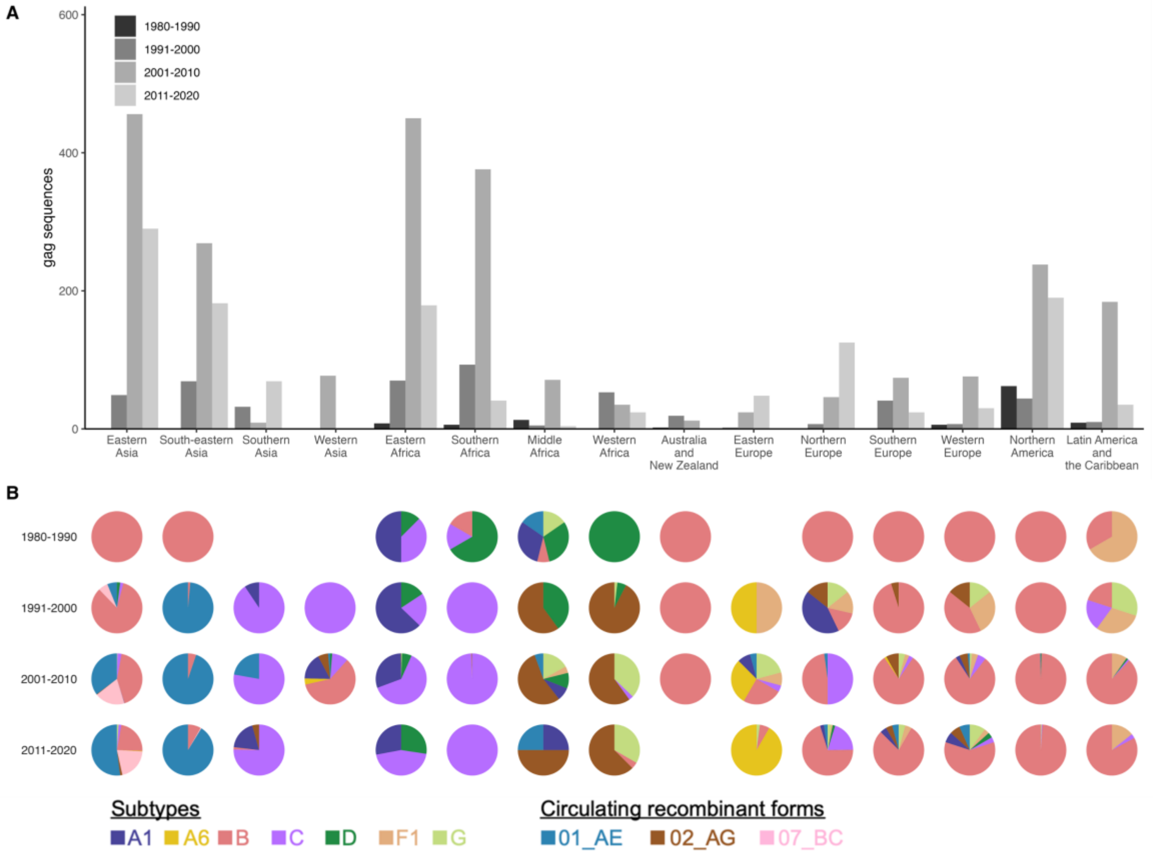

Fig. S3. Regional and temporal distribution of Gag HIV-1 subtypes and CRFs.(A) The number of Gag sequences sampled in different geographic regions by decade. (B) The proportion of seven subtypes and three CRFs in each geographic region by decade.

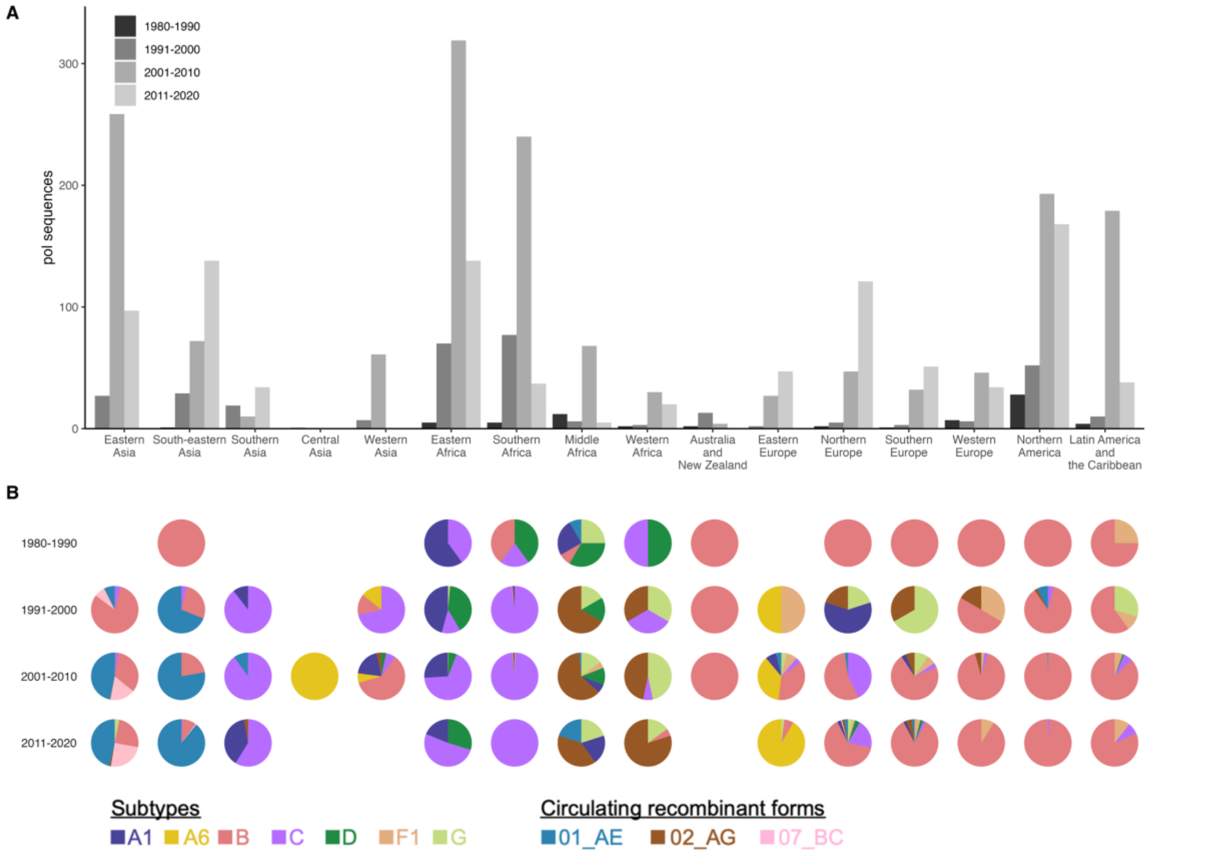

Fig. S4. Regional and temporal distribution of Pol HIV-1 subtypes and CRFs.(A) The number of Pol sequences sampled in different geographic regions by decade. (B) The proportion of seven subtypes and three CRFs in each geographic region by decade.

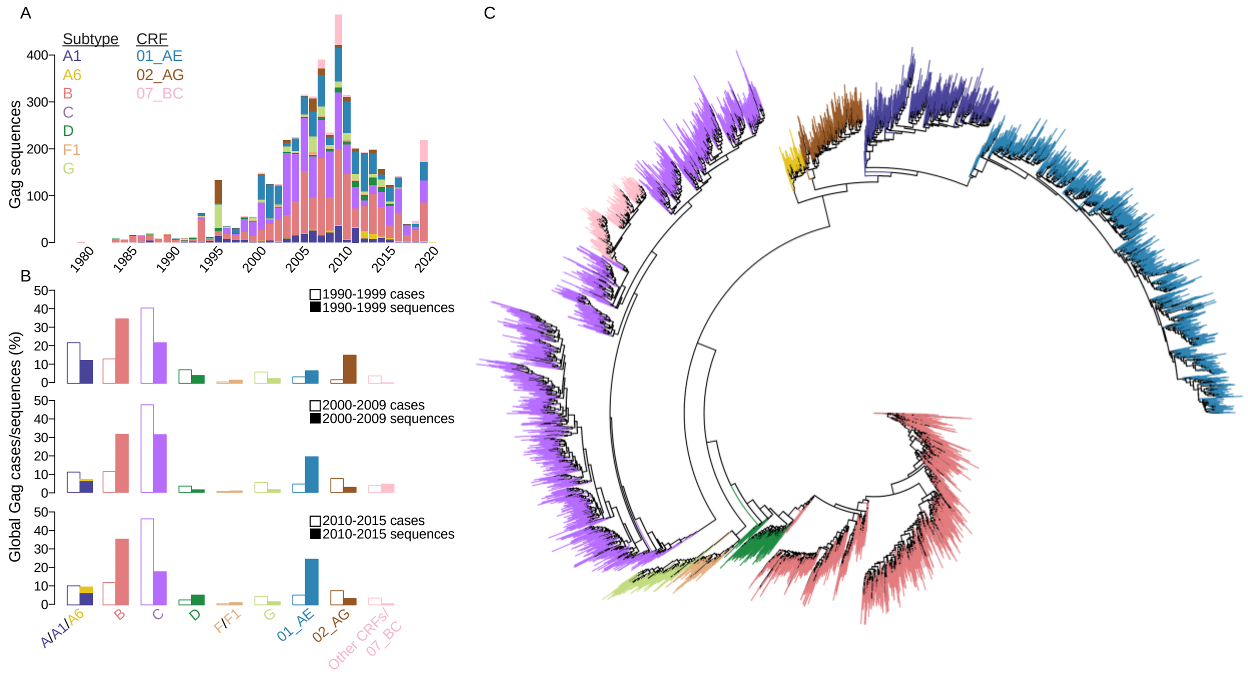

Fig. S5. HIV-1 Gag subtype and CRF sequences over time. (A) The number of Gag sequences in our dataset sampled between 1980-2020 by subtype/CRF. (B) The number of PLWH with a given subtype/CRF (as a percentage of all PLWH) and number of Gag sequences of that subtype/CRF (as a percentage of Gag sequences of all subtypes/CRFs in the dataset) in our dataset between 1990-1999, 2000-2009, and 2010-2015. (C) The reconstructed Gag phylogeny with tips colored by subtype and CRF.

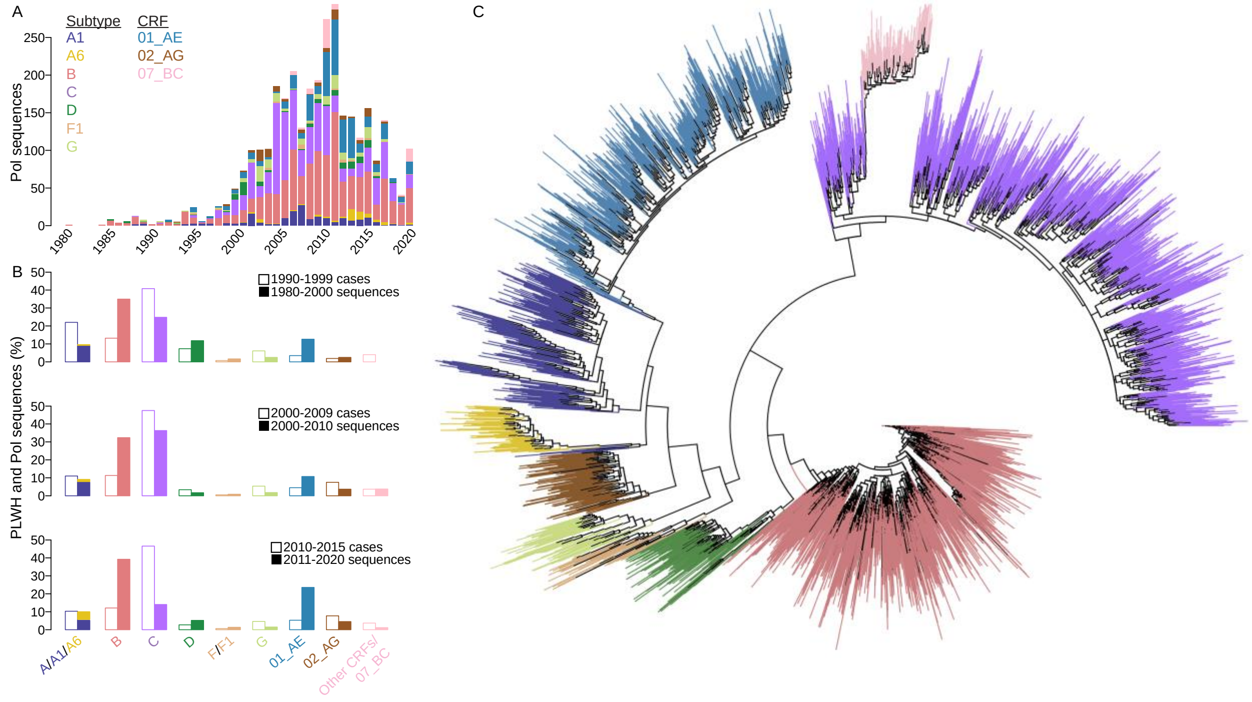

Fig. S6. HIV-1 Pol subtype and CRF sequences over time. (A) The number of Pol sequences in our dataset sampled between 1980-2020 by subtype/CRF. (B) The number of PLWH with a given subtype/CRF (as a percentage of all PLWH) and number of Pol sequences of that subtype/CRF (as a percentage of Pol sequences of all subtypes/CRFs in the dataset) in our dataset between 1990-1999, 2000-2009, and 2010-2015. (C) The reconstructed Pol phylogeny with tips colored by subtype and CRF.

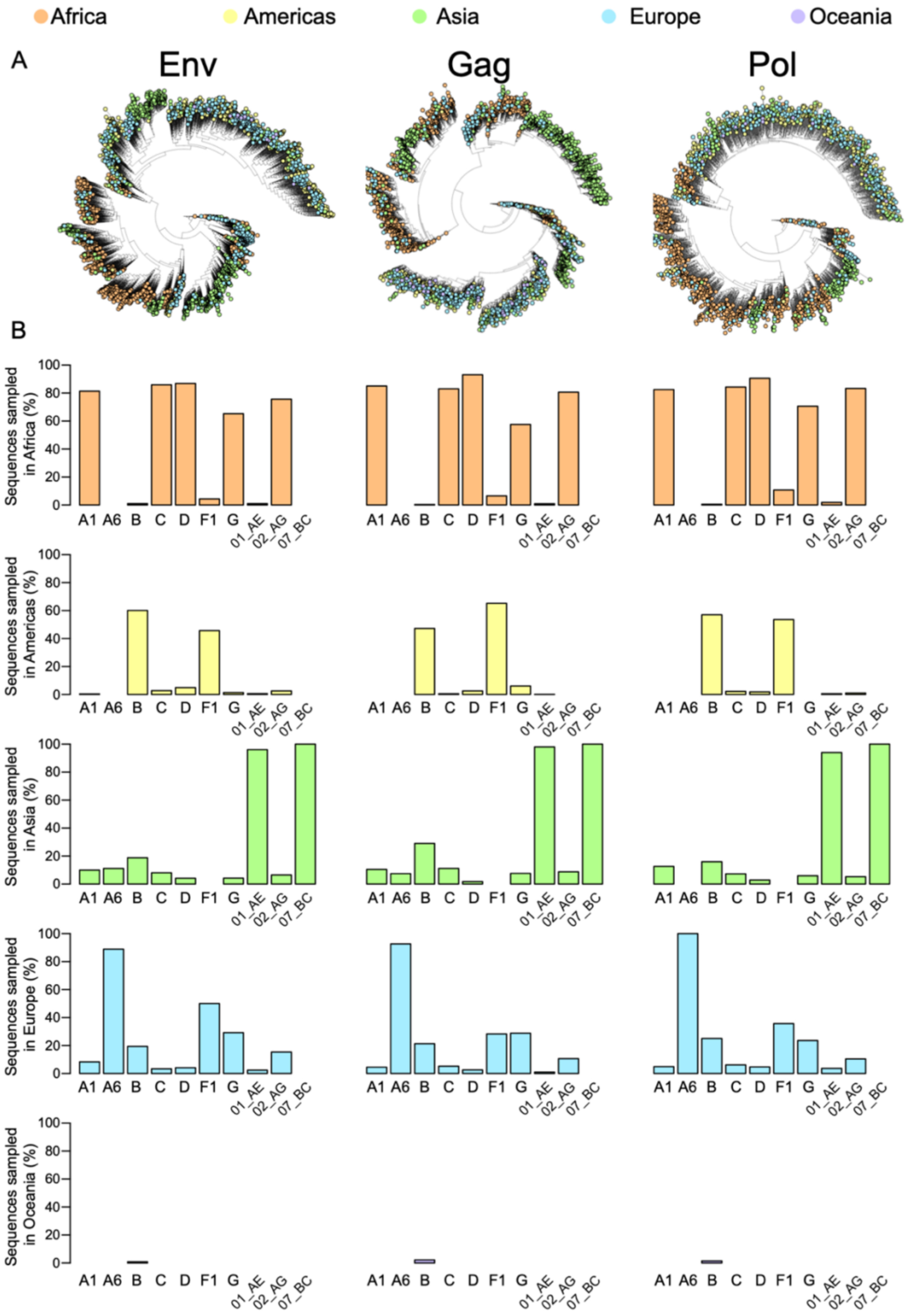

Fig. S7. Subtype/CRF distribution across continents. (A) Reconstructed phylogenies for Env, Gag, and Pol sequences with tips colored by the continent each sequence was sampled from. (B) Barplots of the percentage of Env, Gag, and Pol sequences sampled in Africa, the Americas, Asia, Europe, and Oceania.

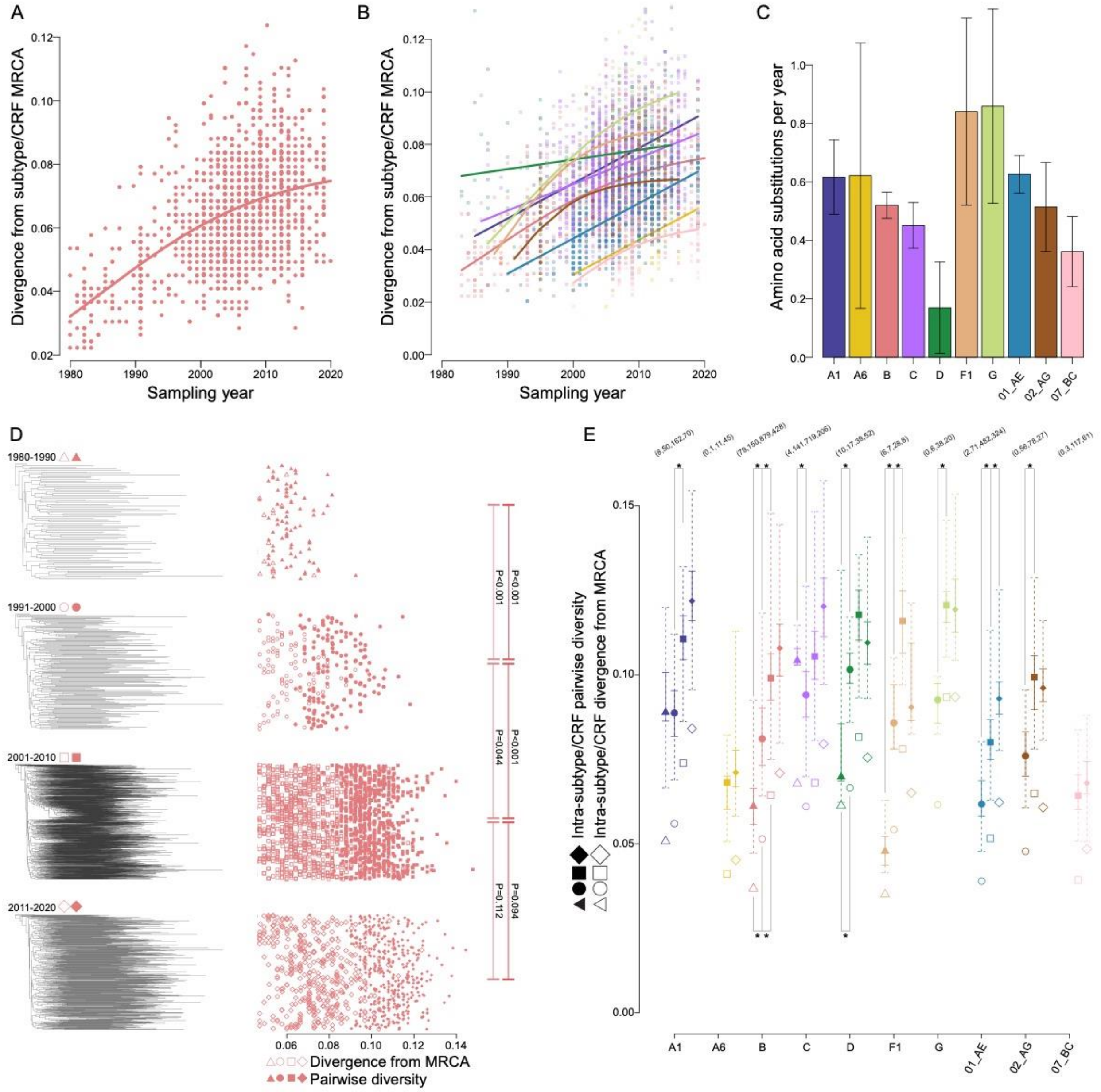

Fig. S8. Subtype/CRF divergence and diversity in HIV-1 Gag. (A) Divergence of each subtype B Gag sequence from the subtype B MRCA by sampling year. A best fit curve is shown. (B) Best fit curves for each subtype/CRF for divergence from each subtype/CRF MRCA by sampling year. (C) Amino acid substitutions per year for each subtype/CRF estimated from a linear regression of the median uncorrected hamming distance per year. (D) Reconstructed phylogenies for subtype B Gag sequences sampled during each sampling period; the divergence from the MRCA (open shapes) and median pairwise diversity (filled shapes) for each sequence corresponding to a tip in the phylogeny. (E) Median divergence from the MRCA (open shapes) and median pairwise diversity (filled shapes) for each subtype/CRF at each sampling period. Solid bars indicate IQR1 and IQR3 and dashed bars indicate minimum and maximum values of pairwise diversity. Pairwise significant differences between sampling periods of divergence from the MRCA (below) and pairwise diversity (above) and the number of sequences corresponding to each subtype/CRF for sampling periods are shown.

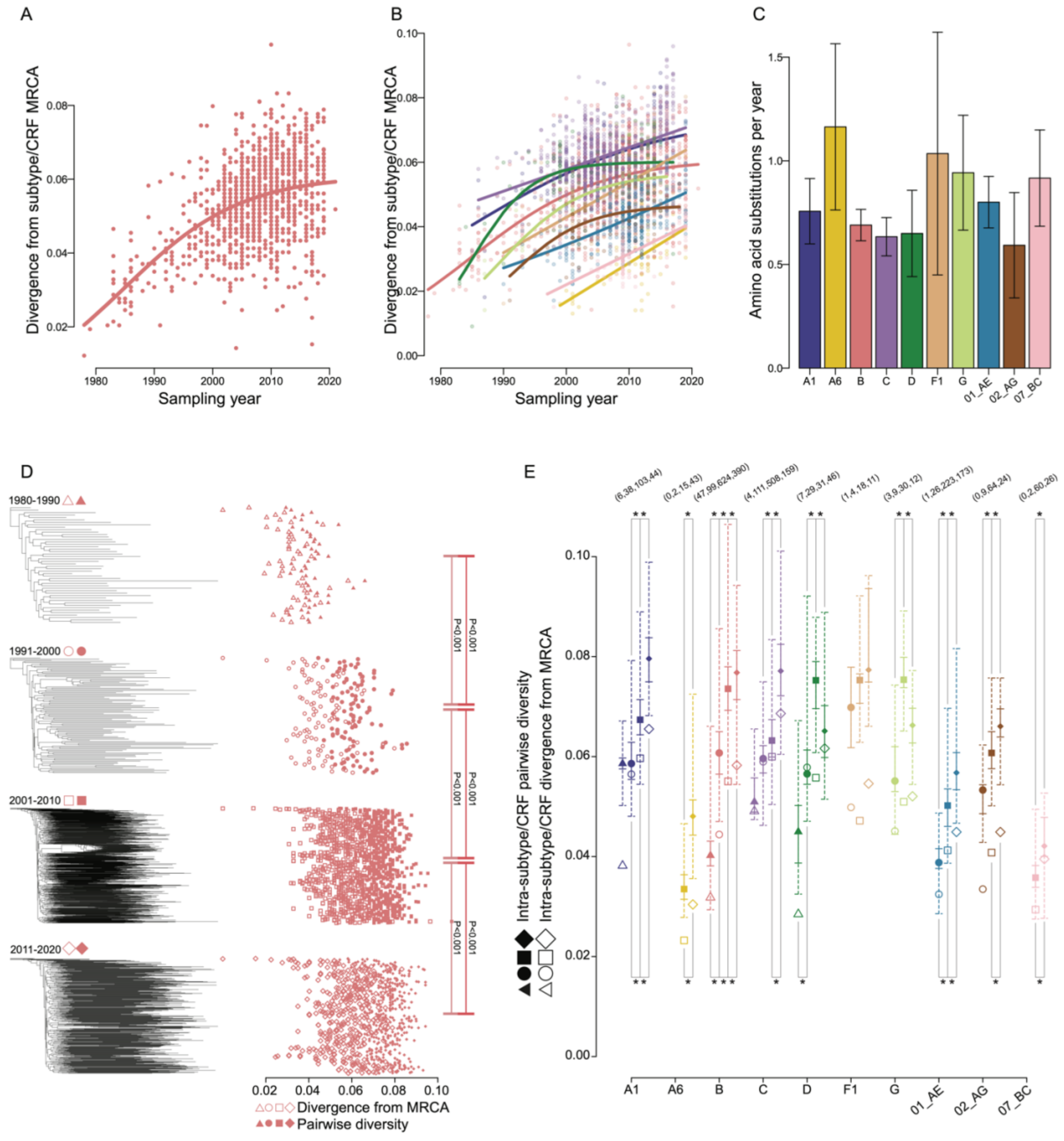

Fig. S9. Subtype/CRF divergence and diversity in HIV-1 Pol (A) Divergence of each subtype B Pol sequence from the subtype B MRCA by sampling year. A best fit curve is shown. (B) Best fit curves for each subtype/CRF for divergence from each subtype/CRF MRCA by sampling year. (C) Amino acid substitutions per year for each subtype/CRF estimated from a linear regression of the median uncorrected hamming distance per year. (D) Reconstructed phylogenies for subtype B Pol sequences sampled during each sampling period; the divergence from the MRCA (open shapes) and median pairwise diversity (filled shapes) for each sequence corresponding to a tip in the phylogeny. (E) Median divergence from the MRCA (open shapes) and median pairwise diversity (filled shapes) for each subtype/CRF at each sampling period. Solid bars indicate IQR1 and IQR3 and dashed bars indicate minimum and maximum values of pairwise diversity. Pairwise significant differences between sampling periods of divergence from the MRCA (below) and pairwise diversity (above) and the number of sequences corresponding to each subtype/CRF for sampling periods are shown.

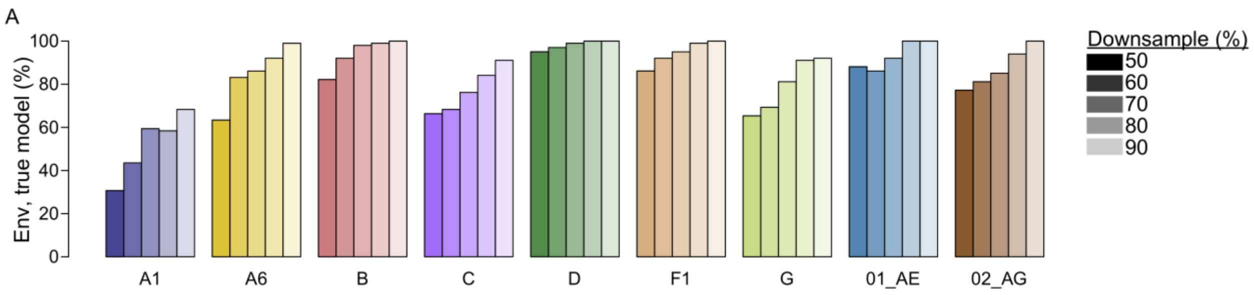

Fig. S10. Growth model recovery estimates for downsampled Env alignments. The frequency of true model recovery (based on the full alignment) for 100 downsampled Env subtype/CRF alignments at 50-90%. Across subtypes/CRFs, downsampled alignments at 50%,60%,70%,80%, and 90% returned the true model at a median of 77.2%, 83.2%, 86.1%, 94.1%, and 100%.

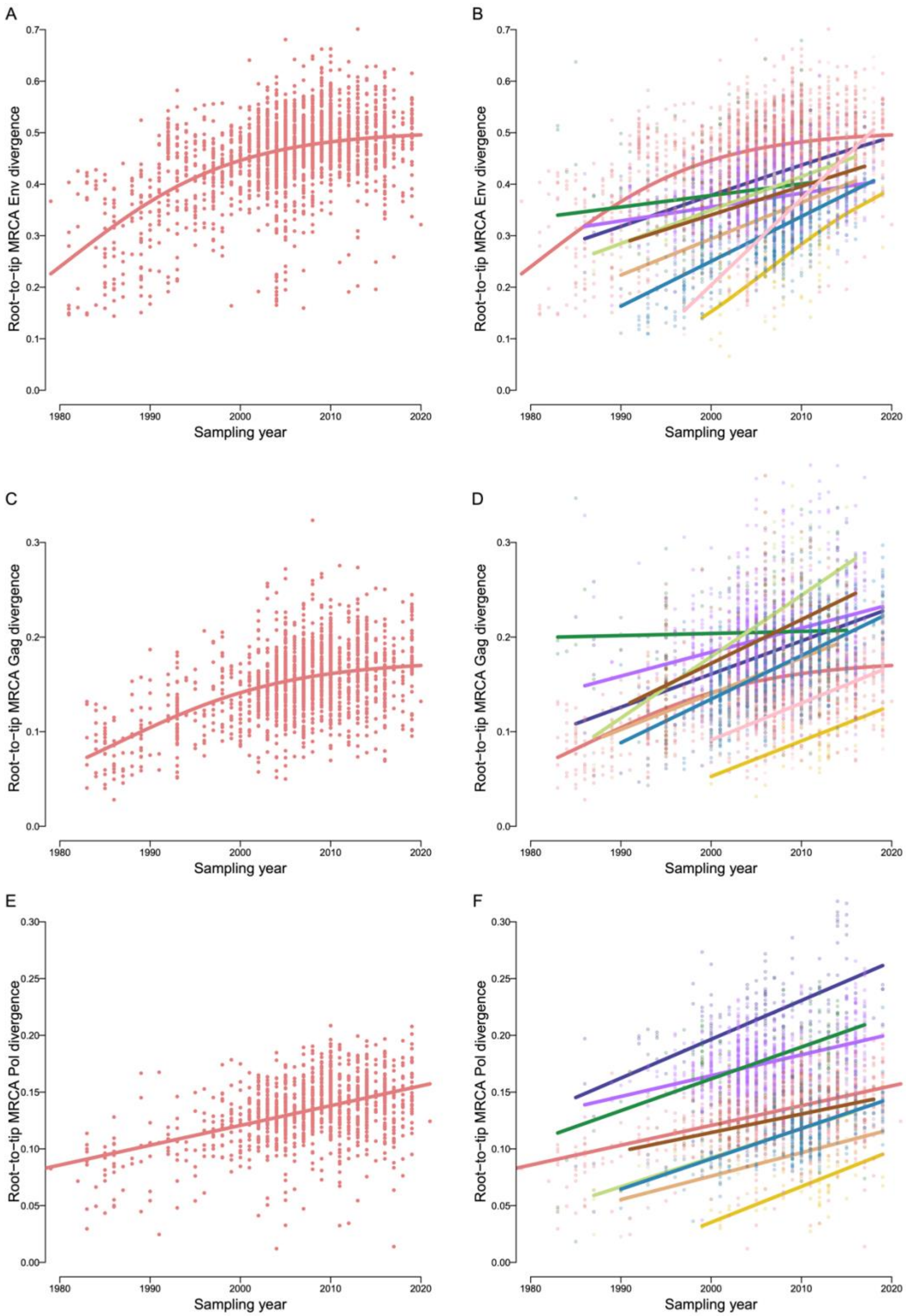

Fig. S11. Subtype/CRF divergence in HIV-1 Env, Gag, and Pol. Root-to-tip phylogenetic divergence of each subtype B (A) Env, (C) Gag, and (E) Pol sequence from the subtype B MRCA by sampling year. Best fit curves are shown. Best fit curves for each subtype/CRF for root-to-tip phylogenetic divergence from each (B) Env, (D) Gag, and (F) Pol subtype/CRF MRCA by sampling year.

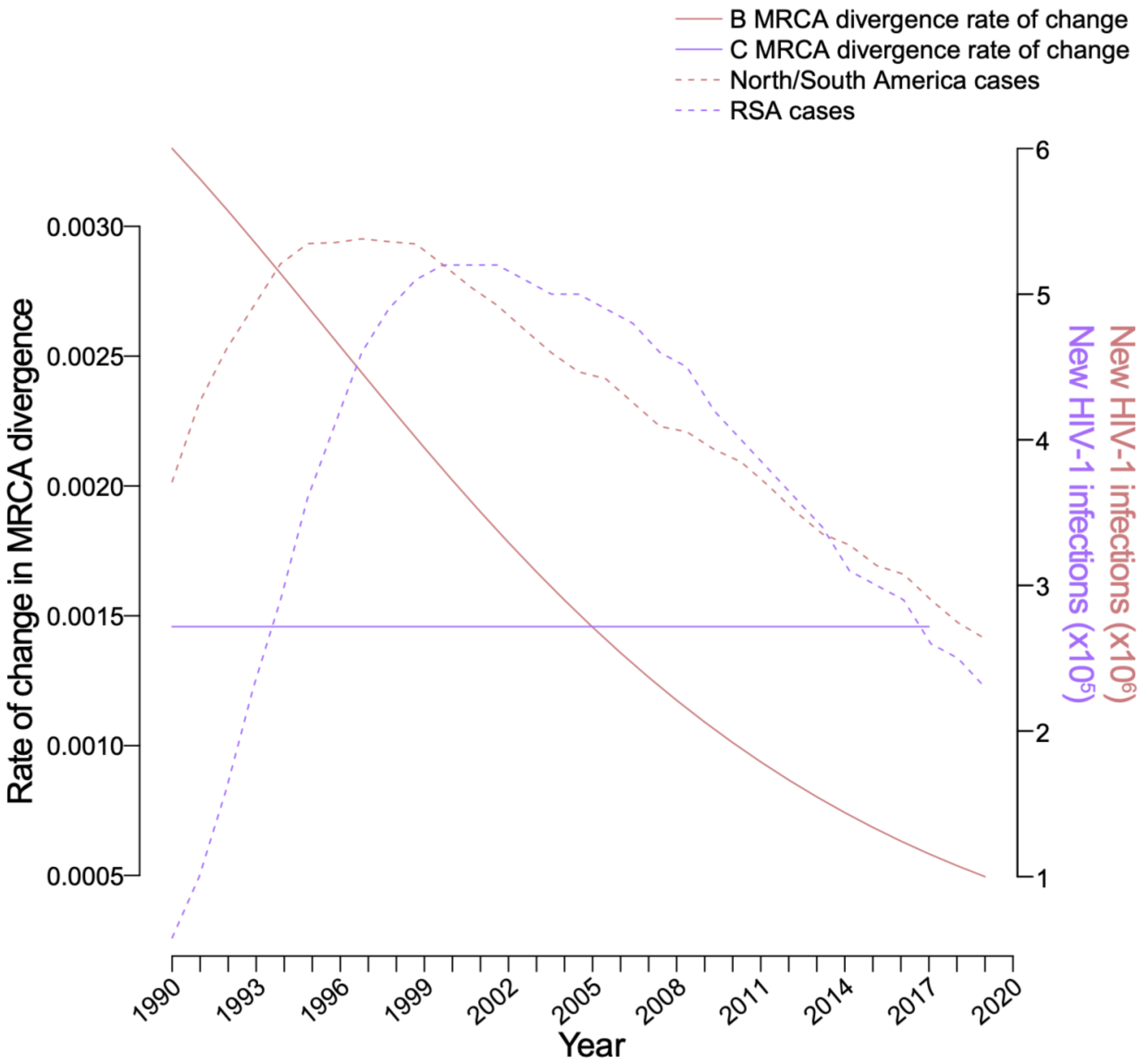

Fig. S12. New HIV-1 infections per year and subtype B and C MRCA divergence. The rate of change of MRCA divergence of subtype B (solid red line) and C (solid purple line) Env sequences over time. The number of new HIV-1 infections per year in the Americas (dashed red line) and the Republic of South Africa (dashed purple line).

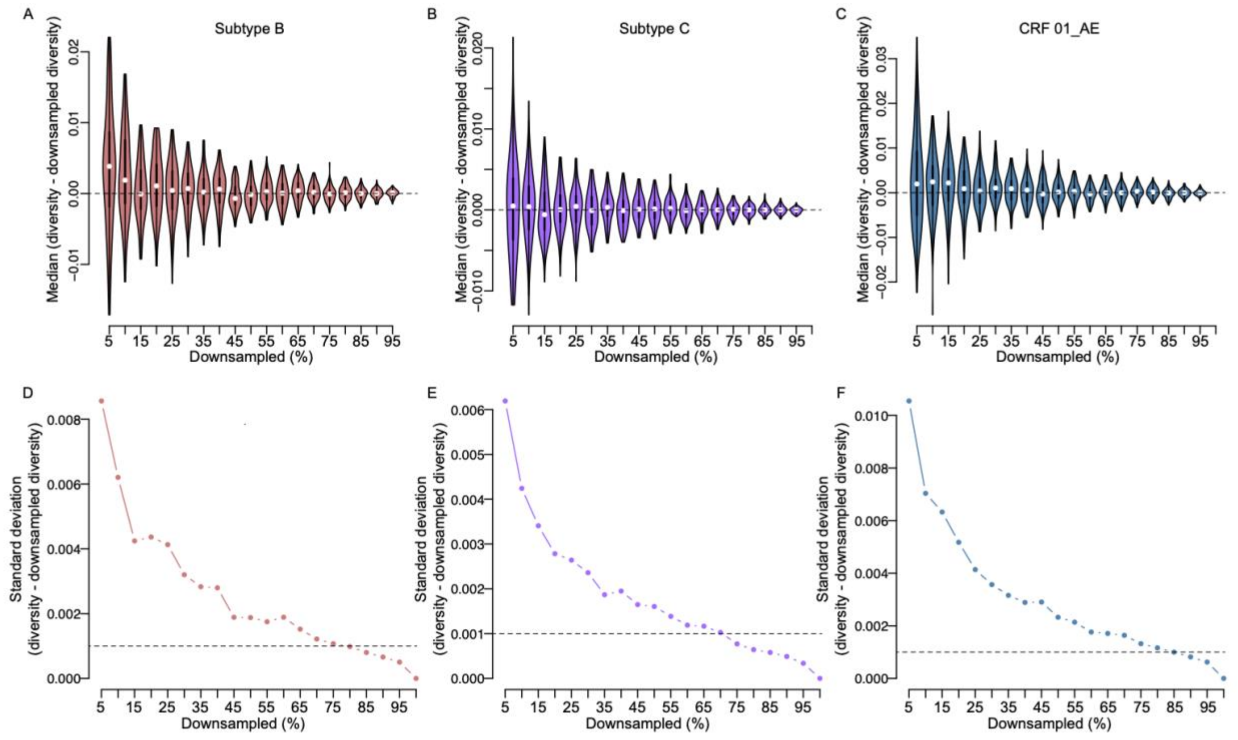

Fig. S13. Differences in pairwise diversity estimates in downsampled subtype B, C and CRF 01_AE alignments. Mean differences and standard deviations in diversity estimates between the full (A,D) subtype B, (B,E) subtype C, and (C,F) CRF 01_AE alignments and 100 downsampled alignments. The percentage of downsampled sequences are indicated on the x-axis.

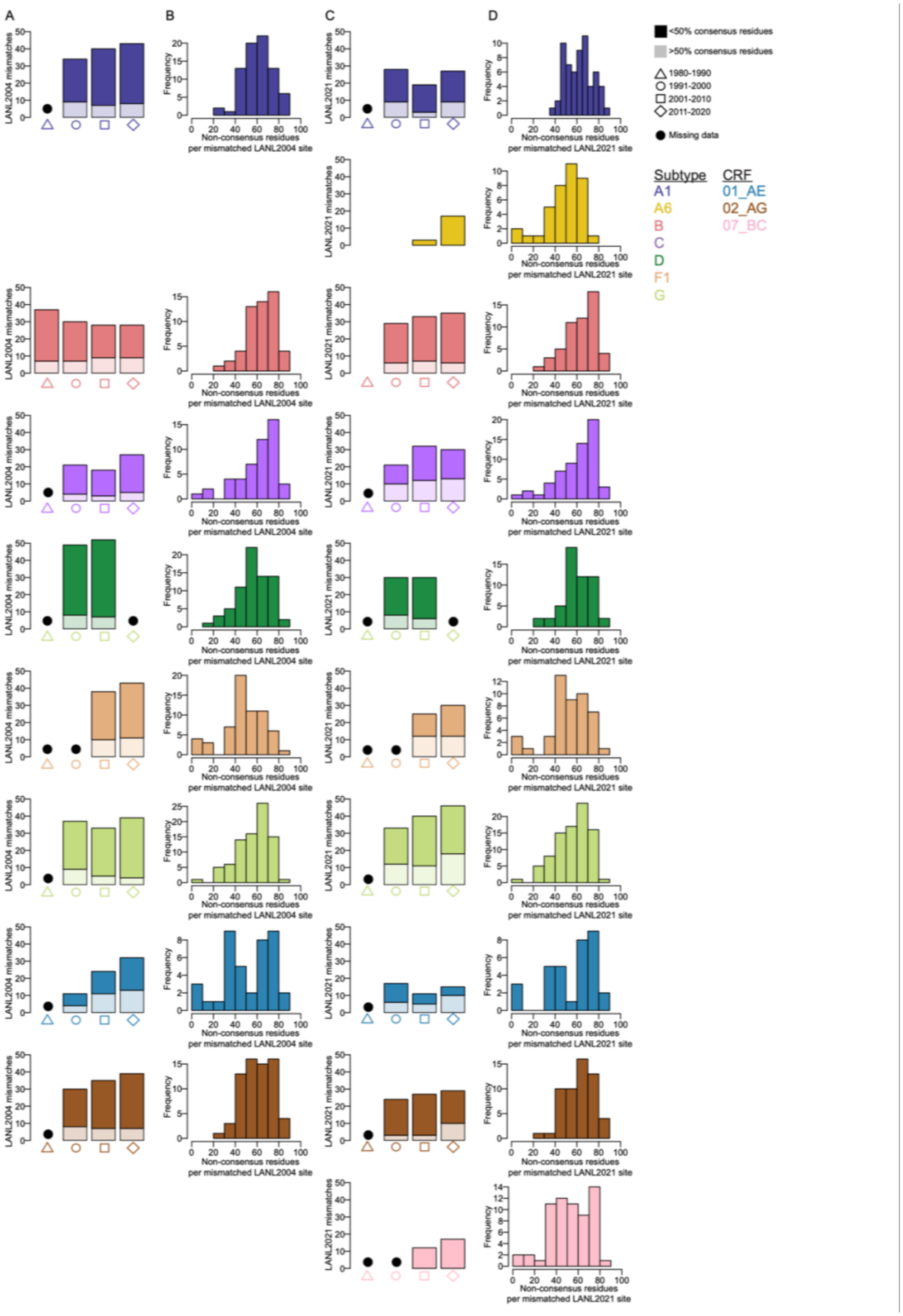

Fig. S14. Mismatched residues between LANL and our consensuses for Env. (A,C) Barplots of the number of mismatches amino acid sites between subtype/CRF LANL consensuses generated with sequences sampled up to 2004 (A) and up to 2021 (C) with the consensus we generated for sequences sampled between 1980-1990, 1991-2000, 2001-2010, and 2011-2020. (B,D) The frequency of non-consensus residues across all sequences in a subtype/CRF alignment at sites mismatched with the (B) LANL2004 and (D) LANL2021 consensuses.

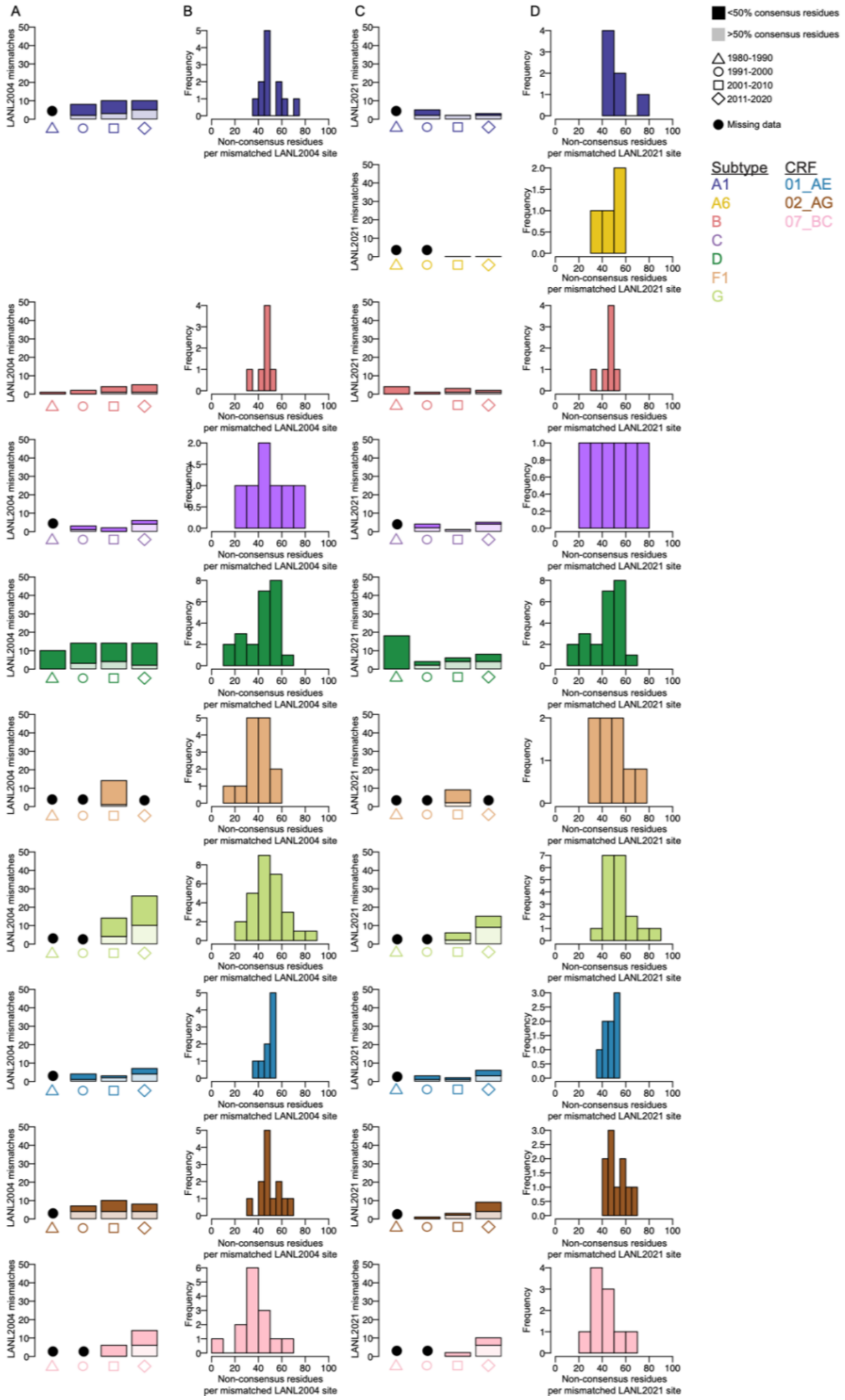

Fig. S15. Mismatched residues between LANL and our consensuses for Gag (A,C) Barplots of the number of mismatches amino acid sites between subtype/CRF LANL consensuses generated with sequences sampled up to 2004 (A) and up to 2021 (C) with the consensus we generated for sequences sampled between 1980-1990, 1991-2000, 2001-2010, and 2011-2020. (B,D) The frequency of non-consensus residues across all sequences in a subtype/CRF alignment at sites mismatched with the (B) LANL2004 and (D) LANL2021 consensuses.

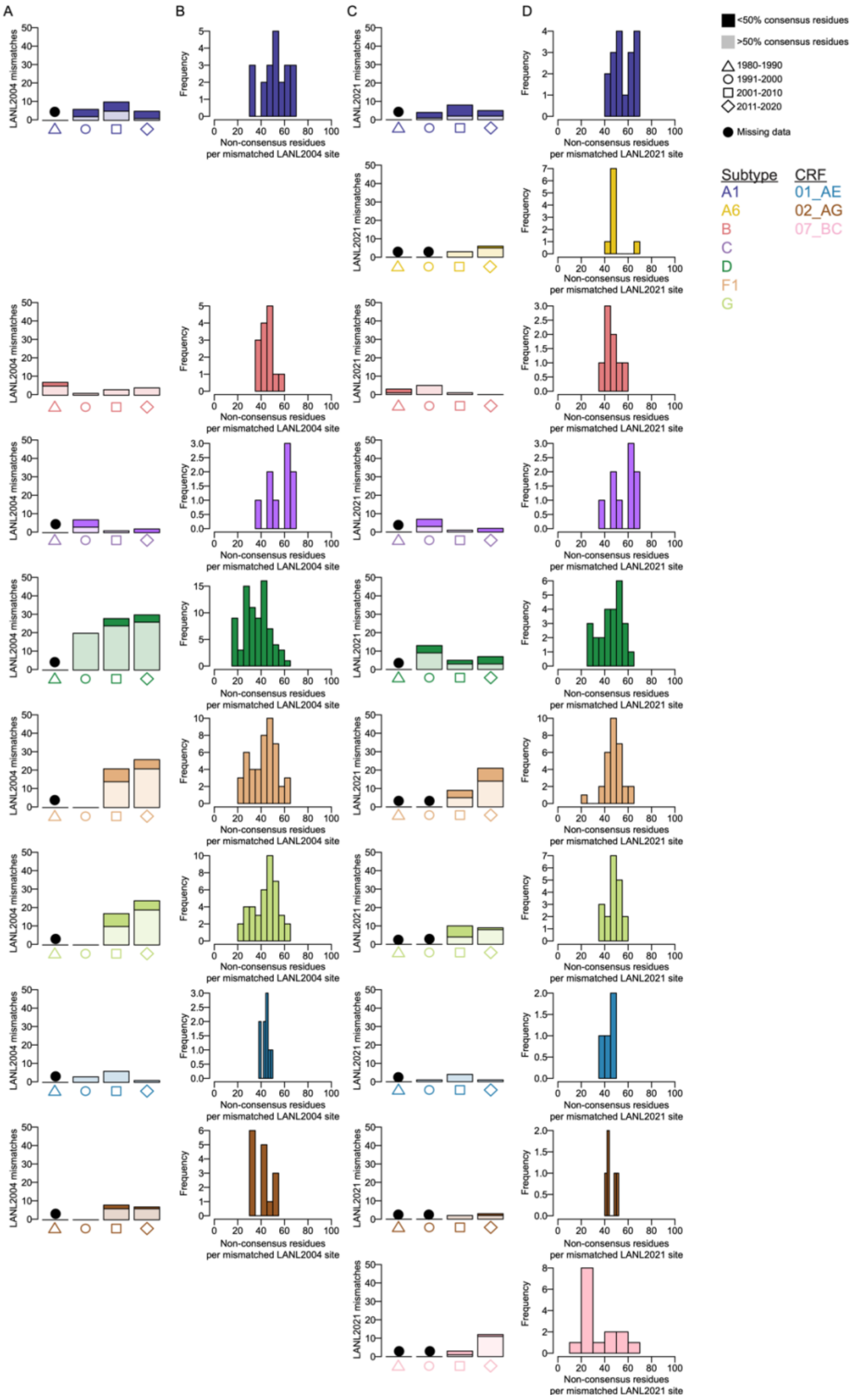

Fig. S16. Mismatched residues between LANL and our consensuses for Pol (A,C) Barplots of the number of mismatched amino acid sites between subtype/CRF LANL consensuses generated with sequences sampled up to 2004 (A) and up to 2021 (C) with the consensus we generated for sequences sampled between 1980-1990, 1991-2000, 2001-2010, and 2011-2020. (B,D) The frequency of non-consensus residues at sites mismatched with the (B) LANL2004 and (D) LANL2021 consensuses.

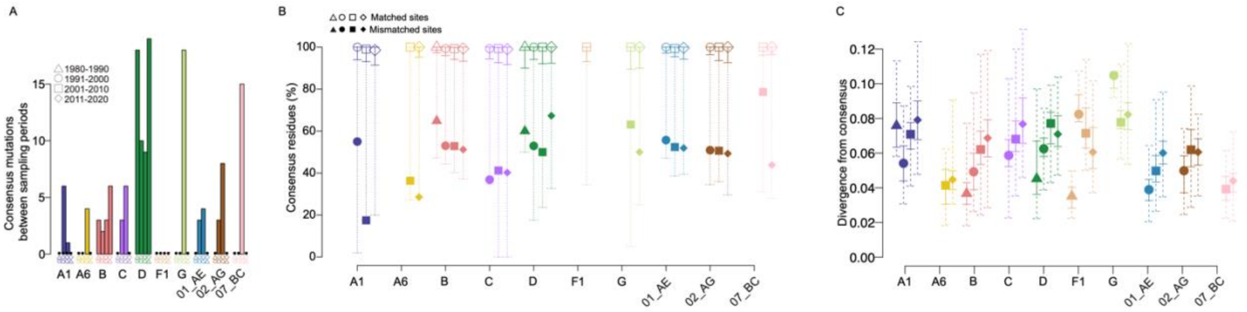

Fig. S17. Subtype/CRF mutations over time in HIV-1 Gag. (A) Barplots of the number of sites with mismatched residues between sampling periods for each Gag subtype/CRF; shapes in each row below bars indicate the two sampling periods compared. Black dots are shown when the consensus was not calculated due to too few sequences. (B) The percentage of consensus residues, as measured from the sampling period consensus, at all sites matched (open shapes) or mismatched (filled shapes) between consensus sequences. Open shapes indicate median values, solid bars indicate IQR1 and IQR3 for matched sites, dashed bars indicate minimum and maximum values for matched sites; closed shapes indicate median values for mismatched sites. (C) Divergence from the consensus at each sampling period; solid bars indicate IQR1 and IQR3, dashed bars indicate minimum and maximum values.

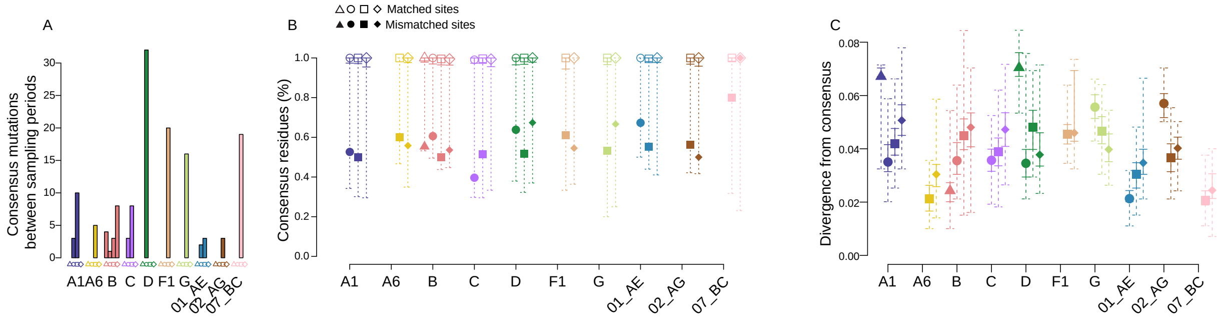

Fig. S18. Subtype/CRF mutations over time in HIV-1 Pol (A) Barplots of the number of sites with mismatched residues between sampling periods for each Pol subtype/CRF; shapes in each row below bars indicate the two sampling periods compared. Black dots are shown when the consensus was not calculated due to too few sequences. (B) The percentage of consensus residues, as measured from the sampling period consensus, at all sites matched (open shapes) or mismatched (filled shapes) between consensus sequences. Open shapes indicate median values, solid bars indicate IQR1 and IQR3 for matched sites, dashed bars indicate minimum and maximum values for matched sites; closed shapes indicate median values for mismatched sites. (C) Divergence from the consensus at each sampling period; solid bars indicate IQR1 and IQR3, dashed bars indicate minimum and maximum values.

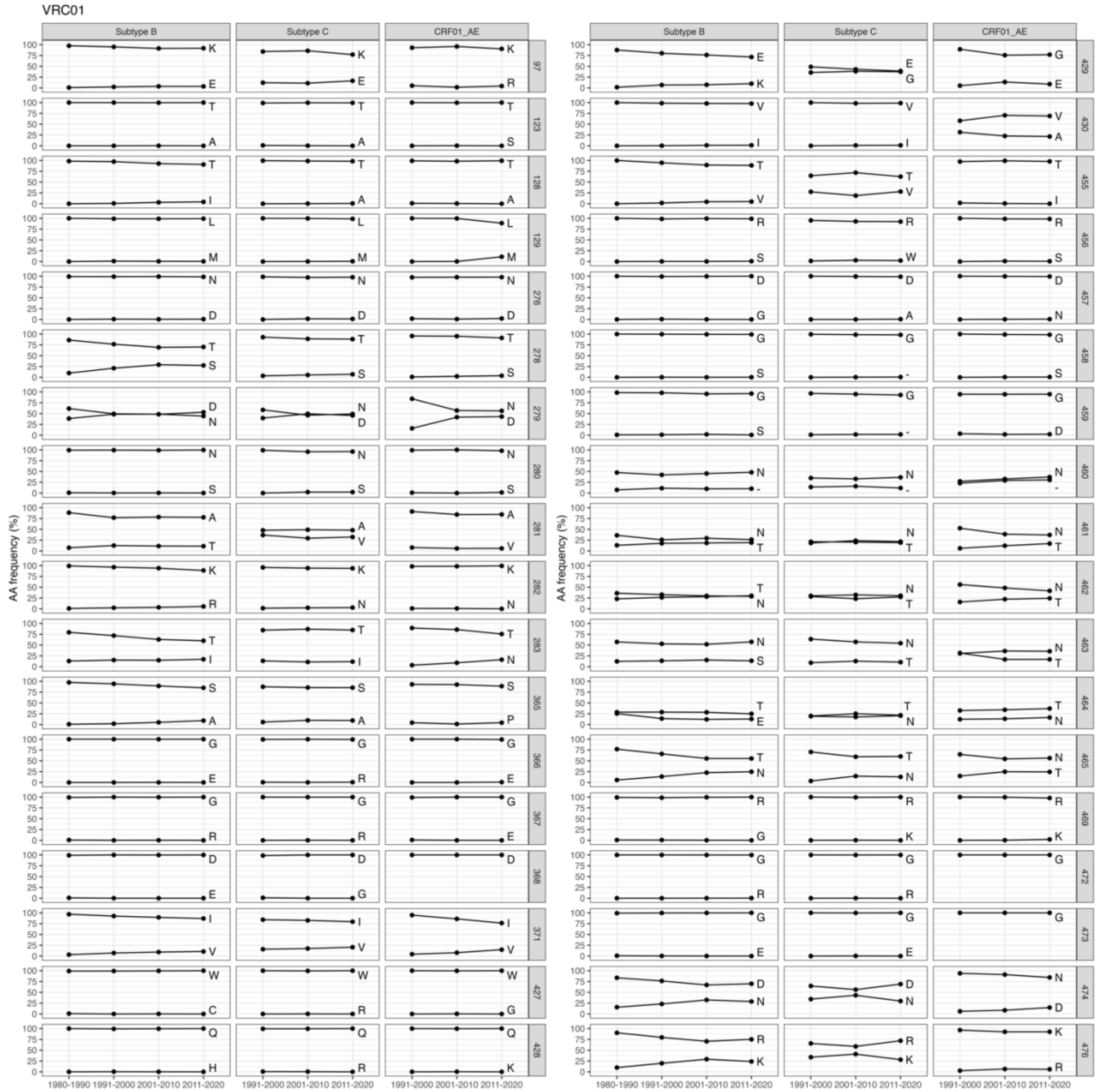

Fig. S19. Dominant residues at VRC01 Ab epitope sites in subtypes B and C and CRF01_AE. The frequency of the two most common residues over time at VRC01 Ab epitope sites in alignments of subtypes B and C and CRF01_AE. Numbers on the right-hand side refer to HXB2 AA sites.

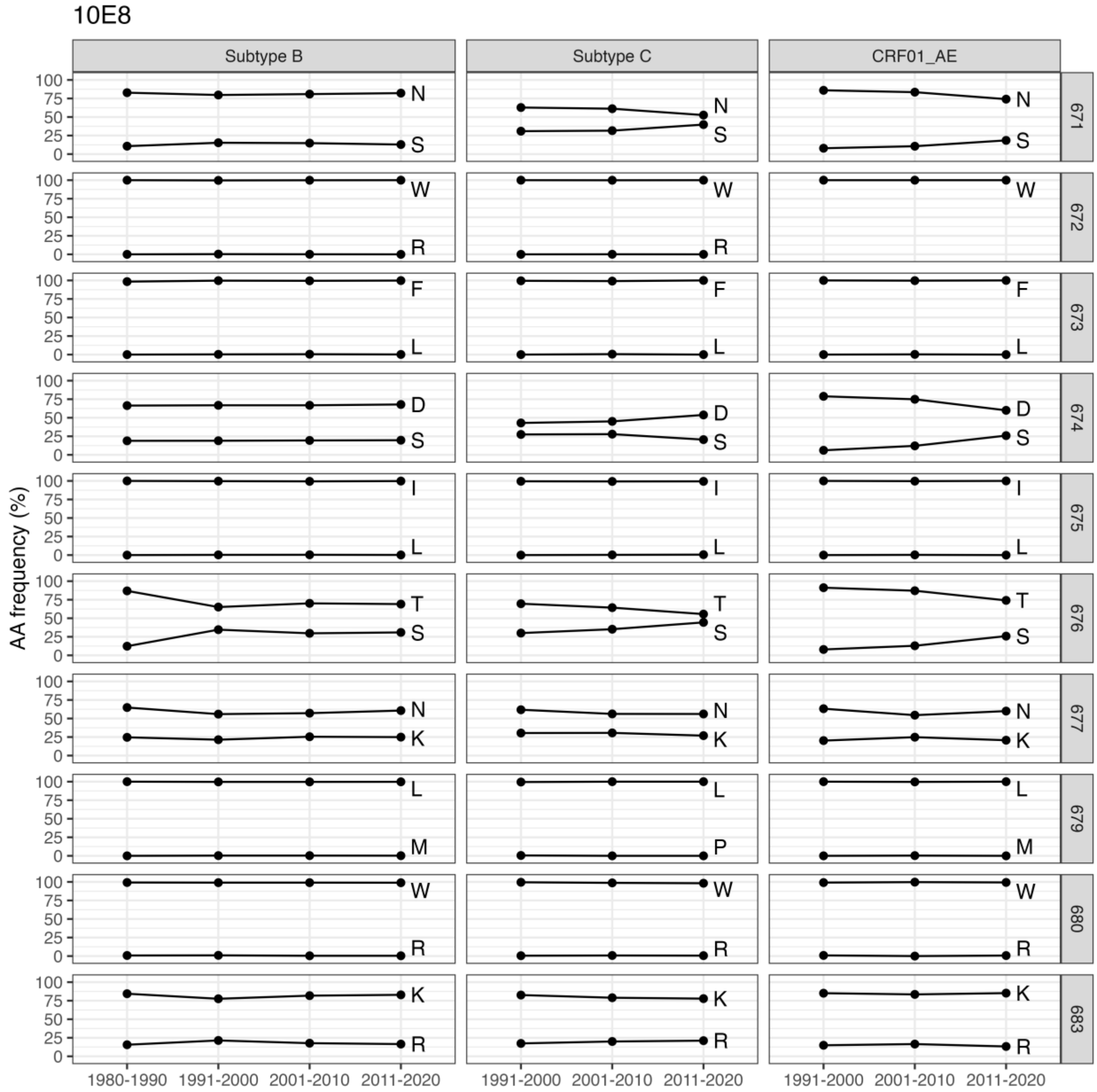

Fig. S20. Dominant residues at 10E8 Ab epitope sites in subtypes B and C and CRF01_AE. The frequency of the two most common residues over time at 10E8 Ab epitope sites in alignments of subtypes B and C and CRF01_AE. Numbers on the right-hand side refer to HXB2 AA sites.

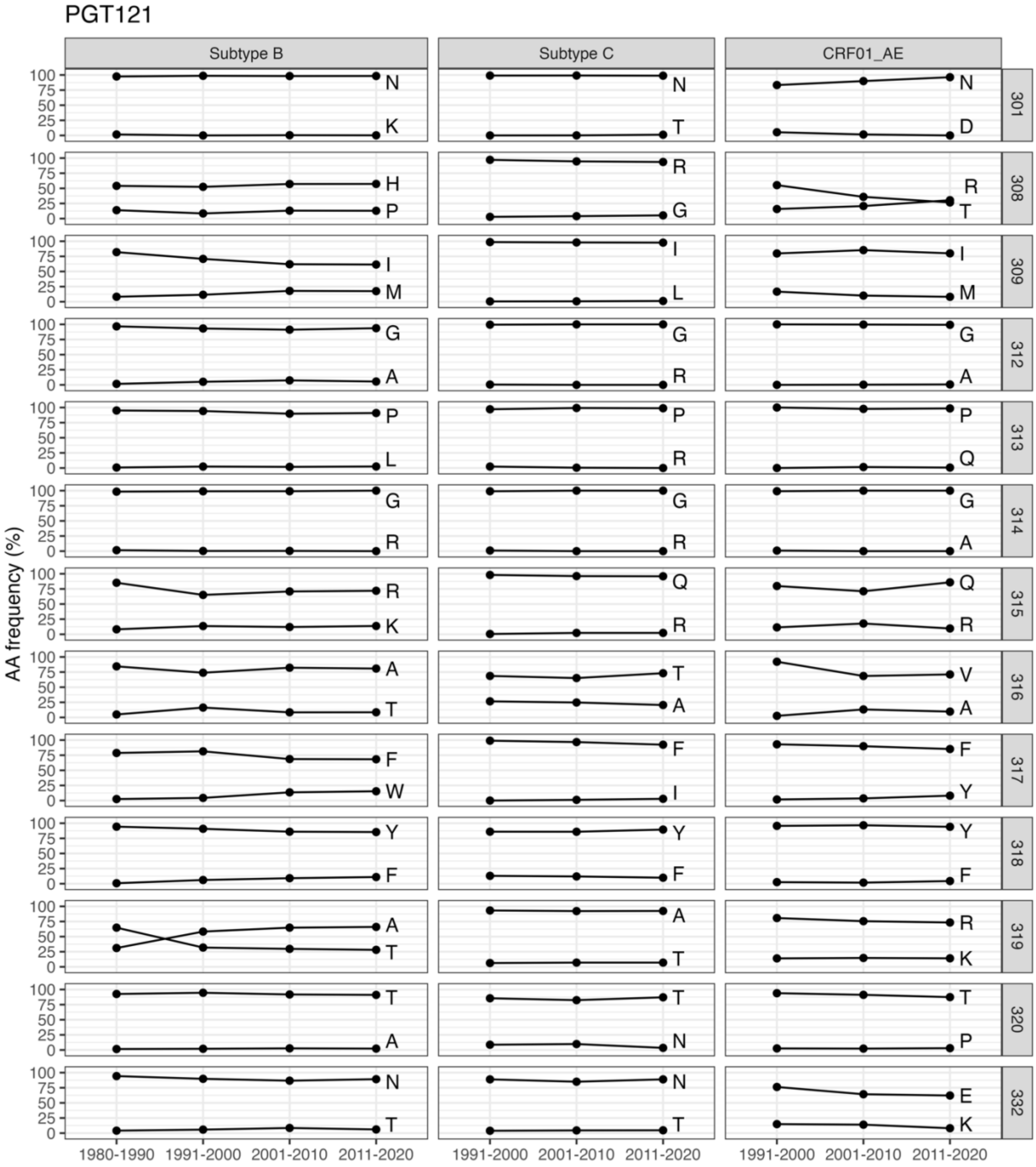

Fig. S21. Dominant residues at PGT121 Ab epitope sites in subtypes B and C and CRF01_AE. The frequency of the two most common residues over time at PGT121 Ab epitope sites in alignments of subtypes B and C and CRF01_AE. Numbers on the right-hand side refer to HXB2 AA sites.

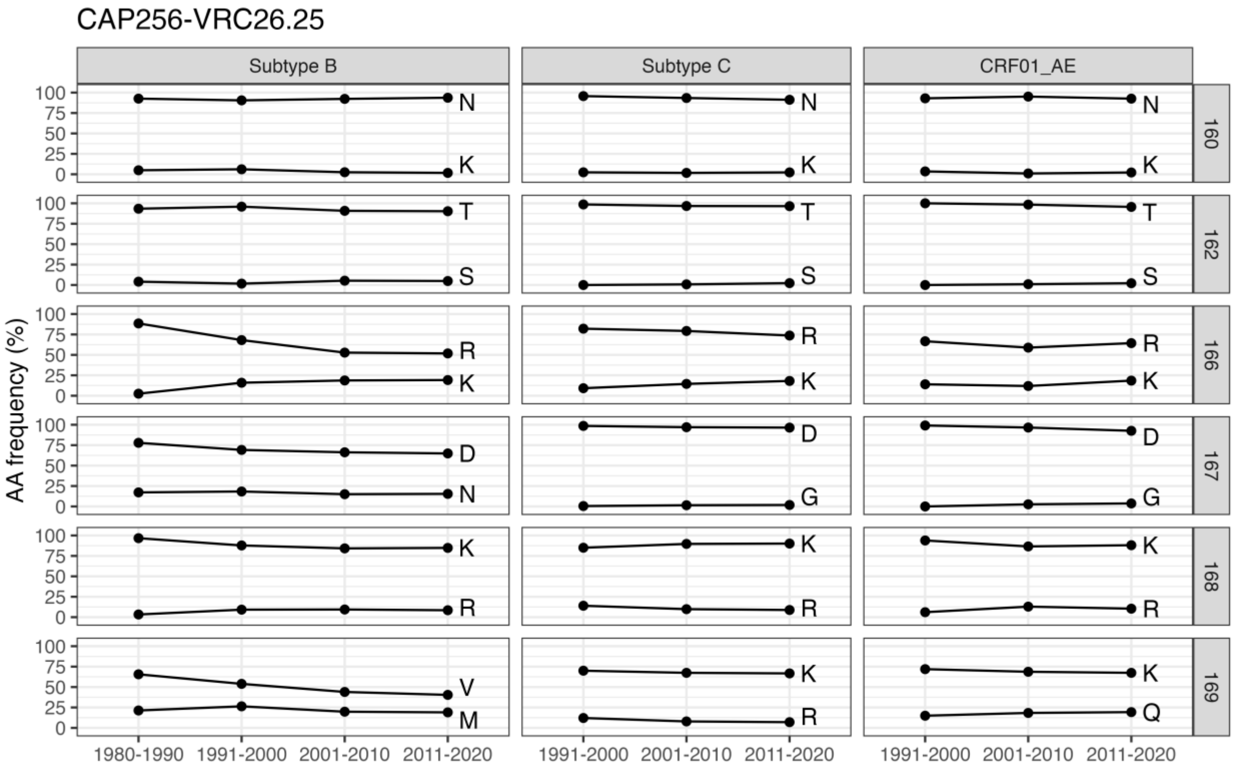

Fig. S22. Dominant residues at CAP256-VRC26.25 Ab epitope sites in subtypes B and C and CRF01_AE. The frequency of the two most common residues over time at CAP256-VRC26.25 Ab epitope sites in alignments of subtypes B and C and CRF01_AE. Numbers on the right-hand side refer to HXB2 AA sites.

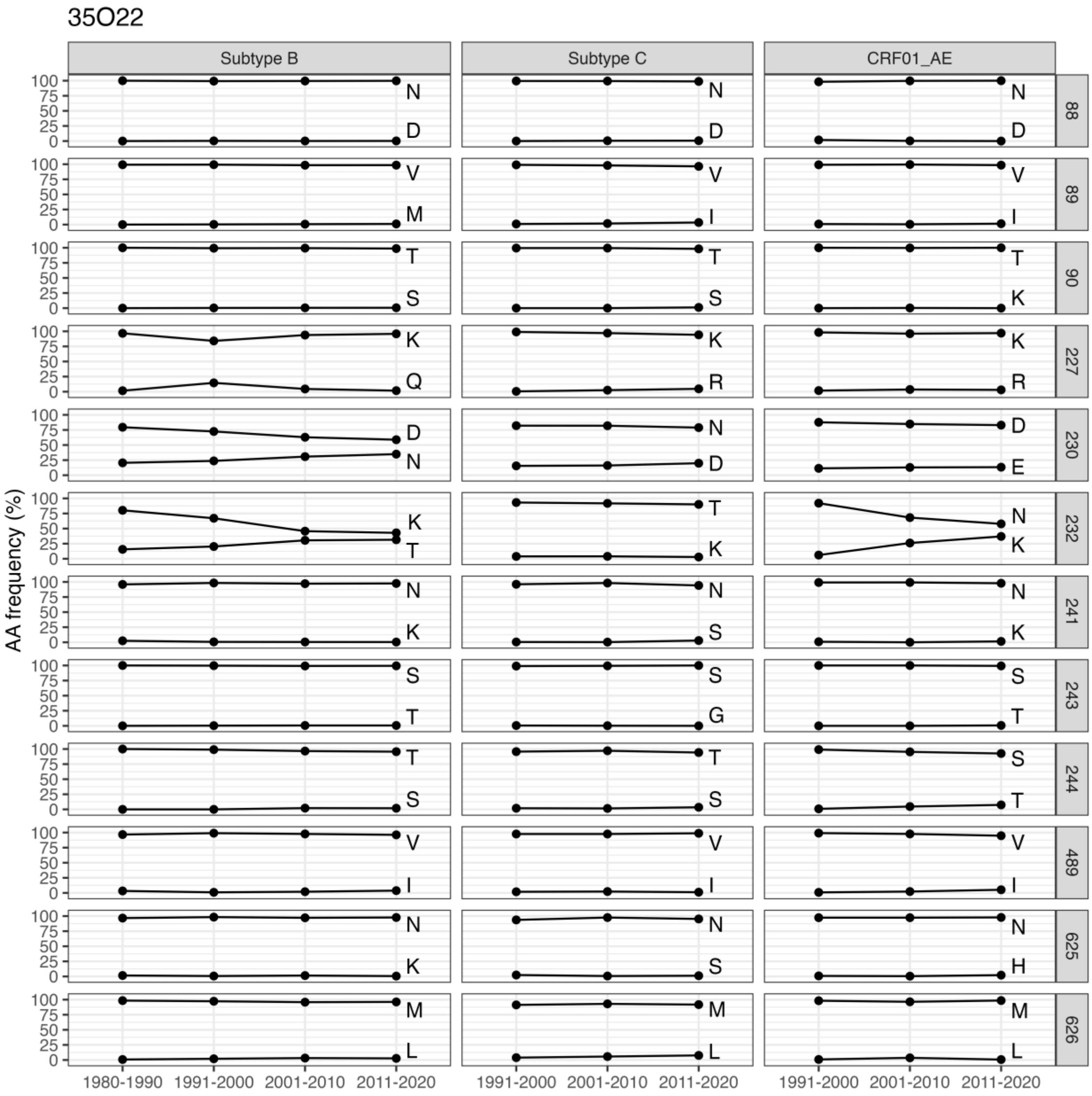

Fig. S23. Dominant residues at 35O22 Ab epitope sites in subtypes B and C and CRF01_AE. The frequency of the two most common residues over time at 35O22 Ab epitope sites in alignments of subtypes B and C and CRF01_AE. Numbers on the right-hand side refer to HXB2 AA sites.

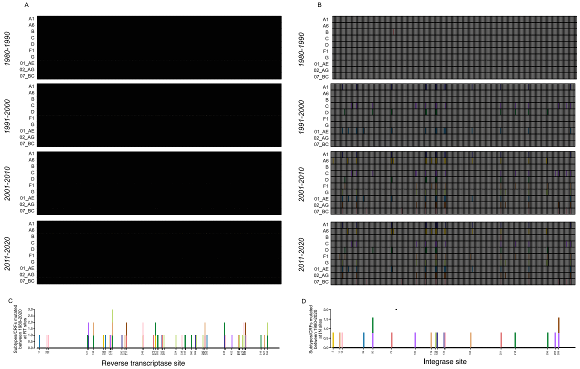

Fig. S24. Diversity over time in HIV-1 Reverse transcriptase and Integrase. (A,B) Map of mismatched residues between the reference (A) RT and (B) IN sequence and each subtype/CRF for each sampling period. (C,D) The number of subtypes/CRFs with a mutation across sampling periods at a (C) RT or (D) IN site.

25

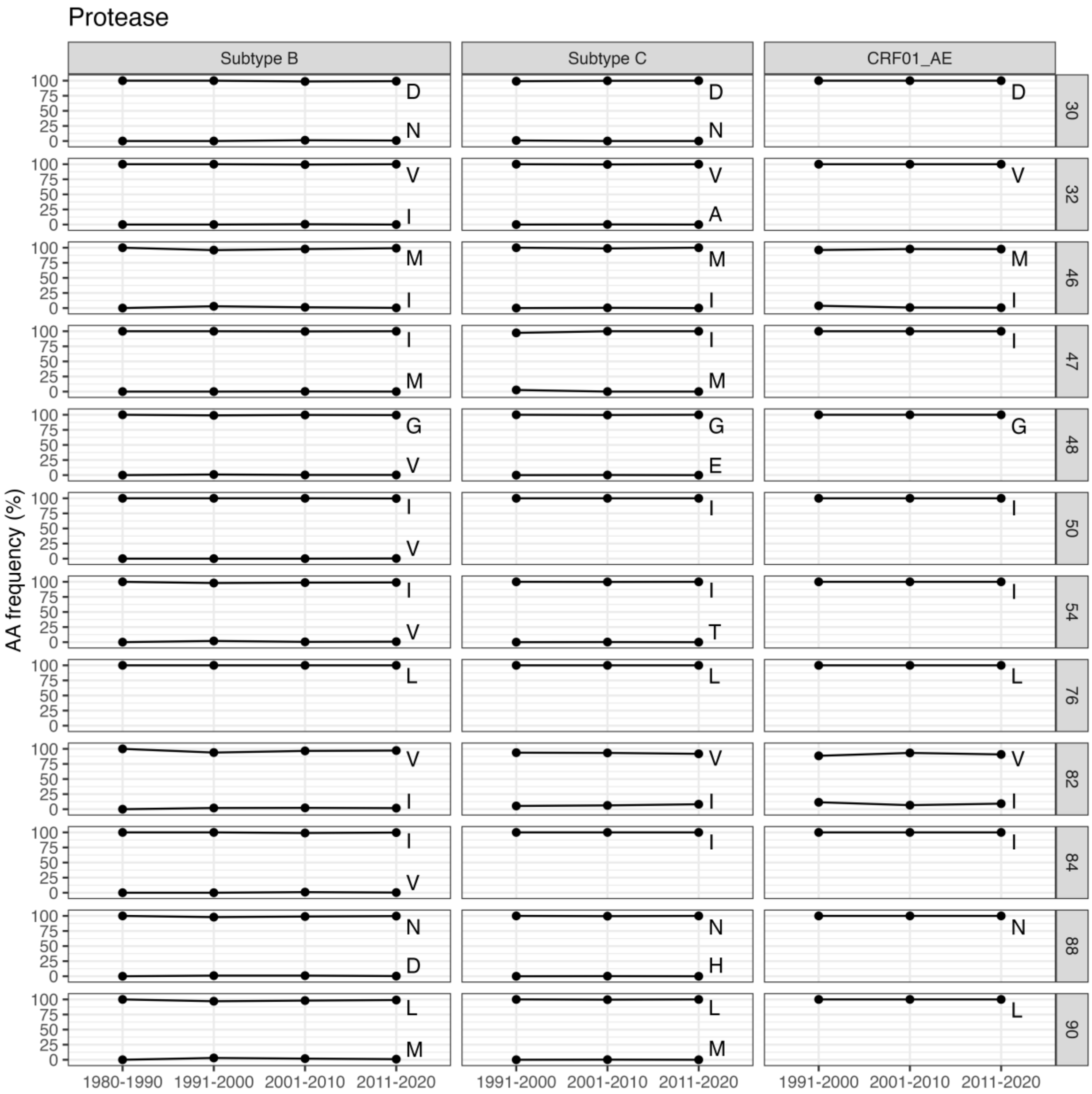

Fig. S25. Dominant residues at Protease sites in subtypes B and C and CRF01_AE. The frequency of the two most common residues over time at sites encoding the Protease enzyme in alignments of subtypes B and C and CRF01_AE. When a single residue was ubiquitous, only the frequency of that residue is shown.

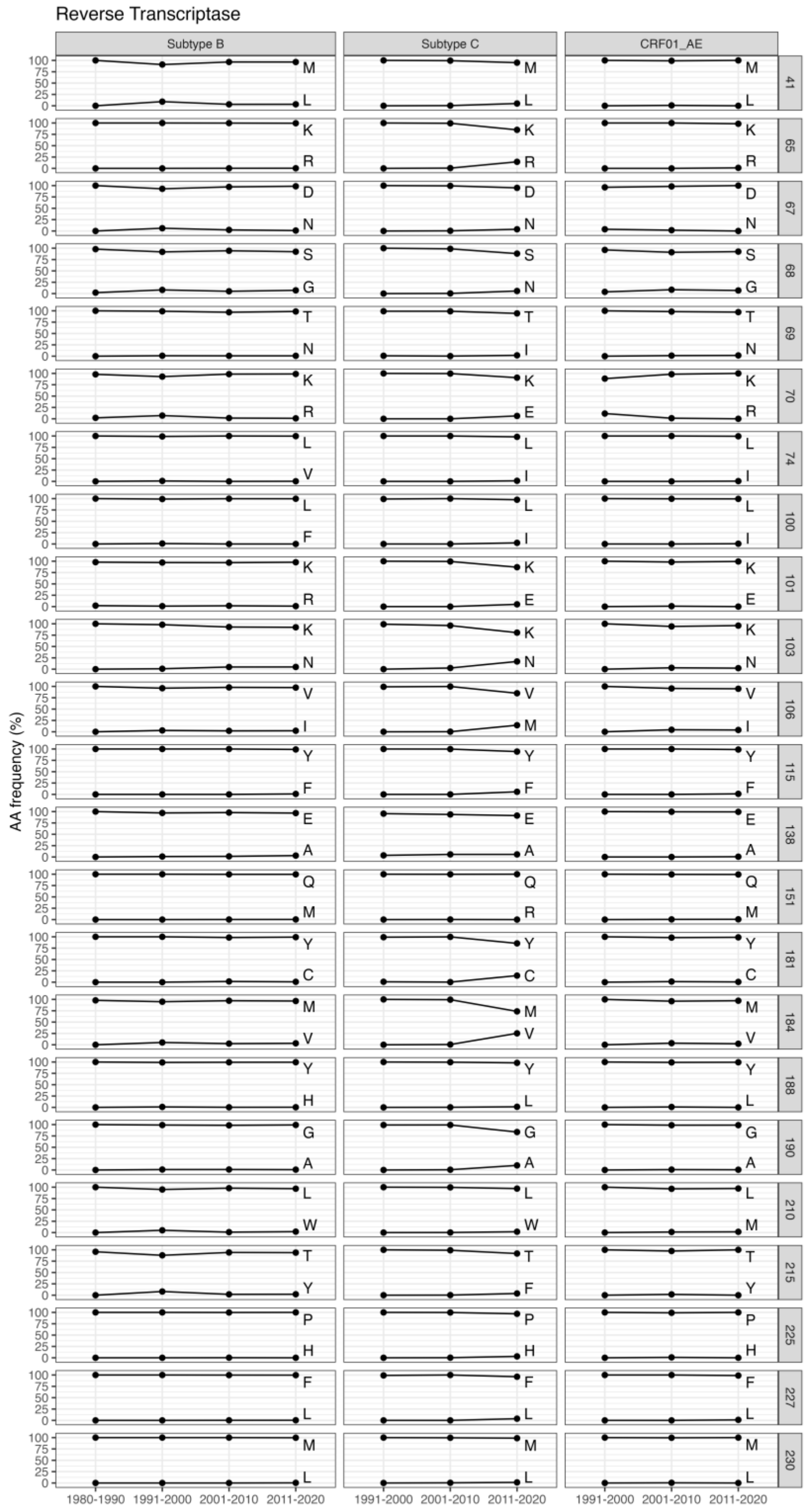

Fig. S26. Dominant residues at Reverse Transcriptase sites in subtypes B and C and CRF01_AE. The frequency of the two most common residues over time at sites encoding the Reverse Transcriptase enzyme in alignments of subtypes B and C and CRF01_AE. When a single residue was ubiquitous, only the frequency of that residue is shown.

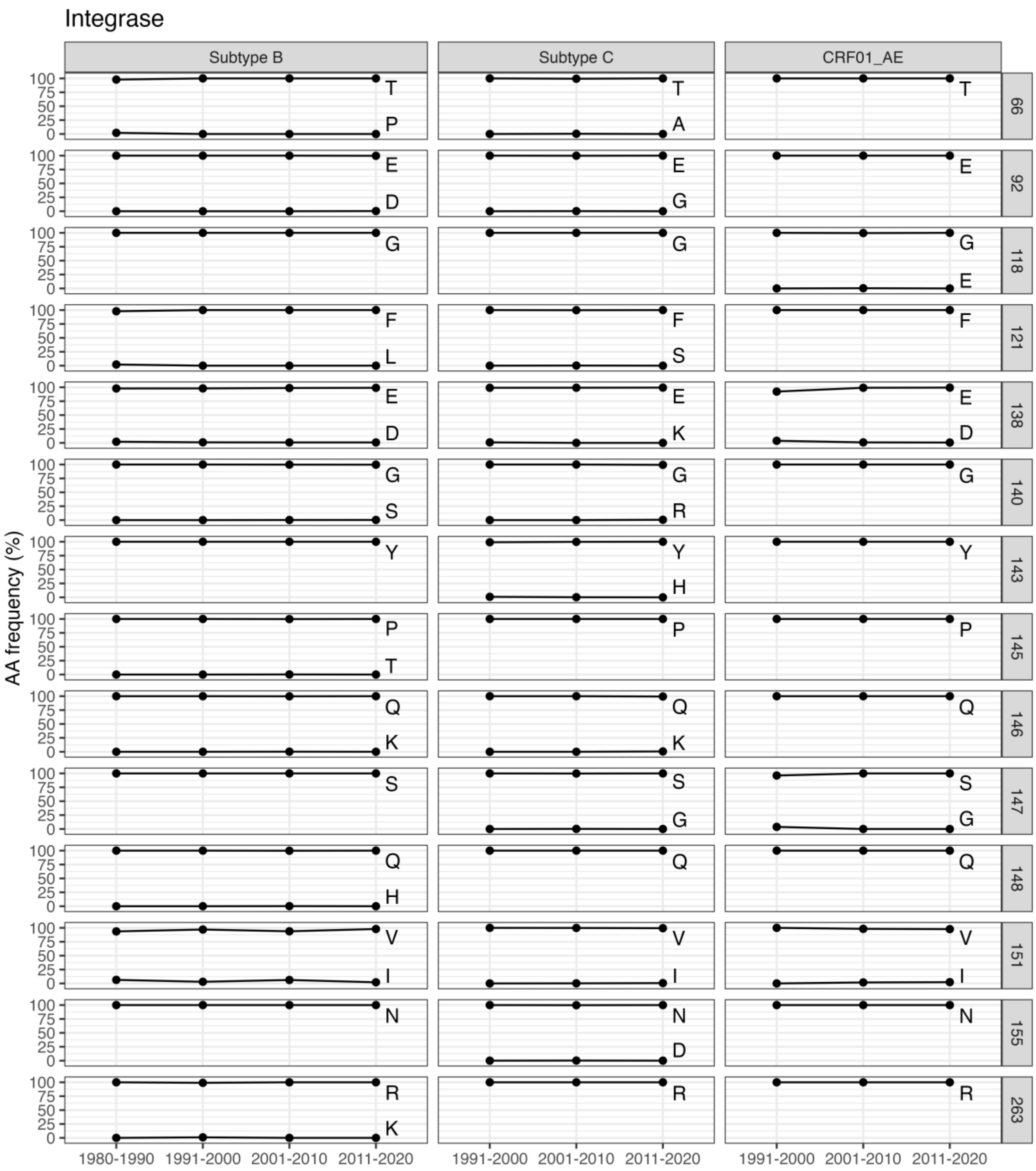

Fig. S27. Dominant residues at Integrase sites in subtypes B and C and CRF01_AE. The frequency of the two most common residues over time at sites encoding the Integrase enzyme in alignments of subtypes B and C and CRF01_AE. When a single residue was ubiquitous, only the frequency of that residue is shown.

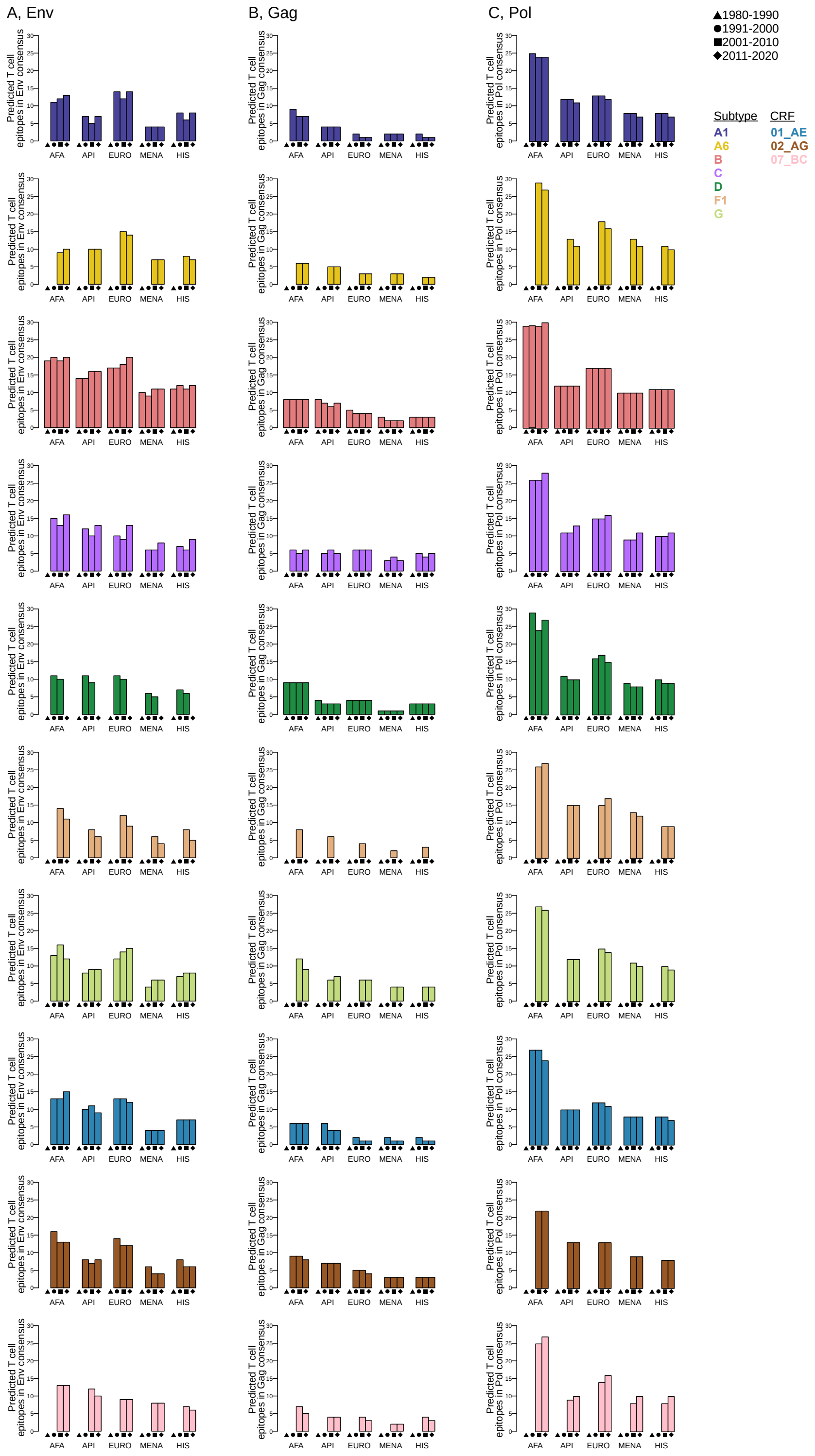

Fig. S28. Predicted CTL epitopes in subtype/CRF consensus sequences. The average number of predicted CTL epitopes in consensus sequences for different sampling periods (see legend) in (A) Env, (B) Gag, and (C) Pol subtypes/CRFs. AFA, African/African-American; API, Asian/Pacific Islands; EURO, European/European descent; MENA, Middle East/North coast of Africa; HIS, South or Central America/Hispanic/Latinx. Subtype/CRF colors correspond to Fig. 1.

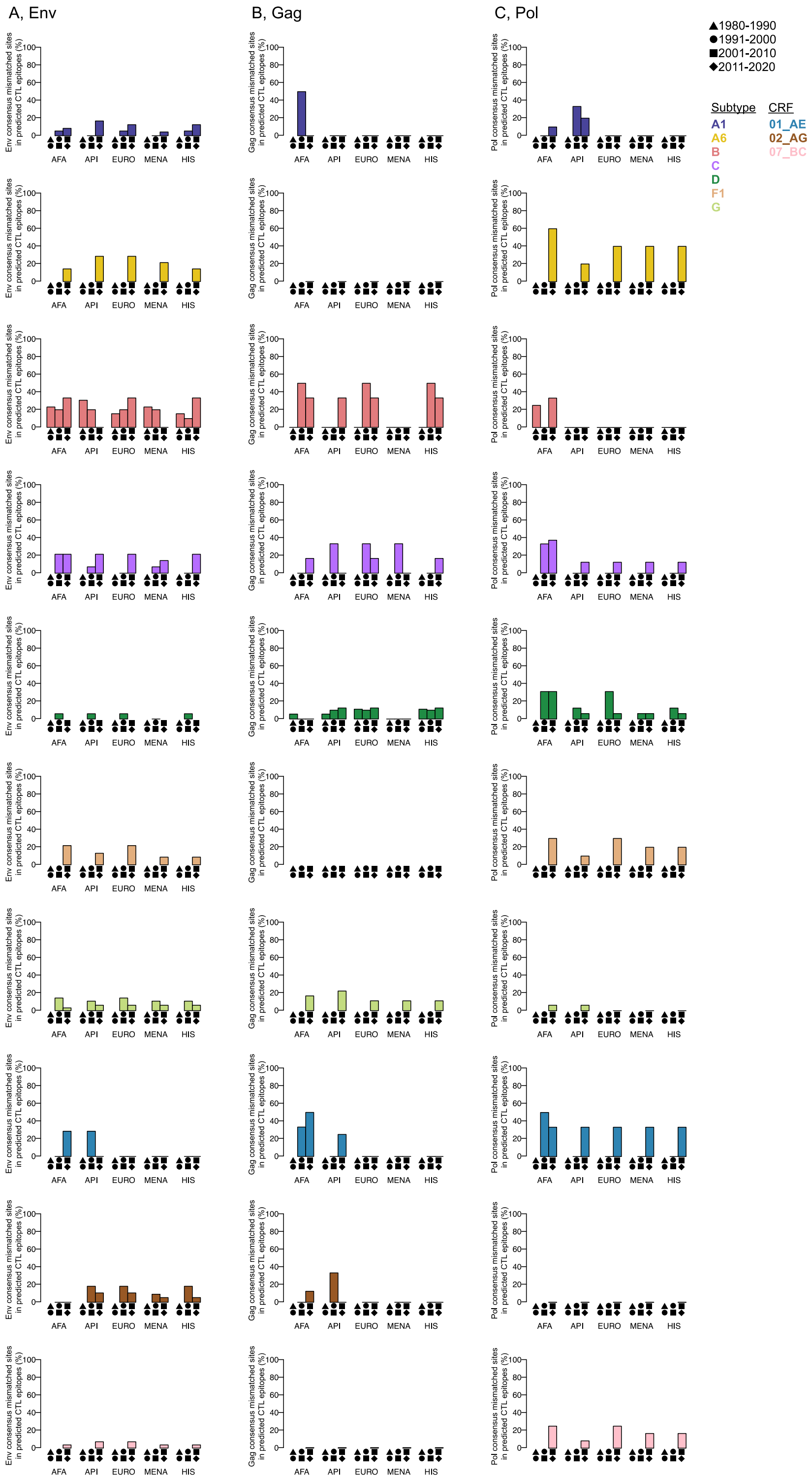

Fig. S29. Subtype/CRF consensus mismatches in predicted CTL epitopes. The number of mismatched sites between subtype/CRF (A) Env, (B) Gag, and (Pol) consensus sequences for different sampling periods in CTL epitopes predicted for five human subpopulations as a percentage of total mismatched sites between subtype/CRF consensus sequence for different sampling periods. Mismatched sites in Env hypervariable loops were omitted. Subtype/CRF colors correspond to Fig. 1.

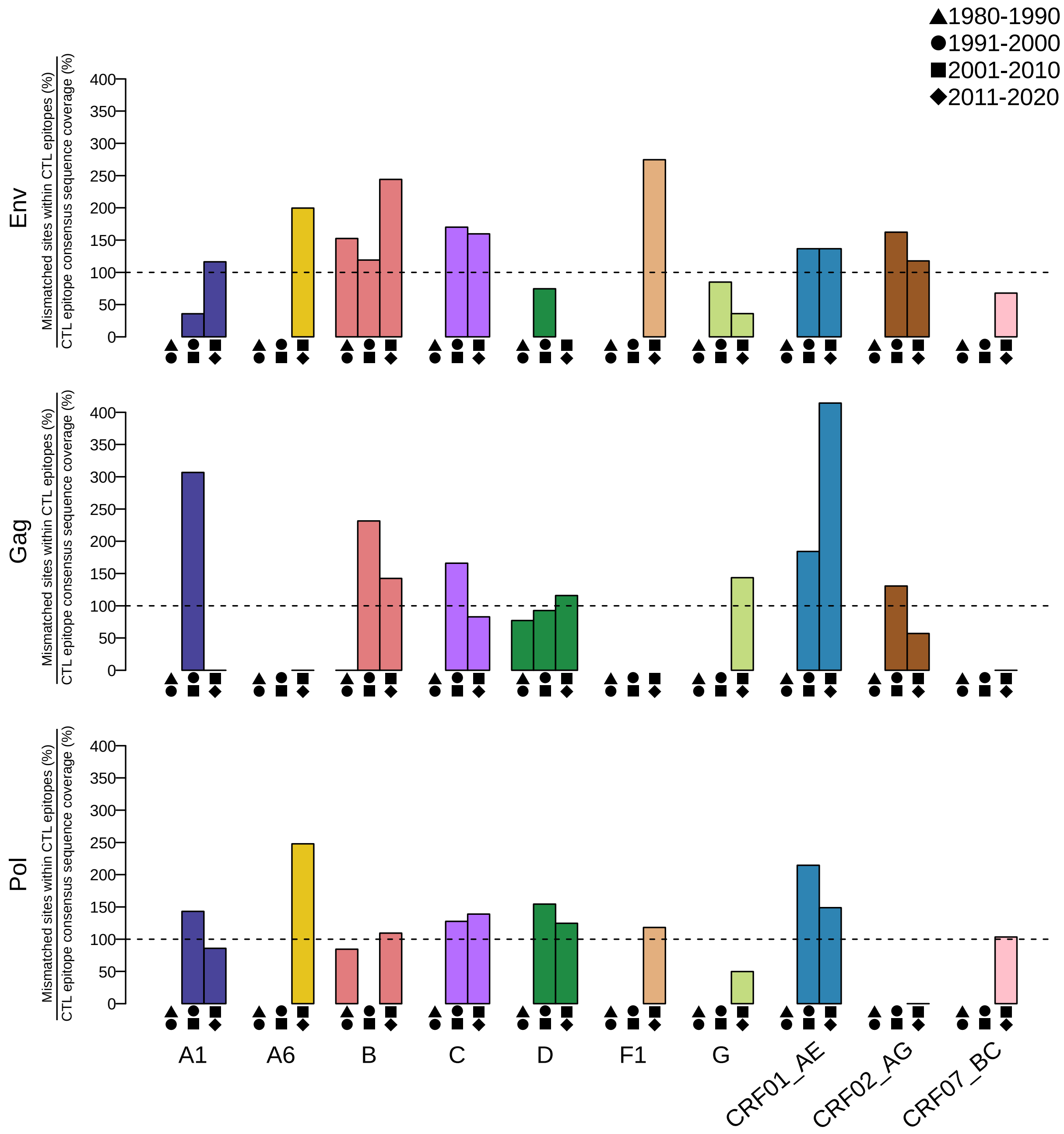

Fig. S30. Subtype/CRF consensus mismatches in predicted CTL epitopes compared to total CTL epitope occurrence in each consensus. The ratio of the percentage of consensus mismatched sites in predicted CTL epitopes between sampling periods (see legend) for each subtype/CRF and the percentage of each consensus sequence covered by predicted CTL epitopes. Values greater than 100% (dashed line) indicate a greater percentage of mismatched sites in CTL epitopes between consensus sequences than the proportion of residues in the consensus sequence within CTL epitopes. Subtype/CRF colors correspond to Fig. 1.

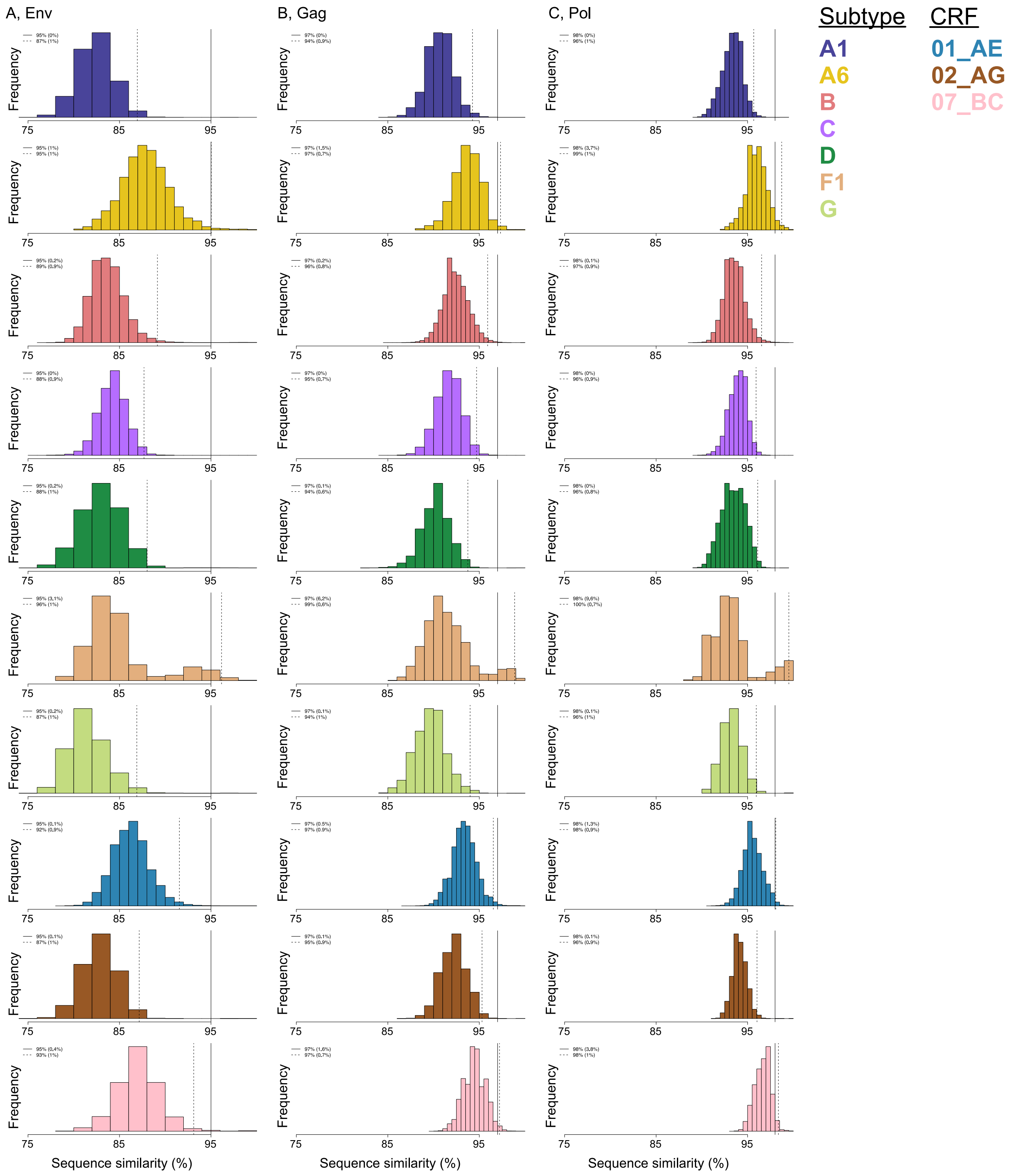

Fig. S31. Distribution of pairwise sequence identity for Env, Gag, and Pol subtypes/CRFs. The distribution of pairwise sequence identity in (A) Env, (B) Gag, and (C) Pol subtypes/CRFs. The solid vertical line indicates a (A) 95%, (B) 97%, or (C) 98% threshold. The percentage of total sequences exceeding the threshold are shown for each subtype/CRF. The dashed line indicates the 99% quantile and the percentage of total sequences exceeding it are shown parenthetically.

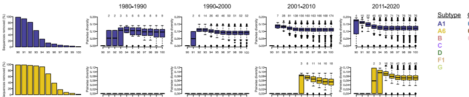

1980-1990
1990-2000
2001-2010
2011-2020
Sequences removed (%)
Pairwise diversity
Subtype
A1
A6
B
C
D
F1
G
CRF
01_AE
02_AG
07_BC

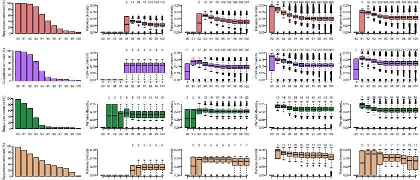

Sequences removed (%)
Pairwise diversity
0 20 40 60 80 100
0.00 0.05 0.10 0.15 0.20 0.25
90 91 92 93 94 95 96 97 98 99 100
2 14 29 74 104 109 112
2 13 37 73 109 153 186 203 207
3 9 38 124 384 650 809 896 940 958 960
3 16 84 229 334 402 423 434 442 453
4 4 4 4 4 4 4
2 6 17 45 85 120 140 149 156 160 163
4 20 83 270 541 712 765 774 780 788 809
2 18 52 113 178 213 215 216 217 220 223
2 2 3 6 9 10 10 10 10 10
2 2 6 13 18 19 19 19 19 19 20
10 16 31 38 40 40 40 40 43 45
7 19 36 53 54 54 54 54 54 54
2 3 6 6 6 6
2 3 5 5 6 6 6 7 7 7
5 11 17 22 25 27 27 27 28 35
2 3 3 4 7 7 7 9 16 17

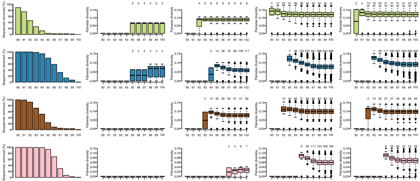

Sequences removed (%)
Pairwise diversity
90 91 92 93 94 95 96 97 98 99 100

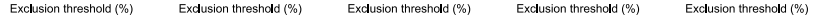

Fig. S32. Subtype/CRF Env diversity at different exclusion thresholds. (A) The percentage of sequences removed for each subtype/CRF at different exclusion thresholds. Boxplots of pairwise diversity estimates at different exclusion thresholds for sequences sampled between (B) 1980-1990, (C) 1991-2000, (D) 2001-2010, and (E) 2011-2020. The total number of sequences at each exclusion threshold are shown above the boxplots.

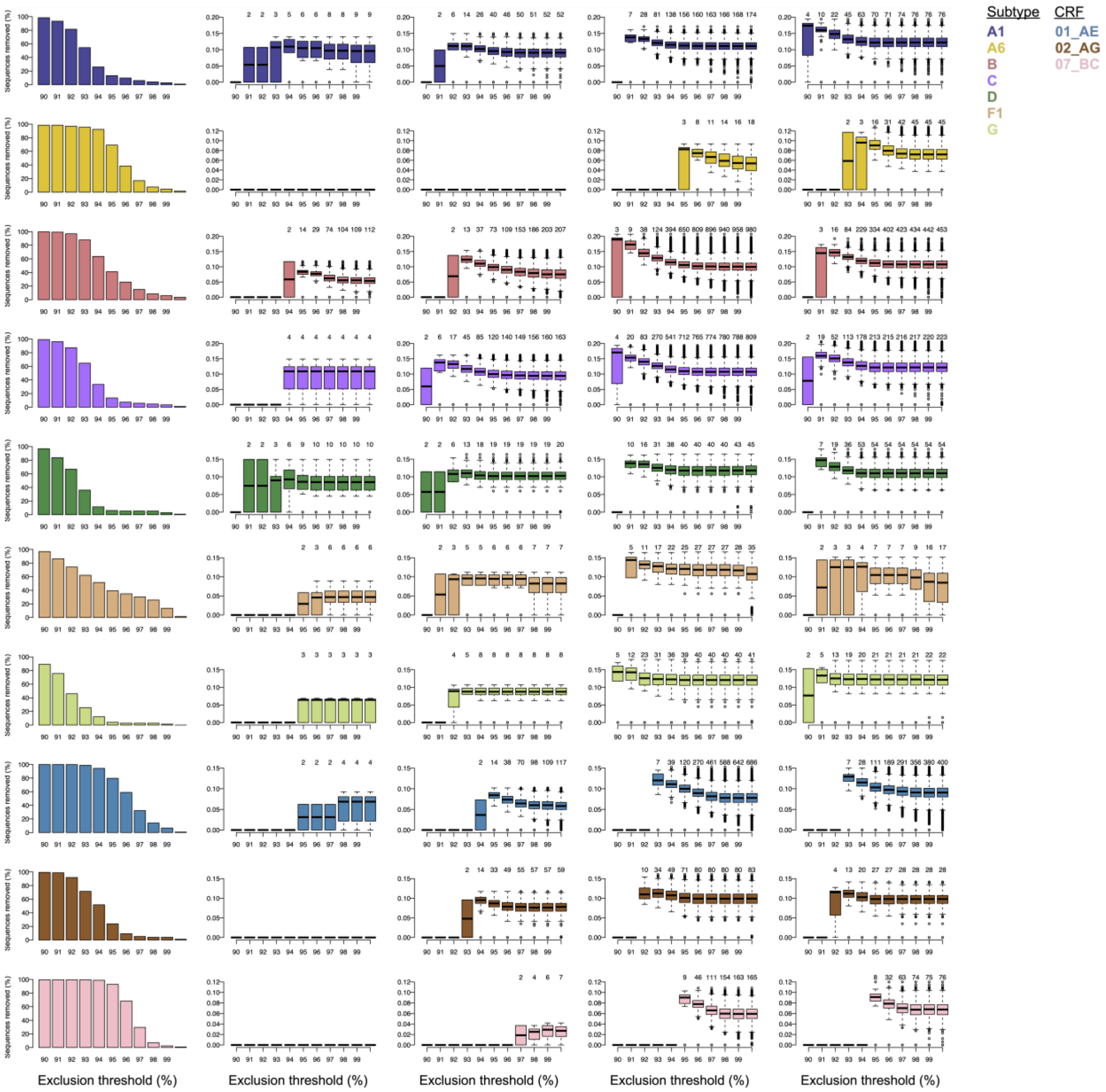

Fig. S33. Subtype/CRF Gag diversity at different exclusion thresholds. (A) The percentage of sequences removed for each subtype/CRF at different exclusion thresholds. Boxplots of pairwise diversity estimates at different exclusion thresholds for sequences sampled between (B) 1980-1990, (C) 1991-2000, (D) 2001-2010, and (E) 2011-2020. The total number of sequences at each exclusion threshold are shown above the boxplots.

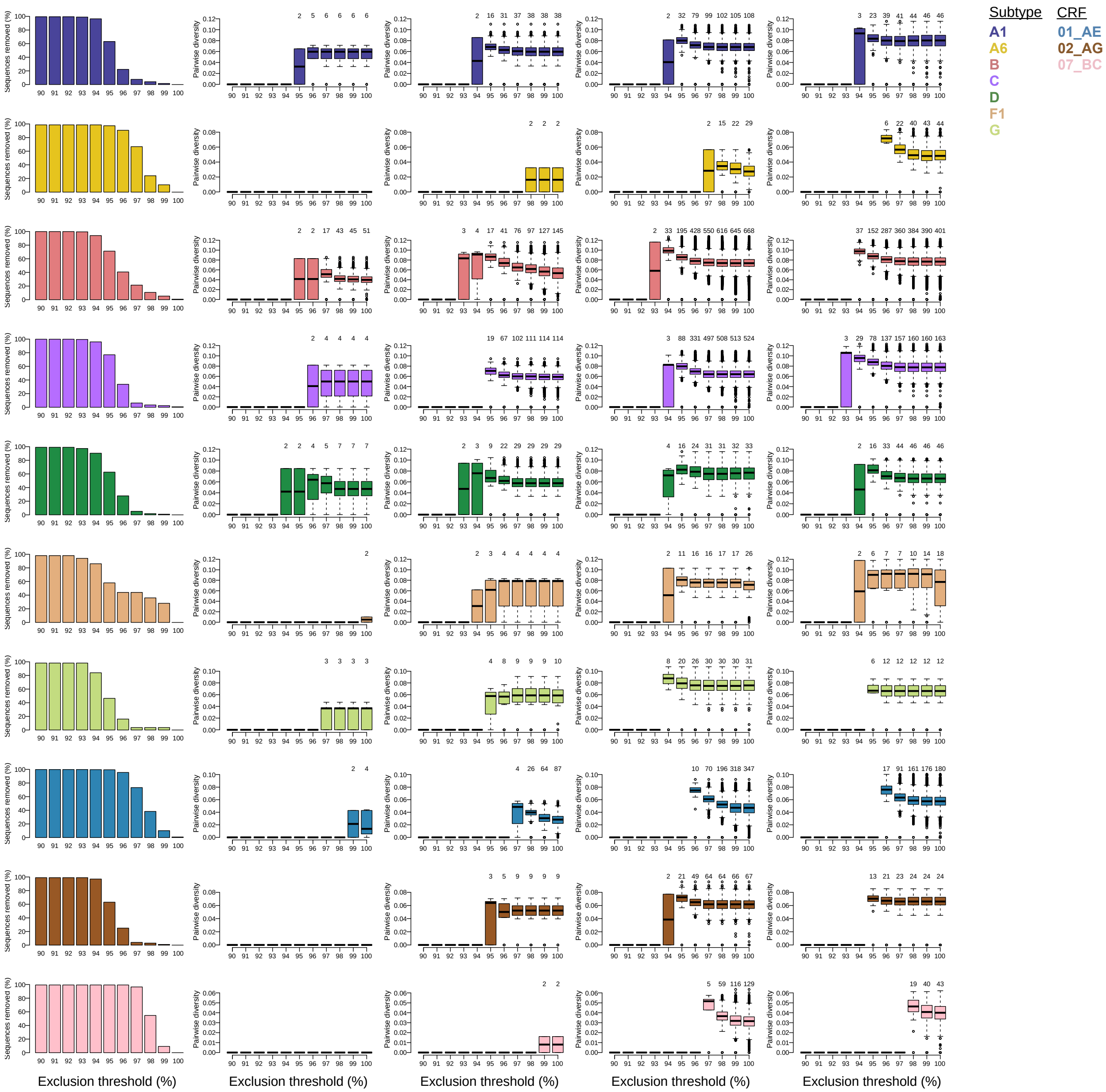

Fig. S34. Subtype/CRF Pol diversity at different exclusion thresholds. (A) The percentage of sequences removed for each subtype/CRF at different exclusion thresholds. Boxplots of pairwise diversity estimates at different exclusion thresholds for sequences sampled between (B) 1980-1990, (C) 1991-2000, (D) 2001-2010, and (E) 2011-2020.

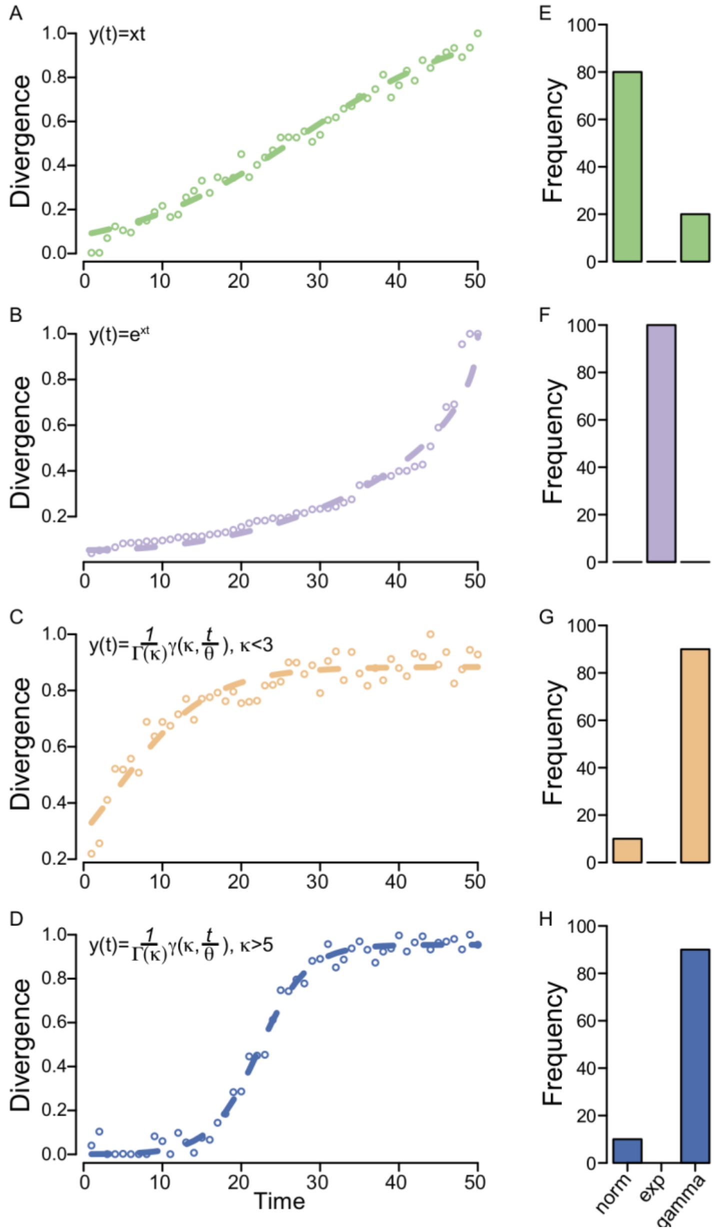

Fig. S35. Growth model recovery on simulated divergence data. Examples of simulated divergence data under a (A) normal model ($\alpha = 0.06$), (B) exponential model ($\beta = 0.075$), (C) gamma model with $\kappa = 1$, and (D) gamma model with $\kappa = 5$. The frequency of recovered models for divergence data simulated under (E) normal models, (F) exponential models, (G) gamma models with $\kappa < 3$, and (H) gamma models with $\theta > 3$. Data simulated under normal, gamma, and exponential models were recovered with >90% accuracy.

Table S1. HIV-1 subtype and CRF sequence data collection by decade and geographical region.

	Sample period	Eastern Asia	South-eastern Asia	Southern Asia	Central Asia	Western Asia	Eastern Africa	Southern Africa	Middle Africa	Western Africa	Australia and New Zealand	Eastern Europe	Northern Europe	Southern Europe	Western Europe	Northern America	Latin America and the Caribbean
**Env**	1980-1990	7	2	0	0	0	7	3	13	2	2	0	3	10	32	64	3
	1991-2000	63	114	24	0	6	136	127	11	21	10	2	28	6	56	139	34
	2001-2010	461	195	9	4	78	453	570	91	35	4	77	105	80	87	493	196
	2011-2020	97	120	56	0	0	74	69	5	25	0	40	35	35	49	296	66
**Gag**	1980-1990	1	1	0	0	0	8	6	13	1	2	0	1	1	6	62	9
	1991-2000	49	69	32	0	1	70	93	5	53	19	2	7	41	7	44	10
	2001-2010	612	269	9	0	77	450	376	71	35	12	24	46	74	76	238	184
	2011-2020	290	182	69	0	0	179	41	4	24	0	48	125	24	30	190	35
**Pol**	1980-1990	0	1	0	0	0	5	5	12	2	2	0	2	1	7	28	4
	1991-2000	27	29	19	0	7	70	77	6	3	13	2	5	3	6	52	10
	2001-2010	347	72	10	1	61	319	240	68	30	4	27	47	32	46	193	179
	2011-2020	97	138	34	0	0	138	37	5	20	0	47	121	51	34	168	38

Table S2. The best-fit growth model and number of sequences at each time period for HIV-1 subtype and CRF alignments of Env, Gag, and Pol.

Protein	*Variable*	*A1*	*A6*	*B*	*C*	*D*	*F1*	*G*	*CRF01_AE*	*CRF02_AG*	*CRF07_BC*
**Env**	Growth model	Normal	Gamma	Gamma	Gamma	Gamma	Normal	Gamma	Normal	Normal	Gamma
**No. sequences**	1980-1990	8	0	122	4	9	1	1	3	0	0
	1991-2000	57	3	295	207	52	6	10	114	31	2
	2001-2010	124	58	1132	953	53	22	49	387	98	62
	2011-2020	53	39	473	171	8	27	12	135	27	22
**Gag**	Growth model	Normal	Normal	Gamma	Normal	Normal	Gamma	Gamma	Normal	Gamma	Gamma
**No. sequences**	1980-1990	8	0	79	4	10	6	2	2	0	0
	1991-2000	50	1	150	141	17	7	6	71	56	3
	2001-2010	162	11	879	719	39	28	38	482	78	117
	2011-2020	70	45	428	206	52	8	20	324	27	61
**Pol**	Growth model	Normal	Gamma	Gamma	Normal	Gamma	Normal	Gamma	Gamma	Gamma	Normal
**No. sequences**	1980-1990	6	0	47	4	7	1	3	1	0	0
	1991-2000	38	2	99	111	29	4	9	26	9	2
	2001-2010	103	15	624	508	31	18	30	223	64	60
	2011-2020	44	43	390	159	46	11	12	173	24	26

Table S3. Consensus mutations over sampling periods at VRC01 Ab epitope sites in each subtype/CRF. The residues at mutated sites are listed by sampling period. 1980-1990, 1991-2000, 2001-2010, 2011-2020. Asterisks represent sampling times with no data. Only sites with mutations are shown.

	279	281	283	365	460	461	462	463	464	465
A1	*NND	-	*TNN	-	-	-	-	*SNN	-	-
A6	-	**GS	-	-	-	**TN	-	-	-	-
B	DDND	-	-	-	-	-	NNNT	-	-	-
C	*NDN	-	-	-	-	*TNN	-	-	-	-
D	-	-	-	-	**AN	-	-	-	**QS	-
F1	-	-	-	-	-	-	-	-	-	-
G	-	*AAT	*VTV	*TST	-	-	-	*SNT	*TTN	*NTN
01_AE	-	-	-	-	-	-	-	TNN	-	-
02_AG	-	-	-	-	-	-	-	-	-	-
07_BC	-	**VA	-	-	**TN	**NS	**EN	-	-	-

Table S4. Consensus mutations over sampling periods at CAP256-VRC26.25 Ab epitope sites in each subtype/CRF. The residues at mutated sites are listed by sampling period. 1991-2000, 2001-2010, 2011-2020. Asterisks represent sampling times with no data. Only sites with mutations are shown.

	166	168	169
A1	-	-	-
A6	-	-	-
B	-	-	-
C	-	-	-
D	-	-	-
F1	-	-	**KQ
G	*KRR	*RKK	-
01_AE	-	-	-
02_AG	-	-	-
07_BC	-	**RK	-

Table S5. Consensus mutations over sampling periods at PGT121 Ab epitope sites in each subtype/CRF. The residues at mutated sites are listed by sampling period. 1980-1990, 1991-2000, 2001-2010, 2011-2020. Asterisks represent sampling times with no data. Only sites with mutations are shown.

	308	309	315	319
A1	*RHR	-	-	-
A6	-	-	-	-
B	-	-	-	TAAA
C	-	-	-	-
D	-	-	-	-
F1	-	**IL	**QR	-
G	-	-	-	-
01_AE	*TTR	-	-	-
02_AG	-	-	-	-
07_BC	-	-	-	-

Table S6. Consensus mutations over sampling periods at 10E8 Ab epitope sites in each subtype/CRF. The residues at mutated sites are listed by sampling period. 1991-2000, 2001-2010, 2011-2020. Asterisks represent sampling times with no data. Only sites with mutations are shown.

	676	677	683
A1	-	-	-
A6	-	-	-
B	-	-	-
C	-	-	-
D	-	**KN	-
F1	-	-	-
G	*TSS	-	*RKK
01_AE	-	-	-
02_AG	-	-	-
07_BC	-	-	-

Table S7. Consensus mutations over sampling periods at 35O22 Ab epitope sites in each subtype/CRF. The residues at mutated sites are listed by sampling period. 1991-2000, 2001-2010, 2011-2020. Asterisks represent sampling times with no data. Only sites with mutations are shown.

	232
A1	-
A6	-
B	-
C	-
D	-
F1	**TK
G	-
01_AE	-
02_AG	-
07_BC	**TK

File S1. Phylogeny for Env sequences sampled between 1980-2020. The tree was constructed based on 4,830 Env protein sequences using the HIV between-patient matrix HIV-B substitution model with rate heterogeneity across sites (FreeRate model with 10 classes) (best model as defined with ModelFinder implemented in IQ-Tree).

File S2. Phylogeny for Gag sequences sampled between 1980-2020. The tree was constructed based on 4,407 Gag protein sequences using the Jones-Taylor-Thornton (JTT) model with rate heterogeneity across sites (FreeRate model with 10 classes) (best model as defined with ModelFinder implemented in IQ-Tree).

File S3. Phylogeny for Pol sequences sampled between 1980-2020. The tree was constructed based on 3,002 Pol protein sequences using the HIV between-patient matrix HIV-B substitution model with rate heterogeneity across sites (FreeRate model with 10 classes) (best model as defined with ModelFinder implemented in IQ-Tree).

File S4. Env consensus sequences for each subtype/CRF and sampling period. Suffixes in the consensus sequence name indicate the subtype/CRF and decade during which sequences were sampled.

>Env_CON_A1_1990s
MRVMGIQRNCQHLLRWGTMILGMIIICSAAENLWVTVYYGVPVWKDAETTLFCASDAKAYETEVHNVWATHACVPTDPNPQEIHLEN
VTEEFNMWKNNMVEQMHTDIISLWDQSLKPCVKLTPLCVTLNCSNVNTNNTNTNITSNMKGEIKNCSFNMTTELRDKKQKVYSLFYR
LDVVQINENNNNSSSSEYRLINCNTSAITQACPKVSFEPIPIHYCAPAGFAILKCNDKEFNGTGPCKNVSTVQCTHGIKPVVSTQLLLNG
SLAEGEVKIRSENITNNAKTIIVQLVEPVEINCTRPNNNTRKSIRIGPGQAFYATGDIIGDIRQAHCNVSRSEWNKTLQQVATQLRKYFG
NKTIIFTNSSGGDLEITTHSFNCGGEFFYCNTSGLFNSTWNNNSTQESNSTESNDTITLPCRIKQIINMWQRVGQAMYAPPIQGVIRCE
SNITGLILTRDGGNNNSTNETFRPGGGDMRDNWRSELYKYKVVKIEPLGVAPTRAKRRVVEREKRAVGIGAVFLGFLGAAGSTMGAA
SITLTVQARQLLSGIVQQQSNLLRAIEAQQHLLKLTVWGIKQLQARVLAVERYLKDQQLLGIWGCSGKLICTTNVPWNSSWSNKSQSEI
WDNMTWLQWDKEISNYTQIIYNLIEESQNQQEKNEQDLLALDKWANLWNWFDISNWLWYIKIFIMIVGGLIGLRIVFAVLSVINRVRQG
YSPLSFQTHTPNPRGLDRPGRIEEEGGEQDRDRSIRLVSGFLALAWDDLRSLCLFSYHRLRDFILIAARTVELLGHSSLKGLRLGWEG
LKYLWNLLLYWGRELKISAINLLDTIAIAVAGWTDRVIEIGQRIGRAILHIPRRIRQGFERALL
>Env_CON_A1_2000s
MRVMGIQRNCQHLLRWGTMILGMIIICSAAENLWVTVYYGVPVWKDAETTLFCASDAKAYDTEVHNVWATHACVPTDPNPQEIHLEN
VTEEFNMWKNNMVEQMHTDIISLWDQSLKPCVKLTPLCVTLNCSNVNTTNNNTTNNNITTEMKEEIKNCSFNMTTELRDKKQKVYSL
FYRLDIVQINENSNNNSSEYRLINCNTSAITQACPKVSFEPIPIHYCAPAGFAILKCNDKEFNGTGPCKNVSTVQCTHGIKPVVSTQLLL
NGSLAEGEVMIRSENITNNAKNIIVQLNKPVNINCTRPNNNTRKSVHIGPGQAFYATGDIIGDIRQAHCNVSRTEWNKTLQQVAEQLRK
HFGNKTIIFNNSSGGDLEITTHSFNCGGEFFYCNTSGLFNSTWNNNTTTQESNSTESNDTITLPCRIKQIINMWQRVGQAMYAPPIQGV
IRCESNITGLILTRDGGNNNNTNETFRPGGGDMRDNWRSELYKYKVVKIEPLGVAPTRARRRVVEREKRAVGIGAVFLGFLGAAGST
MGAASITLTVQARQLLSGIVQQQSNLLRAIEAQQHLLKLTVWGIKQLQARVLAVERYLKDQQLLGIWGCSGKLICTTNVPWNSSWSNK
SQDEIWDNMTWLQWDKEISNYTQIIYNLIEESQNQQEKNEQDLLALDKWASLWNWFDISNWLWYIKIFIMIVGGLIGLRIVFAVLSIINRV
RQGYSPLSFQTHTPNPRGLDRPGRIEEEGGEQGRDRSIRLVSGFLALAWDDLRSLCLFSYHRLRDFILIAARTVELLGHSSLKGLRLG
WEGLKYLWNLLLYWGRELKISAINLLDTIAIAVAGWTDRVIEIGQRIGRAILHIPRRIRQGLERALL
>Env_CON_A1_2010s
MRVMGIQRNCQNLLRWGTMILGMIIICSAAENLWVTVYYGVPVWKDAETTLFCASDAKAYETEVHNVWATHACVPTDPNPQEIPLEN
VTEEFNMWKNNMVEQMHTDIISLWDQSLKPCVKLTPLCVTLNCSNVNTNNTNTNSTITNEMREEMKNCSFNMTTELRDKKQKVYSLF
YKLDIVQINENSNNNSSEYRLINCNTSAITQACPKVSFEPIPIHYCAPAGFAILKCNDKEFNGTGPCKNVSTVQCTHGIKPVVSTQLLLN
GSLAEGEVMIRSENITDNAKNIIVQLNKPVKINCTRPNNNTRKSVRIGPGQAFYATGDIIGDIRQAHCNVSKSEWNKTLQQVAKQLRKY
FKNKTIIFTNSSGGDLEITTHSFNCGGEFFYCNTSGLFNSTWNNNGTNSSNSTESNDTITLPCRIKQIINMWQRVGQAMYAPPIPGVIR
CVSNITGLILTRDGGNNNNTNETFRPGGGDMRDNWRSELYKYKVVKIEPLGVAPTRAKRRVVEREKRAVGLGAVFLGFLGAAGSTM
GAASITLTVQARQLLSGIVQQQSNLLRAIEAQQHLLKLTVWGIKQLQARVLAVERYLKDQQLLGIWGCSGKLICTTNVPWNSSWSNKS
YNEIWDNMTWLQWDKEISNYTQIIYRLIEESQNQQEKNEQDLLALDKWASLWNWFDISNWLWYIKIFIMIVGGLIGLRIVFAVLSIINRVR
QGYSPLSFQTHTPNPGGLDRPGRIEEEGGEQDRDRSIRLVSGFLALAWDDLRSLCLFSYHRLRDFILIAARTVELLGHSSLKGLRLGW
EGLKYLWNLLLYWGRELKISAINLFDTIAIAVAGWTDRVIEIGQRIGRAILHIPRRIRQGLERALL
>Env_CON_A6_2000s
MKARGMQRNYQHLWRWGTMLFWMIIMCKAAENLWVTVYYGVPVWRDAETTLFCASDAKAYDKEVHNVWATHACVPTDPNPQEIIL
ENVTENFDMWKNNMVEQMQTDIISLWDQSLKPCVKLTPLCVTLNCTEPNSTSSNNSSVNSNSSDSLFEEMKNCSFNMTTELRDKRK
TVHSLFYKLDIVSTDSNDSGQYRLINCNTSTMTQACPKVTFEPIPIHYCAPAGFAILKCKDTNFNGTGPCKNVSTVQCTHGIKPVVSTQ
LLLNGSLAEKEVMIRSENITDNGKIIVQLTEPVNITCIRPGNNTRTSIRIGPGQTFYATGDVIGDIRKAHCNVSRAAWNSTLQKISTQLRK
YFNNKTIIFKNSSGGDLEVTTHSFNCGGEFFYCNTTDLFNSTWDENSTVTNSTQANETITLPCRIKQIINMWQRVGQAMYAPPIKGSIR
CESNITGLLLTRDGGGGTNSSNETFRPIGGDMRDNWRSELYKYKVVKIEPIGVAPTKAKRRVVEREKRAIGLGAAFLGFLGAAGSTM
GAASITLTVQARQLLSGIVQQQSNLLRAIEAQQHLLKLTVWGIKQLQARVLAVERYLKDQQLLGIWGCSGKLICTTNVPWNSSWSNKS
QSEIWDNMTWMQWDKEVINYTNIIYDLIEESQNQQEKNEQDLLALDKWASLWNWFDISNWLWYIKIFIIIVGGLIGLRIVFAVLSIINRAR
QGYSPLSLQTLTPHPEGPDRPGRIKEEGGEQGRDRSIRLVSGFLALAWDDLRSLCLFSYHRLRDFISIAARTVELLGRSSLKGLRLGW
EGLKYLGNLLGYWGQELKSSAINLIDTIAIAVAGWTDRVIEIGQRFCRAIRNIPRRIRQGAEKALQ
>Env_CON_A6_2010s
MKARGMQRNYQHLWRWGTMLFWMIIMCKAAENLWVTVYYGVPVWRDAETTLFCASDAKAYDKEVHNVWATHACVPTDPNPQEID
LENVTEEFNMWKNNMVEQMQTDIISLWDQSLKPCVKLTPLCVTLNCTEPNSTSSNNSSSSNSSSSLYEEMKNCSFNMTTELRDKRK
TVYSLFYKLDIVSTDSNNSSGQYRLINCNTSTMTQACPKVTFEPIPIHYCAPAGFAILKCKDPNFNGTGPCKNVSTVQCTHGIKPVVST
QLLLNGSLAEKEVMIRSENITDNSKIIVQLTEPVNITCIRPGNNTRTSIRIGPGQTFYATGDVIGDIRKAYCNVSRTAWNSTLQKISTQLR
KYFNNKTIIFKNSSGGDLEVTTHSFNCGGEFFYCNTTALFNSTWDGNSTVTNSTQTNETITLPCRIKQIINMWQRVGQAMYAPPIKGNI
RCESNITGLLLTRDGGGNNSSNETFRPIGGDMRDNWRSELYKYKVVKIEPIGVAPTKAKRRVVEREKRAIGLGAAFLGFLGAAGSTM
GAASITLTVQARQLLSGIVQQQSNLLRAIEAQQHLLKLTVWGIKQLQARVLAVERYLKDQQLLGIWGCSGKLICTTNVPWNSSWSNKS
QNEIWDNMTWMQWDKEVINYTNIIYDLIEESQNQQEKNEQDLLALDKWASLWNWFDISNWLWYIKIFIMIVGGLIGLRIVFAVLSIINRV
RQGYSPLSLQTLTPHPEGPDRPGRIKEEGGEQGRDRSIRLVSGFLALAWDDLRSLCLFSYHRLRDFISIAARTVELLGRSSLKGLRLG
WEGLKYLGNLLGYWGQELKSSAINLIDTIAIAVAGWTDRVIEIGQRFCRAICNIPRRIRQGAEKALQ
>Env_CON_B_1980s
MRVKGIRKNYQHLWRWGTMLLGMLMICSAAEKLWVTVYYGVPVWKEATTTLFCASDAKAYDTEVHNVWATHACVPTDPNPQEVVL
ENVTENFNMWKNNMVEQMHEDIISLWDQSLKPCVKLTPLCVTLNCTDLRNNTNTTNSSSGGTMEKGEIKNCSFNITTSIRDKVQKEY
ALFYKLDVVPIDNDNTSYRLISCNTSVITQACPKVSFEPIPIHYCAPAGFAILKCNDKKFNGTGPCTNVSTVQCTHGIRPVVSTQLLLNG
SLAEEEVVIRSENFTDNAKTIIVQLNESVEINCTRPNNNTRKSIHIGPGRAFYTTGEIIGDIRQAHCNISRAKWNNTLKQIVEKLREQFGN
KTIVFNQSSGGDPEIVMHSFNCGGEFFYCNSTQLFNSTWNSTETTTNNNNTNDTITLPCRIKQIINMWQEVGKAMYAPPIRGQIRCSS
NITGLLLTRDGGNNNNNTTEIFRPGGGDMRDNWRSELYKYKVVKIEPLGVAPTKAKRRVVQREKRAVGIGAMFLGFLGAAGSTMGA
ASMTLTVQARLLLSGIVQQQNNLLRAIEAQQHLLQLTVWGIKQLQARVLAVERYLKDQQLLGIWGCSGKLICTTAVPWNASWSNKSLD
EIWDNMTWMEWEREIDNYTSLIYTLIEESQNQQEKNEQELLELDKWASLWNWFDITNWLWYIKIFIMIVGGLVGLRIVFAVLSIVNRVR

QGYSPLSFQTRLPAPRGPDRPEGIEEEGGERDRDRSGRLVDGFLALIWVDLRSLCLFSYHRLRDLLLIVTRIVELLGRRGWEALKYW
WNLLQYWSQELKNSAVSLLNATAIAVAEGTDRVIEVLQRAYRAILHIPRRIRQGLERALL
>Env_CON_B_1990s
MRVKGIRKNYQHLWRWGTMLLGMLMICSAAEQLWVTVYYGVPVWKEATTTLFCASDAKAYDTEVHNVWATHACVPTDPNPQEVVL
ENVTENFNMWKNNMVEQMHEDIISLWDQSLKPCVKLTPLCVTLNCTDLNNNTTTNNTTNSSSGGKMEKGEIKNCSFNITTSIRDKVQ
KEYALFYKLDVVPIDNDNTSYRLISCNTSVITQACPKVSFEPIPIHYCAPAGFAILKCNDKKFNGTGPCTNVSTVQCTHGIRPVVSTQLLL
NGSLAEEEVVIRSENFTDNAKTIIVQLNESVEINCTRPNNNTRKSIHIGPGRAFYATGDIIGDIRQAHCNISRAKWNNTLKQIVIKLREQF
GNKTIVFNQSSGGDPEIVMHSFNCGGEFFYCNTTQLFNSTWNGNSTTNNTTEGNDTITLPCRIKQIINMWQEVGKAMYAPPIRGQIRC
SSNITGLLLTRDGGNNNNNTTETFRPGGGDMRDNWRSELYKYKVVKIEPLGVAPTKAKRRVVQREKRAVGIGAMFLGFLGAAGSTM
GAASMTLTVQARQLLSGIVQQQNNLLRAIEAQQHLLQLTVWGIKQLQARVLAVERYLKDQQLLGIWGCSGKLICTTAVPWNTSWSNK
SLDDIWDNMTWMEWEREIDNYTSLIYTLIEESQNQQEKNEQELLELDKWASLWNWFDITNWLWYIKIFIMIVGGLIGLRIVFAVLSIVNR
VRQGYSPLSFQTHLPAPRGPDRPEGIEEEGGERDRDRSGRLVDGFLALIWVDLRSLCLFSYHRLRDLLLIVARIVELLGRRGWEALKY
WWNLLQYWSQELKNSAVSLLNATAIAVAEGTDRVIEVLQRAFRAILHIPRRIRQGLERALL
>Env_CON_B_2000s
MRVKGIRKNYQHLWRWGTMLLGMLMICSAAEQLWVTVYYGVPVWKEATTTLFCASDAKAYDTEVHNVWATHACVPTDPNPQEVVL
ENVTENFNMWKNNMVEQMHEDIISLWDQSLKPCVKLTPLCVTLNCTDLNNTTTNNNTTNSSSSTMEKGEIKNCSFNITTSIRDKVQKE
YALFYKLDVVPIDNNNNTSYRLISCNTSVITQACPKVSFEPIPIHYCAPAGFAILKCNDKKFNGTGPCTNVSTVQCTHGIRPVVSTQLLL
NGSLAEEEVVIRSENFTNNAKTIIVQLNESVEINCTRPNNNTRKSIHIGPGRAFYATGDIIGDIRQAHCNISRTKWNNTLKQIVKKLREQF
GNKTIVFNQSSGGDPEIVMHSFNCGGEFFYCNTTQLFNSTWNNNGTNTNNNTNGNDTITLPCRIKQIINMWQEVGKAMYAPPIRGQI
RCSSNITGLLLTRDGGNNNNNTTETFRPGGGDMRDNWRSELYKYKVVKIEPLGVAPTKAKRRVVQREKRAVGIGAMFLGFLGAAGS
TMGAASITLTVQARQLLSGIVQQQNNLLRAIEAQQHLLQLTVWGIKQLQARVLAVERYLKDQQLLGIWGCSGKLICTTAVPWNTSWSN
KSLDEIWDNMTWMQWEREIDNYTGLIYTLIEESQNQQEKNEQELLELDKWASLWNWFDITNWLWYIKIFIMIVGGLIGLRIVFAVLSIVN
RVRQGYSPLSFQTRLPAPRGPDRPEGIEEEGGERDRDRSGRLVDGFLALIWVDLRSLCLFSYHRLRDLLLIVARIVELLGRRGWEALK
YWWNLLQYWSQELKNSAVSLLNATAIAVAEGTDRIIEVVQRAFRAILHIPRRIRQGLERALL
>Env_CON_B_2010s
MRVKGIRKNYQHLWRWGTMLLGMLMICSAAEQLWVTVYYGVPVWKEATTTLFCASDAKAYDTEVHNVWATHACVPTDPNPQEVVL
ENVTENFNMWKNNMVEQMHEDIISLWDQSLKPCVKLTPLCVTLNCTDLNNTTTNNNTNSSSSSTMEKGEIKNCSFNITTSIRDKVQKEY
ALFYKLDVVPIDNNNNNTSYRLISCNTSVITQACPKVSFEPIPIHYCAPAGFAILKCNDKKFNGTGPCTNVSTVQCTHGIRPVVSTQLLL
NGSLAEEEVVIRSENFTDNAKTIIVQLNESVEINCTRPNNNTRKSIHIGPGRAFYATGDIIGDIRQAHCNISRTKWNNTLKQIVKKLREQF
GNKTIVFNQSSGGDPEIVMHSFNCGGEFFYCNTTQLFNSTWNNNSTNTNNNTNTNDTITLPCRIKQIINMWQEVGKAMYAPPIRGQIR
CSSNITGLLLTRDGGNNNTNTTETFRPGGGDMRDNWRSELYKYKVVKIEPLGVAPTKAKRRVVQREKRAVGIGAMFLGFLGAAGST
MGAASITLTVQARQLLSGIVQQQNNLLRAIEAQQHLLQLTVWGIKQLQARVLAVERYLKDQQLLGIWGCSGKLICTTAVPWNTSWSNK
SLDEIWDNMTWMQWEREIDNYTGLIYTLIEESQNQQEKNEQELLELDKWASLWNWFDITNWLWYIKIFIMIVGGLIGLRIVFTVLSIVNR
VRQGYSPLSFQTRLPAPRGPDRPEGIEEEGGERDRDRSGRLVDGFLALIWVDLRSLCLFSYHRLRDLLLIVARIVELLGRRGWEALKY
WWNLLQYWSQELKNSAVSLLNATAIAVAEGTDRIIEVVQRAGRAILHIPRRIRQGLERALL
>Env_CON_C_1990s
MRVRGILRNWQQWWIWGILGFWMLMICNVVGNLWVTVYYGVPVWKEAKTTLFCASDAKAYEKEVHNVWATHACVPTDPNPQEMV
LENVTENFNMWKNDMVDQMHEDIISLWDQSLKPCVKLTPLCVTLNCTNVNVTNTNTTTYNNTMKGEIKNCSFNTTTELRDKKQKVYA
LFYRLDIVPLNENNNNSSEYRLINCNTSTITQACPKVSFDPIPIHYCAPAGYAILKCNNKTFNGTGPCNNVSTVQCTHGIKPVVSTQLLL
NGSLAEEEIIIRSENLTNNAKTIIVHLNESVEIVCTRPNNNTRKSIRIGPGQTFYATGDIIGDIRQAHCNISEEKWNKTLQRVGKKLKEHFP
NKTIKFAPSSGGDLEITTHSFNCRGEFFYCNTSKLFNSTYNPNYNSNNSNSNSTITLPCRIKQIINMWQEVGRAMYAPPIAGNITCKSNI
TGLLLTRDGGNNTNNTTETFRPGGGDMRDNWRSELYKYKVVEIKPLGIAPTKAKRRVVEREKRAVGIGAVFLGFLGAAGSTMGAASI
TLTVQARQLLSGIVQQQSNLLRAIEAQQHMLQLTVWGIKQLQTRVLAIERYLKDQQLLGIWGCSGKLICTTAVPWNSSWSNKSQEDIW
DNMTWMQWDREISNYTDTIYRLLEDSQNQQEKNEKDLLALDSWKNLWNWFDITNWLWYIKIFIMIVGGLIGLRIIFAVLSIVNRVRQGY
SPLSFQTLTPNPRGPDRLGRIEEEGGEQDRDRSIRLVSGFLALAWDDLRSLCLFSYHRLRDFILIAARAVELLGRSSLRGLQRGWEAL
KYLGSLVQYWGLELKKSAISLLDTIAIAVAEGTDRIIELIQRICRAIRNIPRRIRQGFEAALQ
>Env_CON_C_2000s
MRVRGILRNWQQWWIWGILGFWMLMICNVVGNLWVTVYYGVPVWKEAKTTLFCASDAKAYEKEVHNVWATHACVPTDPNPQEMV
LENVTENFNMWKNDMVDQMHEDIISLWDQSLKPCVKLTPLCVTLNCTNVNVTNTNTTNNTTNNNMKEEMKNCSFNTTTEIRDKKKKE
YALFYRLDIVPLNENNNSSEYRLINCNTSTITQACPKVSFDPIPIHYCAPAGYAILKCNNKTFNGTGPCNNVSTVQCTHGIKPVVSTQLL
LNGSLAEEEIIIRSENLTDNAKTIIVHLNESVEIVCTRPNNNTRKSIRIGPGQTFYATGDIIGDIRQAHCNISKSKWNKTLQRVKKKLKEHF
PNKTIKFNPSSGGDLEITTHSFNCRGEFFYCNTSKLFNSTYNSNYNNNTNSNSTITLPCRIKQIINMWQEVGRAMYAPPIAGNITCKSNI
TGLLLTRDGGNNNNNTTETFRPGGGDMRDNWRSELYKYKVVEIKPLGIAPTKAKRRVVEREKRAVGIGAVFLGFLGAAGSTMGAASI
TLTVQARQLLSGIVQQQSNLLRAIEAQQHMLQLTVWGIKQLQARVLAIERYLKDQQLLGIWGCSGKLICTTAVPWNSSWSNKSQEDIW
DNMTWMQWDREISNYTNTIYRLLEESQNQQEKNEKDLLALDSWNNLWNWFDITNWLWYIKIFIMIVGGLIGLRIIFAVLSIVNRVRQGY
SPLSFQTLTPNPRGPDRLGRIEEEGGEQDRDRSIRLVSGFLALAWDDLRSLCLFSYHRLRDFILIAARAVELLGRSSLRGLQRGWEAL
KYLGSLVQYWGLELKKSAISLLDTIAIAVAEGTDRIIELIQRICRAIRNIPRRIRQGFEAALL
>Env_CON_C_2010s
MRVRGILRNYQQWWIWGILGFWMLMICNVVGNLWVTVYYGVPVWKEAKTTLFCASDAKAYEKEVHNVWATHACVPTDPNPQEMVL
ENVTENFNMWKNDMVDQMHEDIISLWDQSLKPCVKLTPLCVTLNCTNVNVTNTNTTNNNTNNNITEEMKNCSFNATTEIRDKKKKVY
ALFYKLDIVPLNNENNNSSEYRLINCNTSAITQACPKVSFDPIPIHYCAPAGYAILKCNNKTFNGTGPCNNVSTVQCTHGIKPVVSTQLL
LNGSLAEEEIIIRSENLTNNAKTIIVHLNESVEIVCTRPNNNTRKSIRIGPGQTFYATGDIIGDIRQAHCNISESKWNKTLQRVSKKLKEHF
PNKTIKFNPSSGGDLEITTHSFNCRGEFFYCNTSKLFNGTYNGNGNTTNSTSNSTITLPCRIKQIINMWQEVGRAMYAPPIAGNITCKS
NITGLLLTRDGGNNNNNNTTETFRPGGGDMRDNWRSELYKYKVVEIKPLGIAPTKAKRRVVEREKRAVGIGAVFLGFLGAAGSTMGA
ASITLTVQARQLLSGIVQQQSNLLRAIEAQQHMLQLTVWGIKQLQARVLAIERYLKDQQLLGIWGCSGKLICTTAVPWNSSWSNKSQE
EIWDNMTWMQWDREISNYTDTIYRLLEESQNQQEKNEKDLLALDSWNNLWNWFDITNWLWYIKIFIMIVGGLIGLRIIFAVLSIVNRVR
QGYSPLSFQTLTPNPRGPDRLGRIEEEGGEQDRDRSIRLVSGFLALAWDDLRSLCLFSYHRLRDFILVTARAVELLGRSSLRGLQRG
WEALKYLGSLVQYWGLELKKSAISLLDTIAIAVAEGTDRIIELIQRICRAIRNIPRRIRQGFEAALQ
>Env_CON_D_1990s

MRVREIERNYQHLWRWGTMLLGMLMICSVTGQLWVTVYYGVPVWKEATTTLFCASDAKSYKAEAHNIWATHACVPTDPNPQEIKLE
NVTENFNMWKNNMVEQMHEDIISLWDQSLKPCVKLTPLCVTLNCTEWKNNTTTNNTNTTEDIGMKNCSFNITTEVRDKKKQVHALFY
KLDVVQIDDNSNNTSYRLINCNTSAITQACPKVTFEPIPIHYCAPAGFAILKCNDKKFNGTGPCKNVSTVQCTHGIKPVVSTQLLLNGSL
AEEEIIIRSENLTNNAKIIIVQLNESVTINCTRPYNNTRQSTHIGPGQALYTTKIIGDIRQAHCNISRAEWNKTLQQVAKKLGDLLNKTTIIFK
PSSGGDPEITTHSFNCGGEFFYCNTSGLFNSTWNNNTTNNSTGNSNETITLPCRIKQIINMWQGVGKAMYAPPIEGLIKCSSNITGLLL
TRDGGANNNSQNETFRPGGGDMRDNWRSELYKYKVVRIEPLGLAPTKAKRRVVEREKRAIGLGAMFLGFLGAAGSTMGAASLTLTV
QARQLLSGIVQQQNNLLRAIEAQQHLLQLTVWGIKQLQARILAVERYLKDQQLLGIWGCSGKHICTTNVPWNSSWSNKSLDEIWNNM
TWMEWEREIDNYTGLIYSLIEESQIQQEKNEQELLQLDKWASLWNWFSITKWLWYIKIFIMIVGGLIGLRIVFAVLSLVNRVRQGYSPLS
FQTLLPAPRGPDRPEGIEEEGGEQGRGRSIRLVNGFSALIWDDLRNLCLFSYHRLRDLILIATRIVELLGRRGWEAIKYLWNLLQYWIQ
ELKNSAISLLNTTAIAVAEGTDRVIEIVQRAVRAILNIPRRIRQGLERALL
>Env_CON_D_2000s
MRVRGIKRNYQHLWRWGTMLLGMLMICSVAGQLWVTVYYGVPVWKEATTTLFCASDAKSYKAEAHNIWATHACVPTDPNPQEIELE
NVTENFNMWKNNMVEQMHEDIISLWDQSLKPCVKLTPLCVTLNCTDWKNNNTTNTTTNNTTSEDIEMKNCSFNITTEVRDKKKQVYA
LFYKLDVVQIDNSNNTSYRLINCNTSAITQACPKVSFEPIPIHYCAPAGFAILKCNDKKFNGTGPCKNVSTVQCTHGIKPVVSTQLLLNG
SLAEEEIIIRSENLTNNAKIIIVQLNESVTINCTRPYNNTRQSIHIGPGQALYTTNIIGDIRQAHCNISGTDWNKTLQQVAKKLGDLLNKTTII
FKPSSGGDPEITTHSFNCGGEFFYCNTSGLFNSTWNNNTTTNNTTNNNETITLPCRIKQIINMWQGVGKAMYAPPIEGLIKCSSNITGL
LLTRDGGNNNNSSNETFRPGGGDMRDNWRSELYKYKVVRIEPLGLAPTKAKRRVVEREKRAIGLGAMFLGFLGAAGSTMGAASLTL
TVQARQLLSGIVQQQNNLLRAIEAQQHLLQLTVWGIKQLQARVLAVERYLKDQQLLGIWGCSGKHICTTNVPWNSSWSNKSLDEIWN
NMTWMEWEREIDNYTGLIYSLIEESQIQQEKNEQELLELDKWASLWNWFSITNWLWYIKIFIMIVGGLIGLRIVFAVLSLVNRVRQGYSP
LSFQTLLPAPRGPDRPEGIEEEGGEQGRGRSIRLVNGFSALIWDDLRNLCLFSYHRLRDLILIAARIVELLGRRGWEALKYLWNLLQYW
IQELKNSAISLLNTTAIAVAEGTDRVIEIVQRAGRAILNIPRRIRQGLERALL
>Env_CON_F1_2000s
MRVRGMQRNWQHLGKWGLLFLGILIICNAAENLWVTVYYGVPVWKEATTTLFCASDAKAYEKEVHNVWATHACVPTDPNPQEIVLE
NVTENFDMWKNNMVEQMHTDIISLWDQSLKPCVKLTPLCVTLNCTNANNTSTNTTTQNSTLKEEPGAIQNCSFNMTTEVRDKKQKV
HALFYRLDIVPINNNSSSSEYRLINCNTSTITQACPKVSWDPIPIHYCAPAGYAILKCNDKTFNGTGPCKNVSTVQCTHGIKPVVSTQLL
LNGSLAEEDIIIRSQNISDNAKTIIVHLNESVQINCTRPNNNTRKSIHIGPGQAFYATGDIIGDIRKAHCNISGAQWNKTLERVKAKLKSYF
PNKTIKFNSSSGGDLEITMHSFNCRGEFFYCNTSGLFNDTTNETNDTITLPCRIKQIVNMWQEVGRAMYAAPIAGNITCNSNITGLLLTR
DGGNNNNNTTETFRPGGGNMKDNWRSELYKYKVVEIEPLGVAPTRAKRQVVQREKRAVGIGALFLGFLGAAGSTMGAASITLTVQA
RQLLSGIVQQQNNLLRAIEAQQHLLQLTVWGIKQLQARVLAVERYLKDQQLLGLWGCSGKLICTTNVPWNSSWSNKSQEEIWDNMT
WMEWEKEISNYSNEIYRLIEESQNQQEKNEQELLALDKWASLWNWFDISNWLWYIKIFIMIVGGLIGLRIVFAVLSIVNRVRKGYSPLSL
QTLIPSPREPDRPEGIEEGGGEQGRDRSVRLVNGFLALVWDDLRNLCLFSYRHLRDFILIAARIVNRGLRRGWEALKYLGNLTQYWS
QELKNSAISLLNTTAIVVAEGTDRVIEALQRAGRAILNIPRRIRQGLERALL
>Env_CON_F1_2010s
MRVRGMQRNWQHLGKWGLLFLGILIICNATDNLWVTVYYGVPVWKEATTTLFCASDAKAYEKEVHNVWATHACVPTDPNPQEVNLE
NVTENFNMWKNNMVEQMHTDIISLWDQSLKPCVKLTPLCVTLNCTNANATNTTNTNQNSTLKEEPGAIQNCSFNMTTEVRDKQKKV
HALFYRLDIVPINNESSNNSGEYRLINCNTSTITQACPKVSWDPIPIHYCAPAGYAILKCNDKKFNGTGPCKNVSTVQCTHGIKPVVSTQ
LLLNGSLAEEDIIIRSQNISDNAKTIIVHLNESVQINCTRPNNNTRKSIHLGPGRAFYATGDIIGDIRKAHCNVSGTQWNKTLEQVKAKLK
SHFPNKTIKFNSSSGGDLEITMHSFNCRGEFFYCNTSGLFNDTNITSNDTITLPCRIKQIINMWQEVGRAMYAAPIAGNITCNSNITGLLL
TRDGGNNNETNTTETFRPGGGNMKDNWRSELYKYKVVEIEPLGVAPTRAKRQVVKREKRAVGIGALFLGFLGAAGSTMGAASITLTV
QARQLLSGIVQQQNNLLRAIEAQQHMLQLTVWGIKQLQARVLAVERYLKDQQLLGLWGCSGKLICTTNVPWNSSWSNKSQEQIWDN
MTWMQWEKEISNYSQEIYRLIEESQNQQEKNEQELLALDKWASLWNWFDISNWLWYIKIFIMIVGGLIGLRIVFAVLSIVNRVRKGYSP
LSLQTHIPSPREPDRPEGIEEGGGEQGRDRSVRLVNGFLALVWDDLRNLCLFSYRHLRDFILIAARIVDRGLRRGWEALKYLGNLTQY
WSQELKNSAISLLNTTAIVVAEGTDRVIEALQRAGRAVLNIPRRIRQGLERALL
>Env_CON_G_1990s
MRVKGIQRNWQHLWTWGTLILGLVIICSASNNLWVTVYYGVPVWEDADTPLFCASDAKAYSTEKHNVWATHACVPTDPNPQEITLEN
VTENFNMWKNNMVEQMHEDIISLWDESLKPCVKLTPLCVTLNCTNVNNNSVTNNNSTVVTEEAEIKNCSFNITTEIKDRKKKEYALFY
KLDVVPINDNNNSASNYRLINCNVSTIKQACPKVTFDPIPIHYCAPAGFAILKCRDKNFNGTGPCKNVSTVQCTHGIKPVVSTQLLLNGS
LAEEEIVIRSENITDNAKVIIVQLNETIEINCTRPNNNTRKSIRIGPGQAFYATGDIIGDIRQAHCNVSGTKWNEMLQKVKAQLKKIFNNKTI
TFNSSTGGDLEITTHSFNCRGEFFYCNTSGLFNNSNSTISNDTITLPCKIKQIVRMWQRVGQAMYAPPIAGNITCRSNITGLLLTRDGG
NNNSTNETFRPGGGDMRDNWRSELYKYKVVKIKPLGIAPTRARRRVVEREKRAVGLGAVLLGFLGAAGSTMGAASITLTVQVRQLLS
GIVQQQSNLLRAIEAQQHLLQLTVWGIKQLQARVLAVERYLKDQQLLGIWGCSGKLICTTNVPWNTSWSNKSYEEIWDNMTWIEWER
EISNYTQQIYSLIEESQNQQEKNEQDLLALDKWASLWNWFDITNWLWYIRIFIMIVGGLIGLRIVFAVLSIVNRVRQGYSPLSFQTLTHH
QREPDRPEGIEEGGGEQDKDRSIRLVSGFLALAWDDLRSLCLFSYHRLRDFILIAARTVELLGRSSLKGLRLGWEGLKYLWNLLLYWG
RELKNSAINLLDTIAIAVANWTDRVIEVAQRAGRAILNIPRRIRQGLERALL
>Env_CON_G_2000s
MRVKGIQRNWQHLWKWGTLILGLVIICSASNNLWVTVYYGVPVWEDADTPLFCASDAKAYSTEKHNVWATHACVPTDPNPQEIPLE
NVTENFNMWKNNMVEQMHEDIISLWDESLKPCVKLTPLCVTLNCTNVTNNSTNTNTNNNSTVEEKEELKNCSFNITTEIRDKKKKEYA
LFYKLDVVPINDNNNSSSYRLINCNVSTIKQACPKVSFDPIPIHYCAPAGFAILKCRDKNFNGTGPCKNVSTVQCTHGIKPVVSTQLLLN
GSLAEEDIMIRSENITDNAKTIIVQLNKTIEINCTRPNNNTRKSIRIGPGQAFYATGDIIGDIRQAHCNVSGTKWNNMLQNVKAKLKEIFNN
KNITFNSSSGGDLEITTHSFNCRGEFFYCNTSGLFNSSLLNNNSNSTNDTITLPCKIKQIVRMWQRVGQAMYAPPIAGNITCRSNITGLL
LTRDGGNNNNNTTETFRPAGGDMRDNWRSELYKYKVVKIKPLGVAPTKARRRVVGREKRAVGLGAVFLGFLGAAGSTMGAASITLT
VQVRQLLSGIVQQQSNLLRAIEAQQHLLQLTVWGIKQLQARVLAVERYLKDQQLLGIWGCSGKLICTTNVPWNTSWSNKSYEEIWDN
MTWIQWEREISNYTQQIYSLIEESQNQQEKNEQDLLALDKWASLWNWFDISNWLWYIKIFIMIVGGLIGLRIVFAVLSIVNRVRQGYSPL
SFQTLTHHQREPDRPERIEEGGGEQDRDRSIRLVSGFLALAWDDLRSLCLFSYHRLRDFILIAARTVELLGRSSLKGLRLGWEGLKYL
WNLLLYWGQELKNSAINLLDTVAIAVANWTDRVIEVAQRAGRAILNIPRRIRQGLERALL
>Env_CON_G_2010s
MRVKGIQRNWQHLWTWWTLILGLVIICSASENLWVTVYYGVPVWEDADTPLFCASDAKAYSTEKHNVWATHACVPTDPNPQEIPLE
NVTENFNMWKNNMVEQMHEDIISLWDESLKPCVKLTPLCVTLNCTNVNNTNNNNTNNNNNTTLDNKEELKNCSFNITTEIRDKKKKE
YALFYKLDVVPIGNNDNSSYRLINCNVSTIKQACPKVSFDPIPIHYCAPAGFAILKCNDKNFNGTGPCKNVSTVQCTHGIKPVVSTQLLL
NGSLAEEKIVIRSENITDNTKVIIVQLNKTIEINCTRPNNNTRKSIRIGPGQAFYATGDIIGDIRQAHCNVSRTKWNEMLQKVKEQLKKIYK

NKNITFNSSTGGDLEITTHSFNCRGEFFYCNTSKLFNNSLINNNNSTSNDTITLPCRIKQIVRMWQRVGQAMYAPPIAGNITCISNITGLL
LTRDGGNNNNTNNETFRPGGGDMRDNWRSELYKYKIVKIKPLGIAPTKARRRVVEREKRAVGLGAVFLGFLGAAGSTMGAASITLTV
QVRQLLSGIVQQQSNLLRAIEAQQHLLQLTVWGIKQLQARVLAVERYLKDQQLLGIWGCSGKLICTTNVPWNTSWSNKSYEEIWDNM
TWLQWEREINNYTQQIYSLIEQSQIQQEKNEQDLLALDKWASLWNWFDISNWLWYIKIFIMIVGGLIGLRIVFAVLSIVNRVRQGYSPLS
FQTLTHHQREPDRPERTEEGGGEQDKDRSIRLVSGFLALAWDDLRSLCLFSYHRLRDFILIAARTVELLGRSSLQGLRLGWEGLKYL
WNLLLYWGQELKNSAINLLDTIAIAVANWTDRVIEVAQRACRAILNIPRRIRQGLERALL
>Env_CON_01_AE_1990s
MRVKETQMNWPNLWKWGTLILGLVIICSASDNLWVTVYYGVPVWRDADTTLFCASDAKAHETEVHNVWATHACVPTDPNPQEIHLE
NVTENFNMWKNNMVEQMQEDVISLWDQSLKPCVKLTPLCVTLNCTNANLTNVNNTTNVSNIIGNITDEVRNCSFNMTTELRDKKQKV
HALFYKLDIVQIENNNNSSEYRLINCNTSVIKQACPKISFDPIPIHYCTPAGYAILKCNDKNFNGTGPCKNVSSVQCTHGIKPVVSTQLLL
NGSLAEEEIIIRSENLTNNAKTIIVHLNKSVEINCTRPSNNTRTSITIGPGQVFYRTGDIIGDIRKAYCEINGTKWNEVLKQVTGKLKEHFN
NKTIIFQPPSGGDLEITMHHFNCRGEFFYCNTTKLFNNTCIGNETMEGCNGTIILPCKIKQIINMWQGVGQAMYAPPISGRINCVSNITGI
LLTRDGGNNNTTNETFRPGGGNIKDNWRSELYKYKVVQIEPLGIAPTRAKRRVVEREKRAVGIGAMIFGFLGAAGSTMGAASITLTVQ
ARQLLSGIVQQQSNLLRAIEAQQHLLQLTVWGIKQLQARVLAVERYLKDQKFLGLWGCSGKIICTTAVPWNSTWSNKSYEEIWNNMT
WIEWEREISNYTNQIYEILTESQNQQDRNEKDLLELDKWASLWNWFDITNWLWYIKIFIMIVGGLIGLRIIFAVLSIVNRVRQGYSPLSFQ
TPTHHQREPDRPERIEEGGGEQGRDRSVRLVSGFLALAWDDLRSLCLFSYHRLRDFILIAARTVELLGHSSLKGLRRGWEGLKYLGN
LLLYWGQELKISAISLLDATAIAVAGWTDRVIEVAQRAWRAILHIPRRIRQGLERALL
>Env_CON_01_AE_2000s
MRVKETQMNWPNLWKWGTLILGLVIICSASDNLWVTVYYGVPVWRDADTTLFCASDAKAHETEVHNVWATHACVPTDPNPQEIHLE
NVTENFNMWKNNMVEQMQEDVISLWDQSLKPCVKLTPLCVTLNCTNANLTNTNTTTTNSNNIGNITDEVRNCSFNMTTELRDKKQKV
HALFYKLDIVPIDNNNNSSEYRLINCNTSVIKQACPKISFDPIPIHYCTPAGYAILKCNDKNFNGTGPCKNVSSVQCTHGIKPVVSTQLLL
NGSLAEEEIIIRSENLTNNAKTIIVHLNKSVEINCTRPSNNTRTSITIGPGQVFYRTGDIIGDIRKAYCEINGTKWNETLKQVAEKLKEHFN
NKTIIFQPPSGGDLEITMHHFNCRGEFFYCNTTKLFNSTWIGNETTEGCNGTIILPCRIKQIINMWQGVGQAMYAPPISGIINCVSNITGIL
LTRDGGNNNNTNETFRPGGGNIKDNWRSELYKYKVVQIEPLGIAPTRAKRRVVEREKRAVGIGAMIFGFLGAAGSTMGAASITLTVQA
RQLLSGIVQQQSNLLRAIEAQQHLLQLTVWGIKQLQARVLAVERYLKDQKFLGLWGCSGKIICTTAVPWNSTWSNKSYEEIWNNMTW
IEWEREISNYTNQIYEILTESQNQQDRNEKDLLELDKWASLWNWFDITNWLWYIKIFIMIVGGLIGLRIIFAVLSIVNRVRQGYSPLSFQT
PTHHQREPDRPERIEEEGGEQGRDRSVRLVSGFLALAWDDLRSLCLFSYHRLRDFILIAARTVELLGHSSLKGLRRGWEGLKYLGNL
LLYWGQELKISAISLLDATAIAVAGWTDRVIEVAQRAWRAILHIPRRIRQGLERALL
>Env_CON_01_AE_2010s
MRVKETQMNWPNLWKWGTLILGLVIICSASDNLWVTVYYGVPVWRDADTTLFCASDAKAHETEVHNVWATHACVPTDPNPQEIHLE
NVTENFNMWKNNMVEQMQEDVISLWDQSLKPCVKLTPLCVTLNCTNANLTNTNTTNTNNTSNIGNITDEVRNCTFNMTTEIRDKKQK
VYALFYKLDIVPIDNNNNSSEYRLINCNTSVIKQACPKISFDPIPIHYCTPAGYAILKCNDKNFNGTGPCKNVSSVQCTHGIKPVVSTQLL
LNGSLAEEEIIIRSENLTNNAKTIIVHLNKSVEINCTRPSNNTRTSIRIGPGQVFYRTGDIIGDIRKAYCEINGTKWNETLKQVAEKLKEHF
NNKTIIFQPPSGGDLEITMHHFNCRGEFFYCNTTKLFNSTCNGNGNTTEGCNGTIILPCRIKQIINMWQGVGQAMYAPPISGIINCVSNI
TGILLTRDGGNNNNTNETFRPGGGNIKDNWRSELYKYKVVQIEPLGIAPTRAKRRVVEREKRAVGIGAMIFGFLGAAGSTMGAASITLT
VQARQLLSGIVQQQSNLLRAIEAQQHLLQLTVWGIKQLQARVLAVERYLKDQKFLGLWGCSGKIICTTAVPWNSTWSNKSYEEIWNN
MTWIEWEREISNYTNQIYEILTESQNQQDKNEKDLLELDKWASLWNWFDITNWLWYIKIFIMIVGGLIGLRIIFAVLSIVNRVRQGYSPLS
FQTPTHHQREPDRPERIEEGGGEQGRDRSVRLVSGFLALAWDDLRSLCLFSYHRLRDFILIAARTVELLGHSSLKGLRRGWEGLKYL
GNLLLYWGQELKTSAISLLDATAIAVAGWTDRVIEVAQRAWRAILHIPRRIRQGLERALL
>Env_CON_02_AG_1990s
MRVMGIQKNYPLLWRWGMIIFWIMIICNAENLWVTVYYGVPVWRDAETTLFCASDAKAYDTEAHNVWATHACVPTDPNPQEIHLENV
TENFNMWKNNMVEQMHEDIISLWDQSLKPCVKLTPLCVTLNCHDVNSTSNNTSNNISSNMQGEIKNCSFNMTTELRDKKQKVYALFY
RLDVVQINNSNNSSQYRLINCNTSAITQACPKVSFEPIPIHYCAPAGFAILKCNDKEFNGTGPCKNVSTVQCTHGIKPVVSTQLLLNGSL
AEEEIVIRSENITNNAKTIIVQLNEPVKINCTRPGNNTRKSVRIGPGQTFYATGDIIGDIRQAHCNVSRTEWNNTLQKVATQLRKYFNNTT
IIFANSSGGDLEITTHSFNCGGEFFYCNTSELFNSTWNNNSTNSSNSTESNDTITLQCRIKQIVNMWQRVGQAMYAPPIQGVIRCESNI
TGLLLTRDGGNNNSTNETFRPGGGDMRDNWRSELYKYKVVKIEPLGVAPTHAKRRVVEREKRAVGLGAVFLGFLGAAGSTMGAASI
TLTVQARQLLSGIVQQQSNLLKAIEAQQHLLKLTVWGIKQLQARVLALERYLKDQQLLGIWGCSGKLICTTTVPWNSSWSNKTYNDIW
DNMTWLQWDKEISNYTNIIYNLIEESQNQQEKNEQDLLALDKWASLWNWFDITNWLWYIKIFIMIVGGLIGLRIVFAVLTIINRVRQGYSP
LSFQTLTHHQREPDRPERIEEGGGEQDRDRSVRLVSGFLALAWDDLRSLCLFSYHRLRDFVLIAARTVELLGHSSLKGLRLGWEALK
YLWNLLSYWGQELKNSAINLLDTIAIAVANWTDRVIEIGQRAGRAILNIPRRIRQGLERALL
>Env_CON_02_AG_2000s
MRVMGIQRNYPLLWRWGMIIFWIMIICNAENLWVTVYYGVPVWRDAETTLFCASDAKAYDTEAHNVWATHACVPTDPNPQEIHLENV
TENFNMWKNNMVEQMHEDIISLWDQSLKPCVKLTPLCVTLNCSDVNNSSSNSNNSNISEEMKGEIKNCSFNMTTELRDKKQKVYALF
YRLDVVQINENNSNSSQYRLINCNTSAITQACPKVSFEPIPIHYCAPAGFAILKCNDKEFNGTGPCKNVSTVQCTHGIKPVVSTQLLLNG
SLAEEEIVIRSENITNNAKTIIVQLNEPVKINCTRPNNNTRKSVRIGPGQTFYATGDIIGDIRQAHCNVSKTEWNKTLQQVATQLRKYFNN
TTIIFANSSGGDLEITTHSFNCGGEFFYCNTSGLFNSTWNNNTTNNNNSSNSNDTITLQCRIKQIVNMWQRVGQAMYAPPIQGEIRCE
SNITGLLLTRDGGNNNSTNETFRPGGGDMRDNWRSELYKYKVVKIEPLGVAPTHAKRRVVEREKRAVGLGAVFLGFLGAAGSTMGA
ASITLTVQARQLLSGIVQQQSNLLRAIEAQQHLLKLTVWGIKQLQARVLALERYLRDQQLLGIWGCSGKLICTTTVPWNSSWSNKTYN
DIWDNMTWLQWDKEISNYTDIIYNLIEESQNQQEKNEQDLLALDKWASLWNWFDITNWLWYIKIFIMIVGGLIGLRIVFAVLNIINRVRQ
GYSPLSFQTLTHHQREPDRPERIEEGGGEQDRDRSVRLVSGFLALAWDDLRSLCLFSYHRLRDFVLIAARTVELLGHSSLKGLRLGW
EALKYLWNLLSYWGQELKNSAINLLDTIAIAVANWTDRVIEIGQRAGRAILNIPRRIRQGLERALL
>Env_CON_02_AG_2010s
MRVMGIQKNYPLLWRWGMIIFWIIIICNAENLWVTVYYGVPVWRDAETTLFCASDAKAYDTEVHNVWATHACVPTDPNPQEIHLENVT
ENFNMWKNNMVEQMHEDIISLWDQSLKPCVKLTPLCVTLNCSNANNSTSNNNNSNISSDMEGEIKNCSFNMTTELRDKKQKVYALFY
RLDVVQINENNSSQYRLINCNTSAITQACPKVSFEPIPIHYCAPAGFAILKCNDKEFNGTGPCKNVSTVQCTHGIKPVVSTQLLLNGSLA
EEEIVIRSENITNNAKTIIVQLNEPVKINCTRPNNNTRKSVRIGPGQTFYATGDIIGDIRQAHCNVSRTEWNNTLRKVAKQLRKHFNNKTII
FTNSSGGDLEITTHSFNCGGEFFYCNTSGLFNSTWNNNNTTTNNNNTESNDTITLQCRIKQIVNMWQRVGQAMYAPPIPGVIRCESNI
TGLLLTRDGGNNNSTNETFRPGGGDMRDNWRSELYKYKVVKIEPLGVAPTHAKRRVVEREKRAVGLGAVFLGFLGAAGSTMGAASI
TLTVQARQLLSGIVQQQSNLLKAIEAQQHLLRLTVWGIKQLQARVLALERYLRDQQLLGIWGCSGKLICTTTVPWNSSWSNKTYNDIW
DNMTWLQWDKEISNYTDIIYNLIEESQNQQEKNEQDLLALDKWASLWNWFDITNWLWYIKIFIMIVGGLIGLRIVFAVLAIINRVRQGYS

PLSFQTLTHHQREPDRPERIEEGGGEQDRDRSVRLVSGFLALAWDDLRSLCLFSYHRLRDLALIAARTVELLGHSSLKGLRLGWEAL
KYLWNLLSYWGQELKNSAINLLDTIAIAVANWTDRIIEIGQRAGRAILNIPRRIRQGLERALL
>Env_CON_07_BC_2000s
MRVTGIRKNYRHLWRWGTMLLGMLMICSAVGNLWVTVYYGVPVWKEATTTLFCASDAKAYDTEVHNVWATHACVPTDPNPQEMVL
ENVTENFNMWKNEMVNQMHEDVISLWDQSLKPCVKLTPLCVTLECRNVSSNSNSNNNNTYNESMKEMKNCSFNATTVVRDRKQKV
YALFYRLDIVPLTEKNSENSSSYYRLINCNTSAITQACPKVTFDPIPIHYCTPAGYAILKCNDKTFNGTGPCHNVSTVQCTHGIKPVVST
QLLLNGSLAEEEIIIRSENLTNNVKTIIVHLNQSVEIVCTRPGNNTRKSIRIGPGQTFYATGDIIGDIRQAHCNISEDKWNETLQRVSKKLA
EHFPNKTIKFASSSGGDLEITTHSFNCRGEFFYCNTSGLFNGTYMPNGTSTGSNSSSTITIPCRIKQIINMWQEVGRAMYAPPIAGNITC
KSNITGLLLVRDGGTNETNTTETFRPGGGDMRDNWRSELYKYKVVEIKPLGVAPTAAKRRVVEREKRAVGIGAVFLGFLGAAGSTMG
AASITLTVQARQLLSGIVQQQSNLLRAIEAQQHLLQLTVWGIKQLQTRVLAIERYLKDQQLLGIWGCSGKLICTTAVPWNSSWSNKSQT
EIWDNMTWMQWDKEISNYTNTIYRLLEDSQNQQERNEKDLLALDSWKNLWNWFDITNWLWYIKIFIMIVGGLIGLRIIFAVLSIVNRVR
QGYSPLSFQTLTPNPGGPDRLGRIEEEGGEQDKDRSIRLVNGFLALAWDDLRNLCLFSYHRLRDFILVTARVVELLGRNSLRGLQRG
WEALKYLGSLVQYWGQELKKSAISLVDTIAIAVAEGTDRIIELVQGLCRAIYNIPRRIRQGFEAALQ
>Env_CON_07_BC_2010s
MRVTGIRKNYQHLWRWGTMLLGMLMICSAAGNLWVTVYYGVPVWKEATTTLFCASDAKAYDTEVHNVWATHACVPTDPNPQEIVL
ENVTENFNMWKNEMVDQMHEDVISLWDQSLKPCVKLTPLCVTLECTNVSSNSNSSNSSNINVESNEEMKNCSFNTTTEVRDKKQKV
YALFYKLDIVPLDSENSSSNSSEYRLINCNTSAITQACPKVTFDPIPIHYCTPAGYALLKCNDKKFNGTGPCHNVSTVQCTHGIKPVVST
QLLLNGSLAEEEIIIRSENLTNNAKTIIVHLNKSVEIVCTRPGNNTRKSIRIGPGQTFYATGDIIGDIRQAHCNISKGDWNETLQKVSKKLA
EHFPNKTIKFASSSGGDLEITTHSFNCRGEFFYCNTSGLFNGTYNPNGTSTDSNSNSTITIPCRIKQIINMWQEVGRAMYAPPIAGNITC
KSNITGLLLVRDGGNSNTNTTEIFRPGGGDMRDNWRSELYKYKVVEIKPLGVAPTEAKRRVVEREKRAVGLGAVFLGFLGAAGSTMG
AASITLTVQARQLLSGIVQQQSNLLKAIEAQQHLLQLTVWGIKQLQTRVLAIERYLKDQQLLGIWGCSGKLICTTAVPWNSSWSNKSQT
EIWDNMTWMQWDKEISNYTNTIYRLLEESQNQQEKNEKDLLALDSWKNLWNWFDITNWLWYIKIFIMIVGGLIGLRIIFAVLSIVNRVRQ
GYSPLSLQTLIPNPGGPDRLGRIEEEGGEQDRDRSIRLVNGFLALAWDDLRNLCLFSYHRLRDFILVTARVVELLGRNSLKGLQRGWE
ALKYLGSLVQYWGQELKKSAISLLDTIAIAVAEGTDRIIELVQRFCRAIYNIPRRIRQGFEAALQ

File S5. Gag consensus sequences for each subtype/CRF and sampling period. Suffixes in the consensus sequence name indicate the subtype/CRF and decade during which sequences were sampled.

>Gag_CON_A1_1990s
MGARASVLSGGKLDAWEKIRLRPGGKKKYRLKHLVWASRELERFALNPSLLETTEGCQQIMEQLQPALKTGTEELRSLFNTVATLYC
VHQRIDVKDTKEALDKIEEIQNKSKQKTQQAAADTGNSSKVSQNYPIVQNAQGQMIHQSLSPRTLNAWVKVIEEKAFSPEVIPMFSAL
SEGATPQDLNMMLNIVGGHQAAMQMLKDTINEEAAEWDRLHPVHAGPIPPGQMREPRGSDIAGTTSTPQEQIGWMTSNPPIPVGDI
YKRWIILGLNKIVRMYSPVSILDIKQGPKEPFRDYVDRFFKTLRAEQATQEVKGWMTETLLVQNANPDCKSILRALGTGATLEEMMTA
CQGVGGPGHKARVLAEAMSQVQHTNIMMQRGNFRGQKRIKCFNCGKEGHLARNCRAPRKKGCWKCGKEGHQMKDCTERQANFL
GKIWPSSKGRPGNFPQSRLEPTAPPAEIFGMGEEIASPPKQEQKDREQAPLVSLKSLFGNDPLSQ
>Gag_CON_A1_2000s
MGARASVLSGGKLDAWEKIRLRPGGKKKYRLKHLVWASRELERFALNPSLLETAEGCQQIMEQLQPALKTGTEELKSLFNTVATLYC
VHQRIDVKDTKEALDKIEEIQNKSKQKTQQAAADTGNSSKVSQNYPIVQNAQGQMIHQALSPRTLNAWVKVIEEKAFSPEVIPMFSAL
SEGATPQDLNMMLNIVGGHQAAMQMLKDTINEEAAEWDRLHPVHAGPIPPGQMREPRGSDIAGTTSTPQEQIGWMTGNPPIPVGDI
YKRWIILGLNKIVRMYSPVSILDIKQGPKEPFRDYVDRFFKTLRAEQATQEVKGWMTETLLVQNANPDCKSILRALGTGATLEEMMTA
CQGVGGPGHKARVLAEAMSQVQQTNIMMQRGNFRGQKRIKCFNCGKEGHLARNCRAPRKKGCWKCGKEGHQMKDCTERQANFL
GKIWPSSKGRPGNFPQSRPEPTAPPAEIFGMGEEIASPPKQEQKDREQAPPLVSLKSLFGNDPLSQ
>Gag_CON_A1_2010s
MGARASVLSGGKLDAWEKIRLRPGGKKKYRLKHLVWASRELERFALNPSLLETAEGCQQIMEQLQPALKTGTEELKSLFNTVATLYC
VHQRIDVKDTKEALDKIEEIQNKSKQKTQQAAADTGNSSKVSQNYPIVQNAQGQMIHQALSPRTLNAWVKVIEEKAFSPEVIPMFSAL
SEGATPQDLNMMLNIVGGHQAAMQMLKDTINEEAAEWDRLHPVHAGPIPPGQMREPRGSDIAGTTSTPQEQIGWMTGNPPIPVGDI
YKRWIILGLNKIVRMYSPVSILDIKQGPKEPFRDYVDRFFKTLRAEQATQEVKGWMTETLLVQNANPDCKSILRALGTGATLEEMMTA
CQGVGGPGHKARVLAEAMSQVQQTNIMMQRGNFRGQKRIKCFNCGKEGHLARNCRAPRKKGCWKCGKEGHQMKDCTERQANFL
GKIWPSSKGRPGNFPQSRPEPTAPPAEIFGMGEEIASPPKQEQKDREQNPPLVSLKSLFGNDPLSQ
>Gag_CON_A6_2000s
MGARASVLSGGKLDAWEKIRLRPGGKKKYRLKHLVWASRELERFALNPGLLETSEGCQQILEQLQPTLKTGSEELKSLYNTVATLYC
VHQRIEIKDTKEALDKIEEIQNKSKQKTQQAATGTGSSSKVSQNYPIVQNAQGQMIHQSMSPRTLNAWVKVIEEKAFSPEVIPMFSALS
EGATPQDLNMMLNIVGGHQAAMQMLKDTINEEAAEWDRLHPAQAGPFPPGQMREPRGSDIAGTTSTLQEQIGWMTSNPPIPVGDIY
KRWIILGLNKIVRMYSPVSILDIRQGPKEPFRDYVDRFFKTLRAEQATQDVKNWMTETLLVQNANPDCKTILRALGPGATLEEMMTAC
QGVGGPGHKARVLAEAMSQVQNANIMMQKSNFRGPKRIKCFNCGKEGHLARNCRAPRKKGCWKCGKEGHQMKDCTERQANFLG
RIWPSSKGRPGNFPQSRPEPSAPPAENFGMGEEITPSLKQEQKDREQPPPSISLKSLFGNDPLSQ
>Gag_CON_A6_2010s
MGARASVLSGGKLDAWEKIRLRPGGKKKYRIKHLVWASRELERFALNPGLLETSEGCQQILEQLQPALKTGSEELKSLYNTVATLYCV
HQRIEIKDTKEALDKIEEIQNKSKQKTQQAATGTGSSSKVSQNYPIVQNAQGQMVHQSMSPRTLNAWVKVIEEKAFSPEVIPMFSALS
EGATPQDLNMMLNIVGGHQAAMQMLKDTINEEAAEWDRLHPAQAGPFPPGQMREPRGSDIAGTTSTLQEQIGWMTSNPPIPVGDIY
KRWIILGLNKIVRMYSPVSILDIRQGPKEPFRDYVDRFFKTLRAEQATQDVKNWMTETLLVQNANPDCKTILRALGPGATLEEMMTAC
QGVGGPGHKARVLAEAMSQVQNANIMMQKSNFRGPKRIKCFNCGKEGHLARNCRAPRKKGCWKCGKEGHQMKDCTERQANFLG
RIWPSSKGRPGNFPQSRPEPSAPPAENFGMGEEITPSLKQEQKDREQRPPSISLKSLFGNDPLSQ
>Gag_CON_B_1980s
MGARASVLSGGELDRWEKIRLRPGGKKKYKLKHIVWASRELERFAVNPGLLETSEGCRQILGQLQPSLQTGSEELRSLYNTVATLYC
VHQKIEVKDTKEALEKIEEEQNKSKKKAQQAAADTGNSSQVSQNYPIVQNLQGQMVHQAISPRTLNAWVKVVEEKAFSPEVIPMFSA
LSEGATPQDLNTMLNTVGGHQAAMQMLKETINEEAAEWDRLHPVHAGPIAPGQMREPRGSDIAGTTSTLQEQIGWMTNNPPIPVGEI
YKRWIILGLNKIVRMYSPTSILDIRQGPKEPFRDYVDRFYKTLRAEQASQEVKNWMTETLLVQNANPDCKTILKALGPAATLEEMMTAC
QGVGGPGHKARVLAEAMSQVTNSATIMMQRGNFRNQRKTVKCFNCGKEGHIAKNCRAPRKKGCWKCGKEGHQMKDCTERQANF
LGKIWPSHKGRPGNFLQSRPEPTAPPEESFRFGEETTTPSQKQEPIDKELYPLASLRSLFGNDPSSQ
>Gag_CON_B_1990s
MGARASVLSGGELDRWEKIRLRPGGKKKYKLKHIVWASRELERFAVNPGLLETSEGCRQILGQLQPSLQTGSEELKSLYNTVATLYC
VHQRIEVKDTKEALDKIEEEQNKSKKKAQQAAADTGNSSQVSQNYPIVQNLQGQMVHQAISPRTLNAWVKVVEEKAFSPEVIPMFSA
LSEGATPQDLNTMLNTVGGHQAAMQMLKETINEEAAEWDRLHPVHAGPIAPGQMREPRGSDIAGTTSTLQEQIGWMTNNPPIPVGEI
YKRWIILGLNKIVRMYSPTSILDIRQGPKEPFRDYVDRFYKTLRAEQASQEVKNWMTETLLVQNANPDCKTILKALGPAATLEEMMTAC
QGVGGPGHKARVLAEAMSQVTNSATIMMQRGNFRNQRKTVKCFNCGKEGHIAKNCRAPRKKGCWKCGKEGHQMKDCTERQANF
LGKIWPSHKGRPGNFLQSRPEPTAPPEESFRFGEETTTPSQKQEPIDKELYPLASLRSLFGNDPSSQ
>Gag_CON_B_2000s
MGARASVLSGGELDRWEKIRLRPGGKKKYRLKHIVWASRELERFAVNPGLLETSEGCRQILGQLQPSLQTGSEELKSLYNTVATLYC
VHQRIEVKDTKEALDKIEEEQNKSKKKAQQAAADTGNSSQVSQNYPIVQNLQGQMVHQAISPRTLNAWVKVVEEKAFSPEVIPMFSA
LSEGATPQDLNTMLNTVGGHQAAMQMLKETINEEAAEWDRLHPVHAGPIAPGQMREPRGSDIAGTTSTLQEQIGWMTNNPPIPVGEI
YKRWIILGLNKIVRMYSPTSILDIRQGPKEPFRDYVDRFYKTLRAEQASQEVKNWMTETLLVQNANPDCKTILKALGPAATLEEMMTAC
QGVGGPGHKARVLAEAMSQVTNSATIMMQRGNFRNQRKTVKCFNCGKEGHIAKNCRAPRKKGCWKCGKEGHQMKDCTERQANF
LGKIWPSHKGRPGNFLQSRPEPTAPPEESFRFGEETTTPSQKQEPIDKELYPLASLKSLFGNDPSSQ
>Gag_CON_B_2010s
MGARASVLSGGELDRWEKIRLRPGGKKKYRLKHIVWASRELERFAVNPGLLETSEGCRQILGQLQPSLQTGSEELKSLYNTVAVLYC
VHQRIEVKDTKEALDKIEEEQNKSKKKAQQAAADTGNSSQVSQNYPIVQNLQGQMVHQAISPRTLNAWVKVVEEKAFSPEVIPMFSA
LSEGATPQDLNTMLNTVGGHQAAMQMLKETINEEAAEWDRLHPVHAGPIAPGQMREPRGSDIAGTTSTLQEQIGWMTNNPPIPVGEI
YKRWIILGLNKIVRMYSPTSILDIRQGPKEPFRDYVDRFYKTLRAEQASQEVKNWMTETLLVQNANPDCKTILKALGPAATLEEMMTAC
QGVGGPGHKARVLAEAMSQVTNSATIMMQRGNFRNQRKTVKCFNCGKEGHIARNCRAPRKKGCWKCGKEGHQMKDCTERQANF
LGKIWPSHKGRPGNFLQSRPEPTAPPEESFRFGEETTTPSQKQEPIDKELYPLASLRSLFGNDPSSQ
>Gag_CON_C_1990s

MGARASILRGGKLDKWEKIRLRPGGKKHYMLKHLVWASRELERFALNPGLLETSEGCKQIIKQLQPALQTGTEELRSLYNTVATLYCV
HEGIEVRDTKEALDKIEEEQNKSQQKTQQAKAADGKVSQNYPIVQNLQGQMVHQAISPRTLNAWVKVIEEKAFSPEVIPMFTALSEGA
TPQDLNTMLNTVGGHQAAMQMLKDTINEEAAEWDRLHPVHAGPIAPGQMREPRGSDIAGTTSTLQEQIAWMTSNPPIPVGDIYKRWI
ILGLNKIVRMYSPVSILDIKQGPKEPFRDYVDRFFKTLRAEQATQDVKNWMTDTLLVQNANPDCKTILRALGPGATLEEMMTACQGVG
GPSHKARVLAEAMSQANNSNIMMQRSNFKGPKRIVKCFNCGKEGHIARNCRAPRKKGCWKCGKEGHQMKDCTERQANFLGKIWP
SHKGRPGNFLQSRPEPTAPPAESFRFEETTPAPKQEPKDREPLTSLKSLFGSDPLSQ
>Gag_CON_C_2000s
MGARASILRGGKLDKWEKIRLRPGGKKHYMLKHLVWASRELERFALNPGLLETSEGCKQIIKQLQPALQTGTEELRSLYNTVATLYCV
HEKIEVRDTKEALDKIEEEQNKSQQKTQQAKAADGKVSQNYPIVQNLQGQMVHQAISPRTLNAWVKVIEEKAFSPEVIPMFTALSEGA
TPQDLNTMLNTVGGHQAAMQMLKDTINEEAAEWDRLHPVHAGPIAPGQMREPRGSDIAGTTSTLQEQIAWMTSNPPIPVGDIYKRWI
ILGLNKIVRMYSPVSILDIKQGPKEPFRDYVDRFFKTLRAEQATQDVKNWMTDTLLVQNANPDCKTILRALGPGATLEEMMTACQGVG
GPGHKARVLAEAMSQANNTNIMMQRSNFKGPKRIVKCFNCGKEGHIARNCRAPRKKGCWKCGKEGHQMKDCTERQANFLGKIWP
SHKGRPGNFLQSRPEPTAPPAESFRFEETTPAPKQEPKDREPLTSLKSLFGSDPLSQ
>Gag_CON_C_2010s
MGARASILRGGKLDKWEKIRLRPGGKKHYMLKHLVWASRELERFALNPGLLETAEGCKQIIKQLQPALQTGTEELRSLYNTVATLYCV
HAGIEVRDTKEALDKIEEEQNKSQQKTQQAKAADGKVSQNYPIVQNLQGQMVHQAISPRTLNAWVKVIEEKAFSPEVIPMFTALSEGA
TPQDLNTMLNTVGGHQAAMQMLKDTINEEAAEWDRLHPVHAGPVAPGQMREPRGSDIAGTTSTLQEQIAWMTSNPPIPVGDIYKRW
IILGLNKIVRMYSPVSILDIKQGPKEPFRDYVDRFFKTLRAEQATQDVKNWMTDTLLVQNANPDCKTILRALGPGATLEEMMTACQGV
GGPSHKARVLAEAMSQANNSNIMMQRSNFKGPKRIVKCFNCGKEGHIARNCRAPRKKGCWKCGKEGHQMKDCTERQANFLGKIW
PSHKGRPGNFLQSRPEPTAPPAESFRFEETTPAPKQEPKDREPLTSLKSLFGSDPLSQ
>Gag_CON_D_1980s
MGARASVLSGGKLDAWERIRLRPGGKKKYRLKHIVWASRELERFALNPGLLETSEGCKQIIGQLQPAIQTGSEELRSLYNTVATLYCV
HERIDVKDTKEALEKIEEEQNKSKKKAQQAAADTGNSSQVSQNYPIVQNLQGQMVHQAISPRTLNAWVKVIEEKAFSPEVIPMFSALS
EGATPQDLNTMLNTVGGHQAAMQMLKETINEEAAEWDRLHPVHAGPIAPGQMREPRGSDIAGTTSTLQEQIAWMTSNPPIPVGEIYK
RWIILGLNKIVRMYSPVSILDIRQGPKEPFRDYVDRFYKTLRAEQASQDVKNWMTETLLVQNANPDCKTILKALGPQATLEEMMTACQ
GVGGPSHKARVLAEAMSQATNSATAVMMQRGNFKGQRKIIKCFNCGKEGHIAKNCRAPRKKGCWKCGREGHQMKDCTERQANFL
GKIWPSHKGRPGNFLQSRPEPTAPPAESFGFGEEITPSQKQEQKDKELYPLTSLKSLFGNDPLSQ
>Gag_CON_D_1990s
MGARASVLSGGKLDEWEKIRLRPGGKKKYRLKHIVWASRELERFALNPGLLETSEGCKQILGQLQPAIQTGSEELKSLFNTVATLYCV
HERIKVKDTKEALDKIEEEQNKSKKKAQQATADTRNSSQVSQNYPIVQNLQGQMVHQAISPRTLNAWVKVIEEKAFSPEVIPMFSALS
EGATPQDLNTMLNTVGGHQAAMQMLKETINEEAAEWDRLHPVHAGPIAPGQMREPRGSDIAGTTSTLQEQIGWMTSNPPIPVGEIYK
RWIILGLNKIVRMYSPVSILDIRQGPKEPFRDYVDRFYKTLRAEQASQDVKNWMTETLLVQNANPDCKTILKALGPAATLEEMMTACQ
GVGGPSHKARVLAEAMSQATNANAAIMMQRGNFKGPRKIIKCFNCGKEGHIAKNCRAPRKKGCWKCGKEGHQMKDCTERQANFLG
KIWPSHKGRPGNFLQSRPEPTAPPAESFGFGEEITPSQKQEQKDKELYPLASLKSLFGNDPLSQ
>Gag_CON_D_2000s
MGARASVLSGGKLDEWEKIRLRPGGKKKYKLKHIVWASRELERFALNPGLLETSEGCKQIIGQLQPAIQTGSEELKSLYNTVATLYCV
HERIKVADTKEALDKIEEEQTKSKKKAQQATADTGNSSQVSQNYPIVQNLQGQMVHQAISPRTLNAWVKVIEEKAFSPEVIPMFSALS
EGATPQDLNTMLNTVGGHQAAMQMLKETINEEAAEWDRLHPVHAGPVAPGQMREPRGSDIAGTTSTLQEQIAWMTSNPPIPVGEIY
KRWIILGLNKIVRMYSPVSILDIRQGPKEPFRDYVDRFYKTLRAEQASQDVKNWMTETLLVQNANPDCKTILKALGPGATLEEMMTAC
QGVGGPSHKARVLAEAMSQATNTNAAIMMQRGNFKGPRKIIKCFNCGKEGHIAKNCRAPRKKGCWKCGKEGHQMKDCTERQANFL
GKIWPSHKGRPGNFLQSRPEPTAPPAESFGFGEEITPSQKQEQKDKELYPLASLKSLFGNDPLSQ
>Gag_CON_D_2010s
MGARASVLSGGKLDEWEKIRLRPGGKKKYKLKHIVWASRELERFALNPGLLETSEGCKQILGQLQPAIQTGSEELKSLYNTVATLYCV
HERIKVADTKEALDKIEEEQNKSKKKAQQAAADTRNSSQVSQNYPIVQNLQGQMVHQAISPRTLNAWVKVIEEKAFSPEVIPMFSALS
EGATPQDLNTMLNTVGGHQAAMQMLKETINEEAAEWDRLHPVHAGPVAPGQMREPRGSDIAGTTSTLQEQIGWMTSNPPIPVGEIY
KRWIILGLNKIVRMYSPVSILDIRQGPKEPFRDYVDRFYKTLRAEQASQDVKNWMTETLLVQNANPDCKTILKALGPAATLEEMMTAC
QGVGGPSHKARVLAEAMSQATNNAAIMMQRGNFKGPRKIIKCFNCGKEGHLAKNCRAPRKKGCWKCGREGHQMKDCTERQANFL
GKIWPSHKGRPGNFLQSRPEPTAPPAESFGFGEEITPSQKQEQKDKELYPLASLKSLFGNDPLSQ
>Gag_CON_F1_2000s
MGARASVLSGGKLDAWEKIRLRPGGKKKYRMKHLVWASRELERFALDPGLLETSEGCRKIIGQLQPSLQTGSEELKSLYNTVAVLYY
VHQRVEVKDTKEALDKLEEEQNKSQQKTQQAAADKGVSQNYPIVQNLQGQMVHQSLSPRTLNAWVKVIEEKAFSPEVIPMFSALSE
GATPQDLNTMLNTVGGHQAAMQMLKDTINEEAAEWDRLHPVHAGPIPPGQMREPRGSDIAGTTSTLQEQIQWMTSNPPVPVGEIYK
RWIILGLNKIVRMYSPVSILDIKQGPKEPFRDYVDRFFKTLRAEQATQEVKGWMTDTLLVQNANPDCKTILKALGPGATLEEMMTACQ
GVGGPSHKARVLAEAMSQATNTAVMMQKSNFKGQRRIVKCFNCGKEGHIAKNCRAPRKKGCWKCGREGHQMKDCTERQANFLG
KIWPSHKGRPGNFLQNRPEPTAPPAESFGFGEEITPSPKQEQKDEGLYPPLASLKSLFGNDPSQ
>Gag_CON_G_2000s
MGARASVLSGGKLDAWEKIRLRPGGKKKYRMKHLVWASRELERFALNPGLLETAEGCQQIMVQLQPALQTGTEELKSLFNTVATLY
CVHQRIEVKDTKEALEEVEKIQKKSQQKTQQAAMDKGNSSQVSQNYPIVQNAQGQMVHQAISPRTLNAWVKVVEEKAFSPEVIPMFT
ALSEGATPQDLNTMLNTVGGHQAAMQMLKDTINEEAAEWDRLHPQQAGPIPPGQIREPRGSDIAGTTSTLQEQIAWMTSNPPIPVGE
IYKRWIILGLNKIVRMYSPVSILDIKQGPKEPFRDYVDRFFKTLRAEQATQDVKNWMTDTLLVQNANPDCKTILRALGPGATLEEMMTA
CQGVGGPSHKARVLAEAMSQASGAAAAIMMQKSNFKGPRRNIKCFNCGKEGHLARNCRAPRKKGCWKCGKEGHQMKDCTERQA
NFLGKIWPSNKGRPGNFLQNRPEPTAPPAESFGFGEEIAPSPKQEPREKELYPLASLKSLFGNDPSQ
>Gag_CON_G_2010s
MGARASVLSGGKLDAWEKIRLRPGGKKKYRLKHLVWASRELERFALNPGLLETAEGCQQIMQQLQPALQTGTEEIKSLYNTVATLYC
VHQRIEVKDTKEALDEVEKIQKKSQQKTQKAATGKGNSSQVSQNYPIVQNAQGQMVHQAISPRTLNAWVKVIEEKAFSPEVIPMFTAL
SEGATPQDLNTMLNTVGGHQAAMQMLKDTINEEAAEWDRLHPPQAGPIPPGQIREPRGSDIAGTTSTLQEQITWMTSNPPIPVGEIY
KRWIILGLNKIVRMYSPVSILDIKQGPKEPFRDYVDRFFKTLRAEQATQEVKGWMTDTLLVQNANPDCKTILRALGPGATLEEMMTAC
QGVGGPSHKARVLAEAMSQASGAAAAIMMQKSNFKGPRRNIKCFNCGKEGHLARNCRAPRKKGCWKCGKEGHQMKDCTERQAN
FLGKIWPSNKGRPGNFLQNRLEPTAPPAESFGFGEEIAPSQKQEPREKETPPLTSLKSLFGNDPSQ
>Gag_CON_01_AE_1990s

MGARASVLSGGKLDAWEKIRLRPGGKKKYRMKHLVWASRELERFALNPGLLETAEGCQQIIEQLQSTLKTGSEELKSLFNTVVTLWC
VHQRIEVKDTKEALDKIEEVQNKSQQKTQQAAAGTGSSSKVSQNYPIVQNAQGQMVHQPLSPRTLNAWVKVVEEKGFNPEVIPMFS
ALSEGATPQDLNMMLNIVGGHQAAMQMLKETINEEAAEWDRVHPVHAGPIPPGQMREPRGSDIAGTTSTLQEQIGWMTNNPPIPVG
DIYKRWIILGLNKIVRMYSPVSILDIRQGPKEPFRDYVDRFYKTLRAEQATQEVKNWMTETLLVQNANPDCKSILKALGTGATLEEMMT
ACQGVGGPSHKARVLAEAMSHAQHANIMMQRGNFKGQKRIKCFNCGKEGHLARNCRAPRKKGCWKCGKEGHQMKDCTERQANF
LGKIWPSNKGRPGNFPQSRPEPTAPPAENWGMGEEITSLLKQEQKDKEHPPPLVSLKSLFGNDPLSQ
>Gag_CON_01_AE_2000s
MGARASVLSGGKLDAWEKIRLRPGGKKKYRMKHLVWASRELERFALNPGLLETAEGCQQIIEQLQSTLKTGSEELKSLFNTVATLWC
VHQRIEVKDTKEALDKIEEVQNKSQQKTQQAAAGTGSSSKVSQNYPIVQNAQGQMVHQPLSPRTLNAWVKVVEEKGFNPEVIPMFS
ALSEGATPQDLNMMLNIVGGHQAAMQMLKETINEEAAEWDRVHPVHAGPIPPGQMREPRGSDIAGTTSTLQEQIGWMTNNPPIPVG
DIYKRWIILGLNKIVRMYSPVSILDIRQGPKEPFRDYVDRFYKTLRAEQATQEVKNWMTETLLVQNANPDCKSILKALGTGATLEEMMT
ACQGVGGPSHKARVLAEAMSQAQHANIMMQRGNFKGQKRIKCFNCGKEGHLARNCRAPRKKGCWKCGKEGHQMKDCTERQANF
LGKIWPSNKGRPGNFPQSRPEPTAPPAENWGMGEEITSLLKQEQKDKEHPPPSVSLKSLFGNDPLSQ
>Gag_CON_01_AE_2010s
MGARASVLSGGKLDAWEKIRLRPGGKKKYRMKHLVWASRELERFALNPGLLETAEGCQQIIEQLQSTLKTGSEELKSLFNTVATLWC
VHQRIEVKDTKEALDKIEEVQKKSQQKTQQAAAGTGSSSKVSQNYPIVQNAQGQMVHQPVSPRTLNAWVKVVEEKGFNPEVIPMFS
ALSEGATPQDLNMMLNIVGGHQAAMQMLKETINEEAAEWDRVHPVHAGPIPPGQMREPRGSDIAGTTSTLQEQIGWMTNNPPIPVG
DIYKRWIILGLNKIVRMYSPVSILDIRQGPKEPFRDYVDRFYKTLRAEQATQEVKNWMTETLLVQNANPDCKSILKALGTGATLEEMMT
ACQGVGGPSHKARVLAEAMSQAQQTNIMMQRGNFKGQKRIKCFNCGKEGHLARNCRAPRKKGCWKCGKEGHQMKDCTERQANF
LGKIWPSNKGRPGNFPQSRPEPTAPPAENWGMGEEITSLLKQEQKDKEHPPPSVSLKSLFGNDPLSQ
>Gag_CON_02_AG_1990s
MGARASVLSGGKLDAWEKIRLRPGGKKKYRLKHLVWASRELERFALNPGLLETAEGCQQLMEQLQSTLRTGSEELKSLFNTIATLWC
VHQRIDIKDTKEALDKIEEIQNKSKQKTQQAAAATGSSSQNYPIVQNAQGQMVHQSMSPRTLNAWVKVIEEKAFSPEVIPMFSALSEG
ATPQDLNMMLNIVGGHQAAMQMLKDTINEEAAEWDRVHPVHAGPIPPGQMREPRGSDIAGTTSTLQEQIGWMTSNPPIPVGEIYKR
WIVLGLNKIVRMYSPVSILDIRQGPKEPFRDYVDRFFKTLRAEQATQEVKNWMTETLLVQNANPDCKSILRALGPGATLEEMMTACQG
VGGPGHKARVLAEAMSQVQQSNIMMQRGNFRGQRTIKCFNCGKEGHLARNCKAPRKRGCWKCGKEGHQMKDCTERQANFLGKI
WPSSKGRPGNFPQSRPEPTAPPAESFGMGEEITSSPKQEPRDKGLYPPLTSLKSLFGNDPSQ
>Gag_CON_02_AG_2000s
MGARASVLSGGKLDAWEKIRLRPGGKKKYRLKHLVWASRELERFALNPGLLETAEGCQQLMEQLQSTLKTGSEELKSLFNTIATLWC
VHQRIEIKDTKEALDKIEEIQNKSKQKTQQAAAATGSSSQNYPIVQNAQGQMVHQSMSPRTLNAWVKVIEEKAFSPEVIPMFSALSEG
ATPQDLNMMLNIVGGHQAAMQMLKDTINEEAAEWDRTHPVHAGPIPPGQMREPRGSDIAGTTSTLQEQIGWMTSNPPIPVGEIYKR
WIVLGLNKIVRMYSPVSILDIRQGPKEPFRDYVDRFFKTLRAEQATQEVKNWMTETLLVQNANPDCKSILRALGPGATLEEMMTACQG
VGGPGHKARVLAEAMSQVQQSNIMMQRGNFRGQRTIKCFNCGKEGHLARNCKAPRKRGCWKCGKEGHQMKDCTERQANFLGKI
WPSSKGRPGNFPQSRPEPTAPPAESFGMGEEITSSPKQEPRDKGLYPPLTSLKSLFGNDPSQ
>Gag_CON_02_AG_2010s
MGARASVLSGGKLDAWEKIRLRPGGKKKYRLKHLVWASRELERFALNPGLLETAEGCQQIMEQLQSALKTGSEELKSLFNTIATLWC
VHQRIDIKDTKEALDKIEEVQNKSKQKTQQAAAATGSSSQNYPIVQNAQGQMVHQSMSPRTLNAWVKVIEEKAFSPEVIPMFSALSE
GATPQDLNMMLNIVGGHQAAMQMLKDTINEEAAEWDRTHPVHAGPIPPGQMREPRGSDIAGTTSTLQEQIGWMTSNPPIPVGEIYK
RWIILGLNKIVRMYSPVSILDIRQGPKEPFRDYVDRFFKTLRAEQATQEVKNWMTETLLVQNANPDCKSILRALGPGATLEEMMTACQ
GVGGPGHKARVLAEAMSQVQQANIMMQRGNFRGQRTIKCFNCGKEGHLARNCKAPRKKGCWKCGKEGHQMKDCTERQANFLGK
IWPSSKGRPGNFPQSRPEPTAPPAESLGMGEEITSSPKQEPRDKGLYPPLTSLKSLFGNDPSQ
>Gag_CON_07_BC_2000s
MGARASILRGGKLDKWEKIRLRPGGKKHYMLKHLVWASRELERFALNPGLLETSEGCKQIMKQLQPALQTGTEELRSLFNTVATLYC
VHTGIDVRDTKEALDKIEEEQNKIQQKTQQAKEADGKVSQNYPIVQNLQGQMVHQPISPRTLNAWVKVVEEKAFSPEVIPMFSALSEG
ATPQDLNTMLNTVGGHQAAMQILKDTINEEAAEWDRLHPVHAGPIAPGQMREPRGSDIAGTTSTLQEQIAWMTSNPPVPVGDIYKR
WIILGLNKIVRMYSPTSILDIKQGPKEPFRDYVDRFFKTLRAEQATQDVKNWMTDTLLVQNANPDCKTILRALGPGASIEEMMTACQGV
GGPSHKARVLAEAMSQTNSAIMMQRSNFKGPKRIVKCFNCGKEGHIARNCRAPRKKGCWKCGKEGHQMKDCTERQANFLGKIWP
SHKGRPGNFLQSRPEPTAPPEESFRFGEETTTPSQKQEPIDKELYPLTSLKSLFGNDPSSQ
>Gag_CON_07_BC_2010s
MGARASILRGGKLDKWEKIRLRPGGKKHYMLKHLVWASRELERFALNPGLLETSEGCKQIMNQLQPALQTGTEELRSLFNTVATLYC
VHAGIDVRDTKEALDKIEEEQNKIQQKTQQAKEADRKVSQNYPIVQNLQGQMVHQPISPRTLNAWVKVVEEKAFSPEVIPMFSALSEG
ATPQDLNTMLNTVGGHQAAMQILKDTINEEAAEWDRLHPVHAGPIAPGQMREPRGSDIAGTTSTLQEQIAWMTSNPPVPVGDIYKR
WIILGLNKIVRMYSPTSILDIKQGPKEPFRDYVDRFFKTLRAEQATQDVKNWMTDTLLVQNANPDCKTILRALGPGASIEEMMTACQGV
GGPSHKARVLAEAMSQTNNAIMMQRSNFKGPKRIVKCFNCGKEGHIARNCRAPRKKGCWKCGKEGHQMKDCTERQANFLGKIWP
SHKGRPGNFLQSRPEPTAPPEESFRFGEEITTPSQKQEPVTSLKSLFGNDPLLQ

File S6. Pol consensus sequences for each subtype/CRF and sampling period. Suffixes in the consensus sequence name indicate the subtype/CRF and decade during which sequences were sampled.

>Pol_CON_A1_1990s
FFRENLAFQQGEARKFSSEQTGANSPTSRDLWDGGRDSLPSEAGAERQGTGPTFSFPQITLWQRPLVTVKIGGQLKEALLDTGADD
TVLEDINLPGKWKPKMIGGIGGFIKVKQYDQILIEICGKKAIGTVLVGPTPVNIIGRNMLTQIGCTLNFPISPIETVPVKLKPGMDGPKVKQ
WPLTEEKIKALTEICTEMEKEGKISKIGPENPYNTPIFAIKKKDSTKWRKLVDFRELNKRTQDFWEVQLGIPHPAGLKKKKSVTVLDVGD
AYFSVPLDESFRKYTAFTIPSTNNETPGIRYQYNVLPQGWKGSPAIFQSSMTKILEPFRSKNPEIIIYQYMDDLYVGSDLEIGQHRAKIE
ELRAHLLSWGFTTPDKKHQKEPPFLWMGYELHPDKWTVQPIQLPEKESWTVNDIQKLVGKLNWASQIYAGIKVKQLCKLLRGAKALT
DIVTLTEEAELELAENREILKDPVHGVYYDPSKDLIAEIQKQGQDQWTYQIYQEPFKNLKTGKYARKRSAHTNDVKQLAEVVQKVVME
SIVIWGKTPKFKLPIQKETWETWWMDYWQATWIPEWEFVNTPPLVKLWYQLEKDPIAGAETFYVDGAANRETKLGKAGYVTDRGRQ
KVVSLTETTNQKTELHAIYLALQDSGSEVNIVTDSQYALGIIQAQPDRSESELVNQIIEKLIGKDKVYLSWVPAHKGIGGNEQVDKLVSS
GIRKVLFLDGIDKAQEEHERYHSNWRAMASDFNLPPIVAKEIVASCDKCQLKGEAMHGQVDCSPGIWQLDCTHLEGKVILVAVHVAS
GYIEAEVIPAETGQETAYFLLKLAGRWPVKVVHTDNGSNFTSAAVKAACWWANIQQEFGIPYNPQSQGVVESMNKELKKIIGQVREQ
AEHLKTAVQMAVFIHNFKRKGGIGGYSAGERIIDIIATDIQTKELQKQITKIQNFRVYYRDSRDPIWKGPAKLLWKGEGAVVIQDNSDIKV
VPRRKAKIIRDYGKQMAGDDCVAGRQDED
>Pol_CON_A1_2000s
FFRENLAFQQGEARKFSSEQTRANSPTSRDLWDGGRDSLPSEAGAERQGTGPTLSFPQITLWQRPLVTVKIGGQLKEALLDTGADD
TVLEDINLPGKWKPKMIGGIGGFIKVKQYDQILIEICGKKAIGTVLVGPTPVNIIGRNMLTQIGCTLNFPISPIETVPVKLKPGMDGPKVKQ
WPLTEEKIKALTEICTEMEKEGKISKIGPENPYNTPIFAIKKKDSTKWRKLVDFRELNKRTQDFWEVQLGIPHPAGLKKKKSVTVLDVGD
AYFSVPLDESFRKYTAFTIPSTNNETPGIRYQYNVLPQGWKGSPAIFQSSMTKILEPFRSKNPEIIIYQYMDDLYVGSDLEIGQHRAKIE
ELRAHLLSWGFTTPDKKHQKEPPFLWMGYELHPDKWTVQPIQLPEKESWTVNDIQKLVGKLNWASQIYAGIKVKQLCKLLRGAKALT
DIVTLTEEAELELAENREILKDPVHGVYYDPSKDLIAEIQKQGQDQWTYQIYQEPFKNLKTGKYARKRSAHTNDVKQLAEVVQKVVME
SIVIWGKTPKFKLPIQKETWETWWMDYWQATWIPEWEFVNTPPLVKLWYQLEKDPIVGAETFYVDGAANRETKLGKAGYVTDRGRQ
KVVSLTETTNQKTELHAIYLALQDSGSEVNIVTDSQYALGIIQAQPDRSESELVNQIIEKLIGKDKVYLSWVPAHKGIGGNEQVDKLVSS
GIRKVLFLDGIDKAQEEHERYHSNWRAMASDFNLPPIVAKEIVASCDKCQLKGEAMHGQVDCSPGIWQLDCTHLEGKVILVAVHVAS
GYIEAEVIPAETGQETAYFLLKLAGRWPVKVVHTDNGSNFTSAAVKAACWWANIQQEFGIPYNPQSQGVVESMNKELKKIIGQVREQ
AEHLKTAVQMAVFIHNFKRKGGIGGYSAGERIIDIIATDIQTKELQKQITKIQNFRVYYRDSRDPIWKGPAKLLWKGEGAVVIQDNSDIKV
VPRRKAKIIRDYGKQMAGDDCVAGRQDED
>Pol_CON_A1_2010s
FFRENLAFQQGEARKFSSEQTRANSPTSRDLWDGGRDSLPSEAGAERQGTEPTFSFPQITLWQRPLVTVKIGGQLKEALLDTGADD
TVLEDINLPGKWKPKMIGGIGGFIKVKQYDQILIEICGKKAIGTVLVGPTPVNIIGRNMLTQIGCTLNFPISPIETVPVKLKPGMDGPKVKQ
WPLTEEKIKALTEICTEMEKEGKISKIGPENPYNTPIFAIKKKDSTKWRKLVDFRELNKRTQDFWEVQLGIPHPAGLKKKKSVTVLDVGD
AYFSVPLDENFRKYTAFTIPSTNNETPGIRYQYNVLPQGWKGSPAIFQSSMTKILEPFRSKNPEIIIYQYMDDLYVGSDLEIGQHRTKIE
ELRAHLLSWGFTTPDKKHQKEPPFLWMGYELHPDKWTVQPIQLPEKESWTVNDIQKLVGKLNWASQIYAGIKVKQLCKLLRGAKALT
DVVTLTEEAELELAENREILKDPVHGVYYDPSKDLIAEIQKQGQDQWTYQIYQEPFKNLKTGKYARKRSAHTNDVKQLTEVVQKVVME
SIVIWGKTPKFKLPIQKETWETWWMDYWQATWIPEWEFVNTPPLVKLWYQLEKDPIAGAETFYVDGAANRETKLGKAGYVTDRGRQ
KVVSLTETTNQKTELHAIHLALQDSGSEVNIVTDSQYALGIIQAQPDRSESELVNQIIEKLIGKDKVYLSWVPAHKGIGGNEQVDKLVSS
GIRKVLFLDGIDKAQEEHERYHSNWRAMASDFNLPPIVAKEIVASCDKCQLKGEAMHGQVDCSPGIWQLDCTHLEGKVILVAVHVAS
GYIEAEVIPAETGQETAYFLLKLAGRWPVKVVHTDNGSNFTSAAFKAACWWAGIQQEFGIPYNPQSQGVVESMNKELKKIIGQVREQ
AEHLKTAVQMAVFIHNFKRKGGIGGYSAGERIIDIIATDIQTKELQKQITKIQNFRVYYRDSRDPIWKGPAKLLWKGEGAVVIQDNSDIKV
VPRRKAKIIRDYGKQMAGDDCVAGRQDED
>Pol_CON_A6_2000s
FFRENLAFQQREARKFSSEQTRAISPTSRKLWDGGRDNPLPETGTERQGTASSFNFPQITLWQRPLVTVKIGGQLKEALLDTGADDT
VLEDINLPGKWKPKMIGGIGGFIKVRQYDQILIEICGKKAIGTVLVGPTPVNIIGRNMLTQLGCTLNFPISPIETVPVTLKPGMDGPKVKQ
WPLTEEKIKALTDICKEMEKEGKISKIGPENPYNTPVFAIKKKDSTKWRKLVDFRELNKRTQDFWEVQLGIPHPAGLKKKKSVTVLDVG
DAYFSVPLDESFRKYTAFTIPSINNETPGIRYQYNVLPQGWKGSPSIFQSSMTKILEPFRLKNPEIVIYQYMDDLYVGSDLEIGQHRTKIE
ELRAHLLSWGFTTPDKKHQKEPPFLWMGYELHPDKWTVQPIMLPDKDSWTVNDIQKLVGKLNWASQIYPGIKVRQLCKLLRGAKALT
DIVTLTEEAELELAENREILKEPVHGVYYDPSKDLVAEIQKQGQDQWTYQIYQEPFKNLKTGKYAKKGSAHTNDVKQLTAVVQKVATE
SIVIWGKTPKFRLPIQKETWEAWWMEYWQATWIPEWEFVNTPPLVKLWYQLEKEPIVGAETFYVDGAANRETKIGKAGYVTDRGRQ
KVVPLTETTNQKTELHAIHLALQDSGSEVNIVTDSQYALGIIQAQPDRSESEIVNKIIEKLIEKERVYLSWVPAHKGIGGNEQVDKLVSNG
IRRVLFLEGIDKAQEEHEKYHSNWKAMASDFNLPPIVAKEIVASCDKCQLKGEAMHGQVDCSPGIWQLDCTHLEGKVIIVAVHVASGYI
EAEVIPAETGQETAYFLLKLAGRWPVKVVHTDNGPNFTSSAVKAACWWANIQQEFGIPYNPQSQGVVESMNKELKKIIGQVREQAEH
LKTAVQMAVFIHNFKRKGGIGGYSAGERIIDIIATDIQTKELQKQITKIQNFRVYYRDSRDPIWKGPAKLLWKGEGAVVIQDNNDIKVVPR
RKAKIIRDYGKQMAGDDCVASRQDED
>Pol_CON_A6_2010s
FFRENLAFQQREARKFSSEQTRTISPTSRELWDGGRDNPLPETGTERQGTASSFNFPQITLWQRPLVTVKIGGQLKEALLDTGADDT
VLEDINLPGKWKPKMIGGIGGFIKVRQYDQILIEICGKKAIGTVLVGPTPVNIIGRNMLTQLGCTLNFPISPIETVPVTLKPGMDGPKVKQ
WPLTEEKIKALTDICKEMEKEGKISKIGPENPYNTPVFAIKKKDSTKWRKLVDFRELNKRTQDFWEVQLGIPHPAGLKKKKSVTVLDVG
DAYFSVPLDESFRKYTAFTIPSINNETPGIRYQYNVLPQGWKGSPAIFQSSMTKILEPFRLKNPEIVIYQYMDDLYVGSDLEIGQHRTKIE
ELRAHLLSWGFTTPDKKHQKEPPFLWMGYELHPDKWTVQPIMLPDKDSWTVNDIQKLVGKLNWASQIYPGIKVKQLCKLLRGAKALT
DIVTLTEEAELELAENREILKEPVHGVYYDPSKDLVAEIQKQGQDQWTYQIYQEPFKNLKTGKYAKKGSAHTNDVKQLTAVVQKVATE
SIVIWGKTPKFRLPIQKETWEAWWMEYWQATWIPEWEFVNTPPLVKLWYQLEKEPIVGAETFYVDGAANRETKIGKAGYVTDRGRQ
KVVPLTETTNQKTELHAIHLALQDSGSEVNIVTDSQYALGIIQAQPDRSESEIVNKIIEKLIEKERVYLSWVPAHKGIGGNEQVDKLVSNG
IRRVLFLDGIDKAQEEHEKYHSNWKAMASDFNLPPIVAKEIVASCDKCQLKGEAMHGQVDCSPGIWQLDCTHLEGKVIIVAVHVASGYI
EAEVIPAETGQETAYFLLKLAGRWPVKVVHTDNGPNFTSSAVKAACWWANIQQEFGIPYNPQSQGVVESMNKELKKIIGQVREQAEH

LKTAVQMAVFIHNFKRKGGIGGYSAGERIIDIIATDIQTKELQKQITKIQNFRVYYRDSRDPIWKGPAKLLWKGEGAVVIQDNNDIKVVPR
RKAKIIRDYGKQMAGDDCVASRQDED
>Pol_CON_B_1980s
FFREDLAFPQGKAREFSSEQTRANSPTRRELQVWGRDNNSLSEAGADRQGTVSFSFPQITLWQRPLVTIKIGGQLKEALLDTGADDT
VLEEMNLPGRWKPKMIGGIGGFIKVRQYDQILIEICGHKAIGTVLVGPTPVNIIGRNLLTQIGCTLNFPISPIETVPVKLKPGMDGPKVKQ
WPLTEEKIKALVEICTEMEKEGKISKIGPENPYNTPVFAIKKKDSTKWRKLVDFRELNKRTQDFWEVQLGIPHPAGLKKKKSVTVLDVG
DAYFSVPLDKDFRKYTAFTIPSINNETPGIRYQYNVLPQGWKGSPAIFQSSMTKILEPFRKQNPDIVIYQYMDDLYVGSDLEIGQHRTKI
EELRQHLLRWGFTTPDKKHQKEPPFLWMGYELHPDKWTVQPIVLPEKDSWTVNDIQKLVGKLNWASQIYAGIKVKQLCKLLRGTKAL
TEVIPLTEEAELELAENREILKEPVHGVYYDPSKDLIAEIQKQGQGQWTYQIYQEPFKNLKTGKYARMRGAHTNDVKQLTEAVQKIATE
SIVIWGKTPKFKLPIQKETWEAWWTEYWQATWIPEWEFVNTPPLVKLWYQLEKEPIVGAETFYVDGAANRETKLGKAGYVTDRGRQ
KVVSLTDTTNQKTELQAIHLALQDSGLEVNIVTDSQYALGIIQAQPDKSESELVSQIIEQLIKKEKVYLAWVPAHKGIGGNEQVDKLVSA
GIRKVLFLDGIDKAQEEHEKYHSNWRAMASDFNLPPVVAKEIVASCDKCQLKGEAMHGQVDCSPGIWQLDCTHLEGKVILVAVHVAS
GYIEAEVIPAETGQETAYFLLKLAGRWPVKTIHTDNGSNFTSTTVKAACWWAGIKQEFGIPYNPQSQGVVESMNKELKKIIGQVRDQA
EHLKTAVQMAVFIHNFKRKGGIGGYSAGERIVDIIATDIQTKELQKQITKIQNFRVYYRDSRDPLWKGPAKLLWKGEGAVVIQDNSDIKV
VPRRKAKIIRDYGKQMAGDDCVASRQDED
>Pol_CON_B_1990s
FFRENLAFPQGKAREFSSEQTRANSPTRRELQVWGRDNNSLSEAGADRQGTVSFSFPQITLWQRPLVTIKIGGQLKEALLDTGADDT
VLEEMNLPGRWKPKMIGGIGGFIKVRQYDQIPIEICGHKAIGTVLVGPTPVNIIGRNLLTQIGCTLNFPISPIETVPVKLKPGMDGPKVKQ
WPLTEEKIKALVEICTEMEKEGKISKIGPENPYNTPVFAIKKKDSTKWRKLVDFRELNKRTQDFWEVQLGIPHPAGLKKKKSVTVLDVG
DAYFSVPLDKDFRKYTAFTIPSINNETPGIRYQYNVLPQGWKGSPAIFQSSMTKILEPFRKQNPDIVIYQYMDDLYVGSDLEIGQHRTKI
EELRQHLLRWGFTTPDKKHQKEPPFLWMGYELHPDKWTVQPIVLPEKDSWTVNDIQKLVGKLNWASQIYAGIKVKQLCKLLRGTKAL
TEVVPLTEEAELELAENREILKEPVHGVYYDPSKDLIAEIQKQGQGQWTYQIYQEPFKNLKTGKYARMRGAHTNDVKQLTEAVQKIAT
ESIVIWGKTPKFKLPIQKETWEAWWTEYWQATWIPEWEFVNTPPLVKLWYQLEKEPIVGAETFYVDGAANRETKLGKAGYVTDRGR
QKVVSLTDTTNQKTELQAIHLALQDSGLEVNIVTDSQYALGIIQAQPDKSESELVSQIIEQLIKKEKVYLAWVPAHKGIGGNEQVDKLVS
AGIRKVLFLDGIDKAQEEHEKYHSNWRAMASDFNLPPVVAKEIVASCDKCQLKGEAMHGQVDCSPGIWQLDCTHLEGKIILVAVHVAS
GYIEAEVIPAETGQETAYFLLKLAGRWPVKTIHTDNGSNFTSTTVKAACWWAGIKQEFGIPYNPQSQGVVESMNKELKKIIGQVRDQA
EHLKTAVQMAVFIHNFKRKGGIGGYSAGERIVDIIATDIQTKELQKQITKIQNFRVYYRDSRDPLWKGPAKLLWKGEGAVVIQDNSDIKV
VPRRKAKIIRDYGKQMAGDDCVASRQDED
>Pol_CON_B_2000s
FFRENLAFPQGKAREFSSEQTRANSPTRRELQVWGRDNNSLSEAGADRQGTVSFSFPQITLWQRPLVTIKIGGQLKEALLDTGADDT
VLEEMNLPGRWKPKMIGGIGGFIKVRQYDQIPIEICGHKAIGTVLVGPTPVNIIGRNLLTQIGCTLNFPISPIETVPVKLKPGMDGPKVKQ
WPLTEEKIKALVEICTEMEKEGKISKIGPENPYNTPVFAIKKKDSTKWRKLVDFRELNKRTQDFWEVQLGIPHPAGLKKKKSVTVLDVG
DAYFSVPLDKDFRKYTAFTIPSINNETPGIRYQYNVLPQGWKGSPAIFQSSMTKILEPFRKQNPDIVIYQYMDDLYVGSDLEIGQHRTKI
EELRQHLLRWGFTTPDKKHQKEPPFLWMGYELHPDKWTVQPIVLPEKDSWTVNDIQKLVGKLNWASQIYAGIKVKQLCKLLRGTKAL
TEVVPLTEEAELELAENREILKEPVHGVYYDPSKDLIAEIQKQGQGQWTYQIYQEPFKNLKTGKYARMRGAHTNDVKQLTEAVQKIAT
ESIVIWGKTPKFRLPIQKETWEAWWTEYWQATWIPEWEFVNTPPLVKLWYQLEKEPIVGAETFYVDGAANRETKLGKAGYVTDRGR
QKVVSLTDTTNQKTELQAIHLALQDSGLEVNIVTDSQYALGIIQAQPDKSESELVSQIIEQLIKKEKVYLAWVPAHKGIGGNEQVDKLVS
AGIRKVLFLDGIDKAQEEHEKYHSNWRAMASDFNLPPVVAKEIVASCDKCQLKGEAMHGQVDCSPGIWQLDCTHLEGKIILVAVHVAS
GYIEAEVIPAETGQETAYFLLKLAGRWPVKTIHTDNGSNFTSTTVKAACWWAGIKQEFGIPYNPQSQGVVESMNKELKKIIGQVRDQA
EHLKTAVQMAVFIHNFKRKGGIGGYSAGERIVDIIATDIQTKELQKQITKIQNFRVYYRDSRDPLWKGPAKLLWKGEGAVVIQDNSDIKV
VPRRKAKIIRDYGKQMAGDDCVASRQDED
>Pol_CON_B_2010s
FFRENLAFPQGKAREFSSEQTRANSPTRRELQVWGRDNNSLSEAGADRQGTVSFSFPQITLWQRPLVTIKIGGQLKEALLDTGADDT
VLEEMNLPGRWKPKMIGGIGGFIKVRQYDQIPIEICGHKAIGTVLVGPTPVNIIGRNLLTQIGCTLNFPISPIETVPVKLKPGMDGPKVKQ
WPLTEEKIKALVEICTEMEKEGKISKIGPENPYNTPVFAIKKKDSTKWRKLVDFRELNKRTQDFWEVQLGIPHPAGLKKKKSVTVLDVG
DAYFSVPLDKDFRKYTAFTIPSINNETPGIRYQYNVLPQGWKGSPAIFQSSMTKILEPFRKQNPDIVIYQYMDDLYVGSDLEIGQHRTKI
EELRQHLLKWGFTTPDKKHQKEPPFLWMGYELHPDKWTVQPIVLPEKDSWTVNDIQKLVGKLNWASQIYPGIKVKQLCKLLRGTKAL
TEVVPLTEEAELELAENREILKEPVHGVYYDPSKDLIAEIQKQGQGQWTYQIYQEPFKNLKTGKYARMRGAHTNDVKQLTEAVQKIAT
ESIVIWGKTPKFRLPIQKETWEAWWTEYWQATWIPEWEFVNTPPLVKLWYQLEKEPIVGAETFYVDGAANRETKLGKAGYVTDRGR
QKVVSLTDTTNQKTELQAIHLALQDSGLEVNIVTDSQYALGIIQAQPDKSESELVSQIIEQLIKKEKVYLAWVPAHKGIGGNEQVDKLVS
AGIRKVLFLDGIDKAQEEHEKYHSNWRAMASDFNLPPVVAKEIVASCDKCQLKGEAMHGQVDCSPGIWQLDCTHLEGKIILVAVHVAS
GYIEAEVIPAETGQETAYFLLKLAGRWPVKTIHTDNGSNFTSTTVKAACWWAGIKQEFGIPYNPQSQGVVESMNKELKKIIGQVRDQA
EHLKTAVQMAVFIHNFKRKGGIGGYSAGERIIDIIATDIQTKELQKQITKIQNFRVYYRDSRDPLWKGPAKLLWKGEGAVVIQDNSDIKV
VPRRKAKIIRDYGKQMAGDDCVASRQDED
>Pol_CON_C_1990s
FFRENLAFPQGEAREFPSEQTRANSPTSRELQVRGDNPRSEAGAERQGTLNFPQITLWQRPLVSIKVGGQIKEALLDTGADDTVLEEI
NLPGKWKPKMIGGIGGFIKVRQYDQIPIEICGKKAIGTVLVGPTPVNIIGRNMLTQLGCTLNFPISPIETVPVKLKPGMDGPKVKQWPLT
EEKIKALTAICEEMEKEGKITKIGPENPYNTPVFAIKKKDSTKWRKLVDFRELNKRTQDFWEVQLGIPHPAGLKKKKSVTVLDVGDAYF
SVPLDEDFRKYTAFTIPSINNETPGIRYQYNVLPQGWKGSPAIFQSSMTKILEPFRAQNPEIVIYQYMDDLYVGSDLEIGQHRAKIEELR
EHLLKWGFTTPDKKHQKEPPFLWMGYELHPDKWTVQPIQLPEKDSWTVNDIQKLVGKLNWASQIYPGIKVRQLCKLLRGAKALTDIV
PLTEEAELELAENREILKEPVHGVYYDPSKDLIAEIQKQGHDQWTYQIYQEPFKNLKTGKYAKMRTAHTNDVKQLTEAVQKIAMESIVI
WGKTPKFRLPIQKETWETWWTDYWQATWIPEWEFVNTPPLVKLWYQLEKEPIAGAETFYVDGAANRETKIGKAGYVTDRGRQKIVS
LTETTNQKTELQAIQLALQDSGSEVNIVTDSQYALGIIQAQPDKSESELVNQIIEQLIKKERVYLSWVPAHKGIGGNEQVDKLVSSGIRK
VLFLDGIDKAQEEHEKYHSNWRAMASEFNLPPIVAKEIVASCDKCQLKGEAIHGQVDCSPGIWQLDCTHLEGKIILVAVHVASGYIEAE
VIPAETGQETAYYILKLAGRWPVKVIHTDNGSNFTSAAVKAACWWAGIQQEFGIPYNPQSQGVVESMNKELKKIIGQVRDQAEHLKTA
VQMAVFIHNFKRKGGIGGYSAGERIIDIIATDIQTKELQKQIIKIQNFRVYYRDSRDPIWKGPAKLLWKGEGAVVIQDNSDIKVVPRRKAK
IIKDYGKQMAGADCVAGRQDED
>Pol_CON_C_2000s
FFRENLAFPQGEAREFPSEQTRANSPTSRELQVRGDNPRSEAGAERQGTLNFPQITLWQRPLVSIKVGGQIKEALLDTGADDTVLEEI
NLPGKWKPKMIGGIGGFIKVRQYDQILIEICGKKAIGTVLVGPTPVNIIGRNMLTQLGCTLNFPISPIETVPVKLKPGMDGPKVKQWPLTE

EKIKALTAICEEMEKEGKITKIGPENPYNTPVFAIKKKDSTKWRKLVDFRELNKRTQDFWEVQLGIPHPAGLKKKKSVTVLDVGDAYFS
VPLDEGFRKYTAFTIPSINNETPGIRYQYNVLPQGWKGSPAIFQSSMTKILEPFRAQNPEIVIYQYMDDLYVGSDLEIGQHRAKIEELRE
HLLKWGFTTPDKKHQKEPPFLWMGYELHPDKWTVQPIQLPEKDSWTVNDIQKLVGKLNWASQIYPGIKVRQLCKLLRGAKALTDIVP
LTEEAELELAENREILKEPVHGVYYDPSKDLIAEIQKQGHDQWTYQIYQEPFKNLKTGKYAKMRTAHTNDVKQLTEAVQKIAMESIVIW
GKTPKFRLPIQKETWETWWTDYWQATWIPEWEFVNTPPLVKLWYQLEKEPIAGAETFYVDGAANRETKIGKAGYVTDRGRQKIVSLT
ETTNQKTELQAIQLALQDSGSEVNIVTDSQYALGIIQAQPDKSESELVNQIIEQLIKKERVYLSWVPAHKGIGGNEQVDKLVSSGIRKVL
FLDGIDKAQEEHEKYHSNWRAMASEFNLPPIVAKEIVASCDKCQLKGEAIHGQVDCSPGIWQLDCTHLEGKIILVAVHVASGYIEAEVIP
AETGQETAYYILKLAGRWPVKVIHTDNGSNFTSAAVKAACWWAGIQQEFGIPYNPQSQGVVESMNKELKKIIGQVRDQAEHLKTAVQ
MAVFIHNFKRKGGIGGYSAGERIIDIIATDIQTKELQKQIIKIQNFRVYYRDSRDPIWKGPAKLLWKGEGAVVIQDNSDIKVVPRRKVKIIK
DYGKQMAGADCVAGRQDED
>Pol_CON_C_2010s
FFRENLAFPQGEAREFPSEQTRANSPTSRELQVRGDNPRSEAGAERQGTLNFPQITLWQRPLVSIKVGGQIKEALLDTGADDTVLEEI
NLPGKWKPKMIGGIGGFIKVRQYDQIPIEICGKKAIGTVLVGPTPVNIIGRNMLTQLGCTLNFPISPIETVPVKLKPGMDGPKVKQWPLT
EEKIKALTAICEEMEKEGKITKIGPENPYNTPVFAIKKKDSTKWRKLVDFRELNKRTQDFWEVQLGIPHPAGLKKKKSVTVLDVGDAYF
SVPLDEDFRKYTAFTIPSINNETPGIRYQYNVLPQGWKGSPAIFQSSMTKILEPFRAKNPEIVIYQYMDDLYVGSDLEIGQHRAKIEELR
EHLLKWGFTTPDKKHQKEPPFLWMGYELHPDKWTVQPIQLPEKDSWTVNDIQKLVGKLNWASQIYPGIKVRQLCKLLRGAKALTDIV
PLTEEAELELAENREILKEPVHGVYYDPSKDLIAEIQKQGHDQWTYQIYQEPFKNLKTGKYAKMRTAHTNDVKQLTEAVQKIAMESIVI
WGKTPKFRLPIQKETWETWWTDYWQATWIPEWEFVNTPPLVKLWYQLEKEPIAGAETFYVDGAANRETKLGKAGYVTDRGRQKIVS
LTETTNQKTELQAIQLALQDSGSEVNIVTDSQYALGIIQAQPDKSESELVNQIIEQLIKKERVYLSWVPAHKGIGGNEQVDKLVSSGIRK
VLFLDGIDKAQEEHEKYHSNWRAMASEFNLPPIVAKEIVASCDKCQLKGEAMHGQVDCSPGIWQLDCTHLEGKIILVAVHVASGYIEA
EVIPAETGQETAYFILKLAGRWPVKVIHTDNGSNFTSAAVKAACWWAGIQQEFGIPYNPQSQGVVESMNKELKKIIGQVRDQAEHLKT
AVQMAVFIHNFKRKGGIGGYSAGERIIDIIATDIQTKELQKQIIKIQNFRVYYRDSRDPIWKGPAKLLWKGEGAVVIQDNSDIKVVPRRKA
KIIRDYGKQMAGADCVAGRQDED
>Pol_CON_D_1990s
FFRENLAFPQGKARELPSEQTRANSPTSRELRVWGGDNTLSETGAERQGTVSFSFPQITLWQRPLVTVKIGGQLKEALLDTGADDTV
LEEMNLPGKWKPKMIGGIGGFIKVRQYDQILVEICGHKAIGTVLVGPTPVNIIGRNLLTQIGCTLNFPISPIETVPVKLKPGMDGPKVKQ
WPLTEEKIKALTEICTEMEKEGKISRIGPENPYNTPIFAIKKKDSTKWRKLVDFRELNKRTQDFWEVQLGIPHPAGLKKKKSVTVLDVG
DAYFSVPLYEDFRKYTAFTIPSINNETPGIRYQYNVLPQGWKGSPAIFQSSMTKILEPFRKQNPEMVIYQYMDDLYVGSDLEIGQHRIKI
EELREHLLKWGFTTPDKKHQKEPPFLWMGYELHPDKWTVQPIKLPEKESWTVNDIQKLVGKLNWASQIYPGIKVRQLCKCLRGAKAL
TEVVPLTEEAELELAENREILKEPVHGVYYDPSKDLIAEIQKQGQDQWTYQIYQEQYKNLKTGKYAKMRGTHTNDVKQLTEAVQKIAQ
ECIVIWGKTPKFRLPIQKETWETWWTEYWQATWIPEWEFVNTPPLVKLWYQLEKEPIVGAETFYVDGAANRETKLGKAGYVTDRGR
QKVVSLTDTTNQKTELQAINLALQDSGLEVNIVTDSQYALGIIQAQPDKSESELVSQIIEQLIKKEKVYLSWVPAHKGIGGNEQVDKLVS
NGIRKILFLDGIDKAQEEHEKYHNNWRAMASDFNLPPVVAKEIVASCDKCQLKGEALHGQVDCSPGIWQLDCTHLEGKVILVAVHVAS
GYIEAEVIPAETGQETAYFLLKLAGRWPVKVVHTDNGSNFTSAAVKAACWWAGIKQEFGIPYNPQSQGVVESMNKELKKIIGQVRDQ
AEHLKTAVQMAVFIHNFKRKGGIGGYSAGERIIDIIATDIQTKELQKQIIKIQNFRVYYRDSRDPIWKGPAKLLWKGEGAVVIQDNSEIKV
VPRRKAKIIRDYGKQMAGDDCVASRQDED
>Pol_CON_D_2000s
FFRENLAFPQGKARELPSEQTRANSPTSRELRVWGGDNPLSETGAERQGTVSLSFPQITLWQRPLVTVKIGGQLKEALLDTGADDTV
LEEINLPGKWKPKMIGGIGGFIKVRQYDQILVEICGHKAIGTVLVGPTPVNIIGRNLLTQIGCTLNFPISPIETVPVKLKPGMDGPKVKQW
PLTEEKIKALTEICTEMEKEGKISRIGPENPYNTPIFAIKKKDSTKWRKLVDFRELNKRTQDFWEVQLGIPHPAGLKKKKSVTVLDVGDA
YFSVPLDEDFRKYTAFTIPSTNNETPGIRYQYNVLPQGWKGSPAIFQSSMTKILEPFRKQNPEIVIYQYMDDLYVGSDLEIGQHRIKIEE
LREHLLKWGFTTPDKKHQKEPPFLWMGYELHPDKWTVQPIKLPEKESWTVNDIQKLVGKLNWASQIYPGIKVKQLCKLLRGAKALTE
VVPLTEEAELELAENREILKEPVHGVYYDPSKDLIAEIQKQGQDQWTYQIYQEPFKNLKTGKYAKMRGAHTNDVKQLTEAVQKIAQECI
VIWGKTPKFRLPIQKETWETWWTEYWQATWIPEWEFVNTPPLVKLWYQLEKEPIIGAETFYVDGAANRETKLGKAGYVTDRGRQKV
VSLTDTTNQKTELQAINLALQDSGLEVNIVTDSQYALGIIQAQPDKSESELVSQIIEQLIKKEKVYLAWVPAHKGIGGNEQVDKLVSNGIR
KILFLDGIDKAQEEHEKYHNNWRAMASDFNLPPVVAKEIVASCDKCQLKGEAMHGQVDCSPGIWQLDCTHLEGKVILVAVHVASGYIE
AEVIPAETGQETAYFLLKLAGRWPVKVVHTDNGSNFTSAAVKAACWWAGIKQEFGIPYNPQSQGVVESMNKELKKIIGQVRDQAEHL
KTAVQMAVFIHNFKRKGGIGGYSAGERIIDIIATDIQTKELQKQITKIQNFRVYYRDSRDPIWKGPAKLLWKGEGAVVIQDNSDIKVVPR
RKAKIIRDYGKQMAGDDCVASRQDED
>Pol_CON_D_2010s
FFRENLAFPQGKARELPSEQTRANSPTSRELRVWGGDNTLSETGAERQGAVSFSFPQITLWQRPLVTVKIGGQLKEALLDTGADDTV
LEEINLPGKWKPKMIGGIGGFIKVRQYDQILVEICGHKAIGTVLVGPTPVNIIGRNLLTQIGCTLNFPISPIETVPVKLKPGMDGPKVKQW
PLTEEKIKALTEICTEMEKEGKISRIGPENPYNTPIFAIKKKDSTKWRKLVDFRELNKRTQDFWEVQLGIPHPAGLKKKKSVTVLDVGDA
YFSVPLYEDFRKYTAFTIPSINNETPGIRYQYNVLPQGWKGSPAIFQSSMTKILEPFRKQNPEIVIYQYMDDLYVGSDLEIGQHRIKIEEL
REHLLKWGFTTPDKKHQKEPPFLWMGYELHPDKWTVQPIKLPEKESWTVNDIQKLVGKLNWASQIYPGIKVRQLCKCIRGAKALTEV
VPLTEEAELELAENREILKEPVHGVYYDPSKDLIAEIQKQGQDQWTYQIYQEQYKNLKTGKYAKMRGTHTNDVKQLTEAVQKIAQECI
VIWGKTPKFRLPIQKETWETWWTEYWQATWIPEWEFVNTPPLVKLWYQLEKEPIVGAETFYVDGAANRETKLGKAGYVTDRGRQKV
VSLTDTTNQKTELQAINLALQDSGLEVNIVTDSQYALGIIQAQPDKSESELVNQIIEQLIKKEKVYLSWVPAHKGIGGNEQVDKLVSNGIR
KILFLDGIDKAQEEHEKYHNNWRAMASDFNLPPVVAKEIVASCDKCQLKGEALHGQVDCSPGIWQLDCTHLEGKVILVAVHVASGYIE
AEVIPAETGQETAYFLLKLAGRWPVKVVHTDNGSNFTSAAVKAACWWAGIKQEFGIPYNPQSQGVVESMNKELKKIIGQVRDQAEHL
KTAVQMAVFIHNFKRKGGIGGYSAGERIIDIIATDIQTKELQKQITKIQNFRVYYRDSRDPIWKGPAKLLWKGEGAVVIQDNSEIKVVPRR
KAKIIRDYGKQMAGDDCVASRQDED
>Pol_CON_F1_2000s
FFRENLAFPQGEARKFPSEQARANSPTSRELRVRRGDNPLSEAGAEGRGTVPSLSFPQITLWQRPVVTIKVGGQLKEALLDTGADDT
VLEDINLPGKWKPKMIGGIGGFIKVKQYDNILIDICGHKAIGTVLVGPTPVNIIGRNMLTQIGCTLNFPISPIETVPVKLKPGMDGPKVKQ
WPLTEEKIKALTEICTEMEKEGKISKIGPENPYNTPVFAIKKKDSTKWRKLVDFRELNKRTQDFWEVQLGIPHPAGLKKKKSVTVLDVG
DAYFSVPLDKEFRKYTAFTIPSINNETPGIRYQYNVLPQGWKGSPAIFQCSMTKILEPFRTKNPDIVIYQYMDDLYVGSDLEIGQHRAKI
EELREHLLKWGFTTPDKKHQKEPPFLWMGYELHPDKWTVQPIQLPDKDSWTVNDIQKLVGKLNWASQIYPGIKVKQLCKLLRGAKAL
TDIVPLTAEAELELAENREILKEPVHGVYYDPSKDLIAEIQKQGQGQWTYQIYQEPFKNLKTGKYAKMRSAHTNDVKQLTEAVQKIALE
SIVIWGKTPKFKLPILKETWDTWWTEYWQATWIPEWEFVNTPPLVKLWYQLETEPIAGAETFYVDGASNRETKKGKAGYVTDRGRQK

AVSLTETTNQKAELQAIQLALQDSGSEVNIVTDSQYALGIIQAQPDKSESELVNQIIEQLIQKEKVYLSWVPAHKGIGGNEQVDKLVSAG
IRKILFLDGIDKAQEEHEKYHNNWRAMASDFNLPPVVAKEIVASCDKCQLKGEAMHGQVDCSPGIWQLDCTHLEGKIILVAVHVASGYI
EAEVIPAETGQETAYFILKLAGRWPVKTIHTDNGTNFTSAAVKAACWWAGIQQEFGIPYNPQSQGVVESMNKELKKIIGQVRDQAEHL
KTAVQMAVFIHNFKRKGGIGGYSAGERIIDIIATDIQTKELQKQIIKIQNFRVYYRDSRDPVWKGPAKLLWKGEGAVVIQDNSEIKVVPR
RKAKIIRDYGKQMAGDDCVAGRQDED
>Pol_CON_F1_2010s
FFRENLAFQQGEARKFPSEQARANSPASRELRVQGGDNPLSEAGAERRGTVPSLSFPQITLWQRPLVTVKVGGQLKEALLDTGADD
TVLEDINLPGKWKPKMIGGIGGFIKVKQYDNILIDICGHKATGTVLVGPTPVNIIGRNMLTQIGCTLNFPISPIETVPVKLKPGMDGPKVK
QWPLTEEKIKALTEICTEMEKEGKISKIGPENPYNTPVFAIKKKDSTKWRKLVDFRELNKRTQDFWEVQLGIPHPAGLKKKKSVTVLDV
GDAYFSVPLDKEFRKYTAFTIPSLNNETPGIRYQYNVLPQGWKGSPAIFQCSMTKILEPFRAKNPELVIYQYMDDLYVGSDLEIGQHRA
KIEELRDHLLKWGFTTPDKKHQKEPPFLWMGYELHPDKWTVQPIQLPEKDSWTVNDIQKLVGKLNWASQIYPGIKVKQLCKLLRGAK
ALTDIVPLTAEAELELAENREILKEPVHGVYYDPSKDLIAEIQKQGQGQWTYQIYQEPFKNLKTGKYAKMRSAHTNDVKQLTEAVQKIA
LESIVIWGKTPKFRLPILKETWDTWWTEYWQATWIPEWEFVNTPPLVKLWYQLETEPIAGAETFYVDGASNRETKKGKAGYVTDRGR
QKAISLTETTNQKAELQAIQLALQDSGSEVNIVTDSQYALGIIQAQPDKSESELVNQIIEQLIKKEKVYLSWVPAHKGIGGNEQVDKLVS
AGIRKILFLDGIDKAQEDHEKYHNNWRAMASDFNLPPVVAKEIVASCDKCQLKGEAMHGQVDCSPGIWQLDCTHLEGKIILVAVHVAS
GYIEAEVIPAETGQETAYFILKLAGRWPVKTIHTDNGSNFTSAAVKAACWWAGIQQEFGIPYNPQSQGVVESMNKELKKIIGQIRDQAE
HLKTAVQMAVFIHNFKRKGGIGGYSAGERIIDIIATDIQTKELQKQIIKIQNFRVYYRDSRDPVWKGPAKLLWKGEGAVVIQDNSEIKVVP
RRKAKIIRDYGKQMAGDDCVAGRQDED
>Pol_CON_G_2000s
FFRENLAFQQGEAREFSSEQARANSPTSRELRVRRGDSPLPEAGAEGEGATSLSLPQITLWQRPLVTVRIGGQLIEALLDTGADDTVL
EQINLPGKWKPKMIGGIGGFIKVKQYDQILIEIEGKKAIGTVLVGPTPINIIGRNMLTQIGCTLNFPISPIETVPVKLKPGMDGPKVKQWPL
TEEKIKALTEICTEMEKEGKISKIGPENPYNTPIFAIKKKDSTKWRKLVDFRELNKRTQDFWEVQLGIPHPAGLKKKKSVTVLDVGDAYF
SVPLDESFRKYTAFTIPSINNETPGIRYQYNVLPQGWKGSPAIFQSSMTKILEPFRTKNPEMVIYQYMDDLYVGSDLEIGQHRAKIEELR
EHLLKWGFTTPDKKHQKEPPFLWMGYELHPDKWTVQPIQLPDKESWTVNDIQKLVGKLNWASQIYPGIKVKQLCKLLRGAKALTDIV
PLTAEAELELAENREILKEPVHGVYYDPSKELIAEVQKQGLDQWTYQIYQEPYKNLKTGKYAKRGSAHTNDVKQLTEVVQKIATESIVI
WGKIPKFKLPIRKETWEVWWTEYWQATWIPDWEFVNTPPLVKLWYQLETEPIPGAETYYVDGAANRETKLGKAGYVTDKGKQKIITL
TETTNQKTELQAIQLALQDSGSEVNIVTDSQYALGIIQAQPDRSESELVNQIIEQLIKKEKVYLSWVPAHKGIGGNEQVDKLVSSGIRKV
LFLDGIDKAQEEHERYHSNWRAMASDFNLPPIVAKEIVASCDKCQLKGEAMHGQVDCSPGIWQLDCTHLEGKIILVAVHVASGYIEAE
VIPAETGQETAYFILKLAGRWPVKVIHTDNGSNFTSAAVKAACWWANITQEFGIPYNPQSQGVVESMNKELKKIIGQVRDQAEHLKTA
VQMAVFIHNFKRKGGIGGYSAGERIIDIIASDIQTKELQKQITKIQNFRVYYRDSRDPIWKGPAKLLWKGEGAVVIQDNNEIKVVPRRKA
KIIRDYGKQMAGDDCVAGRQDED
>Pol_CON_G_2010s
FFRENLAFQQGEAREFSSEQARANSPTRRELRVRRGDSPLPEAGAEGKGATSFSLPQITLWQRPLVTVRIGGQLIEALLDTGADDTV
LEQIDLPGKWKPKMIGGIGGFIKVKQYDQIPIEIEGKKAIGTVLVGPTPINIIGRNMLTQIGCTLNFPISPIETVPVKLKPGMDGPKVKQWP
LTEEKIKALTEICTEMEKEGKISKIGPENPYNTPIFAIKKKDSTKWRKLVDFRELNKRTQDFWEVQLGIPHPAGLKKKKSVTVLDVGDAY
FSVPLDESFRKYTAFTIPSINNETPGIRYQYNVLPQGWKGSPAIFQSSMTKILEPFRTKNPEIVIYQYMDDLYVGSDLEIGQHRAKIEELR
EHLLKWGFTTPDKKHQKEPPFLWMGYELHPDKWTVQPIQLPDKESWTVNDIQKLVGKLNWASQIYPGIKVKQLCKLLRGAKALTDIV
PLTEEAELELAENREILKEPVHGVYYDPSKDLIAEVQKQGLDQWTYQIYQEPYKNLKTGKYAKRGSAHTNDVKQLTEAVQKIATESIVI
WGKVPKFKLPIRKETWEVWWTEYWQATWIPDWEFVNTPPLVKLWYQLETEPIPGAETYYVDGAANRETKLGKAGYVTDKGKQKIITI
QETTNQKAELHAIQLALQDSGSEVNIVTDSQYALGIIQAQPDRSESELVNQIIEQLIKKEKVYLTWVPAHKGIGGNEQVDKLVSSGIRKV
LFLDGIDKAQEEHERYHSNWRAMASDFNLPPIVAKEIVASCDKCQLKGEAMHGQVDCSPGIWQLDCTHLEGKIILVAVHVASGYIEAE
VIPAETGQETAYFILKLAGRWPVKVIHTDNGSNFTSNAVKAACWWANITQEFGIPYNPQSQGVVESMNKELKKIIGQVRDQAEHLKTA
VQMAVFIHNFKRKGGIGGYSAGERIIDIIASDIQTKELQKQITKIQNFRVYYRDSRDPIWKGPAKLLWKGEGAVVIQDNNEIKVVPRRKA
KIIRDYGKQMAGDDCVAGRQDED
>Pol_CON_01_AE_1990s
FFRENLAFQQGKAGEFSSEQTRANSPTSRKLGDGGRDNLLTEAGAERQGTSSSFSFPQITLWQRPLVTVKIGGQLKEALLDTGADDT
VLEDINLPGKWKPKMIGGIGGFIKVRQYDQILIEICGKKAIGTVLVGPTPVNIIGRNMLTQIGCTLNFPISPIDTVPVTLKPGMDGPKVKQ
WPLTEEKIKALTEICKEMEEEGKISKIGPENPYNTPVFAIKKKDSTKWRKLVDFRELNKRTQDFWEVQLGIPHPAGLKKKKSVTVLDVG
DAYFSVPLDESFRKYTAFTIPSINNETPGIRYQYNVLPQGWKGSPAIFQSSMTKILEPFRIKNPEIVIYQYMDDLYVGSDLEIGQHRTKIE
ELRAHLLSWGFTTPDKKHQKEPPFLWMGYELHPDRWTVQPIELPEKDSWTVNDIQKLVGKLNWASQIYAGIKVKQLCKLLRGAKALT
DIVPLTEEAELELAENREILKTPVHGVYYDPSKDLVAEVQKQGQDQWTYQIYQEPFKNLKTGKYARKRSAHTNDVRQLTEVVQKIATE
SIVIWGKTPKFRLPIQRETWETWWMEYWQATWIPEWEFVNTPPLVKLWYQLEKDPIVGAETFYVDGAASRETKLGKAGYVTDRGRQ
KVVSLTETTNQKTELHAIHLALQDSGSEVNIVTDSQYALGIIQAQPDRSESEVVNQIIEELIKKEKVYLSWVPAHKGIGGNEQVDKLVSS
GIRKVLFLDGIDKAQEEHERYHSNWRTMASDFNLPPIVAKEIVANCDKCQLKGEAMHGQVDCSPGIWQLDCTHLEGKVILVAVHVAS
GYIEAEVIPAETGQETAYFLLKLAGRWPVKVIHTDNGSNFTSAAVKAACWWANVRQEFGIPYNPQSQGVVESMNKELKKIIGQVREQ
AEHLKTAVQMAVFIHNFKRKGGIGGYSAGERIIDIIATDIQTKELQKQITKIQNFRVYYRDSRDPIWKGPAKLLWKGEGAVVIQDNSDIKV
VPRRKAKIIRDYGKQMAGDDCVAGRQDED
>Pol_CON_01_AE_2000s
FFRENLAFQQGKAREFSSEQTRANSPTSRKLGDGGRDNLLTEAGAERQGTSSSFSFPQITLWQRPLVTVKIGGQLKEALLDTGADDT
VLEDINLPGKWKPKMIGGIGGFIKVRQYDQILIEICGKKAIGTVLVGPTPVNIIGRNMLTQIGCTLNFPISPIDTVPVTLKPGMDGPKVKQ
WPLTEEKIKALTEICKEMEEEGKISKIGPENPYNTPVFAIKKKDSTKWRKLVDFRELNKRTQDFWEVQLGIPHPAGLKKKKSVTVLDVG
DAYFSVPLDESFRKYTAFTIPSINNETPGIRYQYNVLPQGWKGSPAIFQSSMTKILEPFRIKNPEIVIYQYMDDLYVGSDLEIGQHRTKIE
ELRAHLLSWGFTTPDKKHQKEPPFLWMGYELHPDRWTVQPIELPEKDSWTVNDIQKLVGKLNWASQIYAGIKVKQLCKLLRGAKALT
DIVPLTEEAELELAENREILKTPVHGVYYDPSKDLVAEVQKQGQDQWTYQIYQEPFKNLKTGKYARKRSAHTNDVRQLTEVVQKIATE
SIVIWGKTPKFRLPIQKETWETWWMEYWQATWIPEWEFVNTPPLVKLWYQLEKDPIVGAETFYVDGAASRETKLGKAGYVTDRGRQ
KVVSLTETTNQKTELHAIHLALQDSGSEVNIVTDSQYALGIIQAQPDRSESEVVNQIIEELIKKEKVYLSWVPAHKGIGGNEQVDKLVSS
GIRKVLFLDGIDKAQEEHERYHSNWRTMASDFNLPPIVAKEIVANCDKCQLKGEAMHGQVDCSPGIWQLDCTHLEGKVILVAVHVAS
GYIEAEVIPAETGQETAYFLLKLAGRWPVKVIHTDNGSNFTSAAVKAACWWANVRQEFGIPYNPQSQGVVESMNKELKKIIGQVREQ
AEHLKTAVQMAVFIHNFKRKGGIGGYSAGERIIDIIATDIQTKELQKQITKIQNFRVYYRDSRDPIWKGPAKLLWKGEGAVVIQDNSDIKV
VPRRKAKIIRDYGKQMAGDDCVAGRQDED

>Pol_CON_01_AE_2010s
FFRENLAFQQGKAREFSSEQTRANSPTSRKLGDGGRDNLLTEAGAERQGTSSSFSFPQITLWQRPLVTVKIGGQLKEALLDTGADDT
VLEDINLPGKWKPKMIGGIGGFIKVRQYDQILIEICGKKAIGTVLVGPTPVNIIGRNMLTQIGCTLNFPISPIDTVPVKLKPGMDGPKVKQ
WPLTEEKIKALTEICKEMEEEGKISKIGPENPYNTPVFAIKKKDSTKWRKLVDFRELNKRTQDFWEVQLGIPHPAGLKKKKSVTVLDVG
DAYFSVPLDESFRKYTAFTIPSINNETPGIRYQYNVLPQGWKGSPAIFQCSMTKILEPFRIKNPEIVIYQYMDDLYVGSDLEIGQHRTKIE
ELRAHLLSWGFTTPDKKHQKEPPFLWMGYELHPDRWTVQPIELPEKDSWTVNDIQKLVGKLNWASQIYAGIKVKQLCKLLRGAKALT
DIVPLTEEAELELAENREILKTPVHGVYYDPSKDLVAEVQKQGQDQWTYQIYQEPFKNLKTGKYARKRSAHTNDVRQLTEVVQKIATE
SIVIWGKTPKFRLPIQKETWETWWMEYWQATWIPEWEFVNTPPLVKLWYQLEKDPIVGAETFYVDGAASRETKLGKAGYVTDRGRQ
KVVSLTETTNQKTELHAIHLALQDSGSEVNIVTDSQYALGIIQAQPDRSESEVVNQIIEELIKKEKVYLSWVPAHKGIGGNEQVDKLVSS
GIRKVLFLDGIDKAQEEHERYHSNWRTMASDFNLPPIVAKEIVASCDKCQLKGEAMHGQVDCSPGIWQLDCTHLEGKVILVAVHVAS
GYIEAEVIPAETGQETAYFLLKLAGRWPVKVIHTDNGSNFTSAAVKAACWWANVRQEFGIPYNPQSQGVVESMNKELKKIIGQVREQ
AEHLKTAVQMAVFIHNFKRKGGIGGYSAGERIIDIIATDIQTKELQKQITKIQNFRVYYRDSRDPIWKGPAKLLWKGEGAVVIQDNSDIKV
VPRRKAKIIRDYGKQMAGDDCVAGRQDED
>Pol_CON_02_AG_2000s
FFRENLAFQQGEARKFSSEQTRTNSPTSRELWDGGRDNLLSEAGTEGQGTISSFNFPQITLWQRPLVTVRIGGQLIEALLDTGADDT
VLEEINLPGKWKPKMIGGIGGFIKVRQYDQILIEICGKKAIGTVLVGPTPVNIIGRNMLTQIGCTLNFPISPIETVPVKLKPGMDGPKVKQ
WPLTEEKIKALTEICTEMEKEGKISKIGPENPYNTPVFAIKKKDSTKWRKLVDFRELNKRTQDFWEVQLGIPHPAGLKKKKSVTVLDVG
DAYFSVPLDKDFRKYTAFTIPSVNNETPGIRYQYNVLPQGWKGSPAIFQASMTKILEPFRTKNPEIVIYQYMDDLYVGSDLEIGQHRAKI
EELREHLLKWGFTTPDKKHQKEPPFLWMGYELHPDKWTVQPIQLPEKDSWTVNDIQKLVGKLNWASQIYAGIKVKQLCKLLRGAKAL
TDIVTLTEEAELELAENREILKEPVHGVYYDPTKDLVAEIQKQGQDQWTYQIYQEPFKNLKTGKYAKKRSAHTNDVKQLTEVVQKVAT
ESIVIWGKTPKFRLPIQKETWEAWWMEYWQATWIPEWEFVNTPPLVKLWYQLEKDPIVGAETFYVDGAANRETKLGKAGYVTDRGR
QKVVSLTETTNQKTELHAIHLALQDSGSEVNIVTDSQYALGIIQAQPDRSESELVNQIIEKLIEKDKVYLSWVPAHKGIGGNEQVDKLVS
SGIRKVLFLDGIDKAQEEHERYHSNWRAMASDFNLPPIVAKEIVASCDKCQLKGEAMHGQVDCSPGIWQLDCTHLEGKIILVAVHVAS
GYIEAEVIPAETGQETAYFILKLAGRWPVKVIHTDNGSNFTSAAVKAACWWANVTQEFGIPYNPQSQGVVESMNKELKKIIGQVRDQA
EHLKTAVQMAVFIHNFKRKGGIGGYSAGERIIDIIASDIQTKELQKQITKIQNFRVYYRDSRDPIWKGPAKLLWKGEGAVVIQDNSDIKVV
PRRKAKIIKDYGKQMAGDDCVAGRQDED
>Pol_CON_02_AG_2010s
FFRENLAFQQGEARKFSSEQTRTNSPTSRELWDGGRDNLLSEAGTEGQGTISSFNFPQITLWQRPLVTVRIGGQLIEALLDTGADDT
VLEEINLPGKWKPKMIGGIGGFIKVRQYDQILIEICGKKAIGTVLVGPTPVNIIGRNMLTQIGCTLNFPISPIETVPVKLKPGMDGPKVKQ
WPLTEEKIKALTEICTEMEKEGKISKIGPENPYNTPVFAIKKKDSTKWRKLVDFRELNKRTQDFWEVQLGIPHPAGLKKKKSVTVLDVG
DAYFSVPLDKDFRKYTAFTIPSVNNETPGIRYQYNVLPQGWKGSPAIFQASMTKILEPFRTKNPEIVIYQYMDDLYVGSDLEIGQHRAKI
EELREHLLRWGFTTPDKKHQKEPPFLWMGYELHPDKWTVQPIQLPEKDSWTVNDIQKLVGKLNWASQIYAGIKVKQLCKLLRGAKAL
TDIVTLTEEAELELAENREILKEPVHGVYYDPTKDLVAEIQKQGQDQWTYQIYQEPFKNLKTGKYAKKRSAHTNDVKQLTEVVQKVAT
ESIVIWGKTPKFRLPIQKETWEAWWMEYWQATWIPEWEFVNTPPLVKLWYQLEKDPIVGAETFYVDGAANRETKLGKAGYVTDRGR
QKVVSLTETTNQKTELHAIYLALQDSGSEVNIVTDSQYALGIIQAQPDRSESELVNQIIEKLIEKDKVYLSWVPAHKGIGGNEQVDKLVS
SGIRKVLFLDGIDKAQEEHERYHSNWRAMASDFNLPPIVAKEIVASCDKCQLKGEAMHGQVDCSPGIWQLDCTHLEGKIILVAVHVAS
GYIEAEVIPAETGQETAYFILKLAGRWPVKVIHTDNGSNFTSAAVKAACWWANVTQEFGIPYNPQSQGVVESMNKELKKIIGQVRDQA
EHLKTAVQMAVFIHNFKRKGGIGGYSAGERIIDIIASDIQTKELQKQITKIQNFRVYYRDSRDPIWKGPAKLLWKGEGAVVIQDNSDIKVV
PRRKAKIIRDYGKQMAGDDCVAGRQDED
>Pol_CON_07_BC_2000s
FFRENLALPQGKAREFSSEQTRANSPTRGELQVWGRDNNSISEAGADRQGTISLNFPQITLWQRPLVTIKIGGQLKEALLDTGADDTV
LEDMNLPGKWKPKMIGGIGGFIKVRQYEQIPIEICGHKAIGTVLVGPTPVNIIGRNLLTQLGCTLNFPISPIETVPVKLKPGMDGPKVKQ
WPLTEEKIKALTAICDEMEKEGKITKIGPENPYNTPIFAIKKKDSTKWRKLVDFRELNKRTQDFWEVQLGIPHPAGLKKKKSVTVLDVG
DAYFSVPLYEDFRKYTAFTIPSINNETPGIRYQYNVLPQGWKGSPAIFQSSMTKILEPFRKQNPDIVIYQYMDDLYVGSDLEIGQHRTKI
EELRQHLLRWGFTTPDKKHQKEPPFLWMGYELHPDKWTVQPIQLPEKDSWTVNDIQKLVGKLNWASQIYPGIKVRQLCKLLRGAKAL
TDIVPLTEEAELELAENREILKEPVHGVYYDPSKDLIAEIQKQGQDQWTYQIYQEPFKNLKTGKYAKMRTAHTNDVKQLTEAVQKIAME
SIVIWGKTPKFRLPIQKETWETWWTDYWQATWIPEWEFVNTPPLVKLWYQLEKDPIAGAETFYVDGAANRETKIGKAGYVTDRGRKK
VVSLTETTNQKTELQAICIALQDSGSEVNIVTDSQYALGIIQAQPDKSESELVNQIIEQLIKKERVYLSWVPAHKGIGGNEQVDKLVSSGI
RKVLFLDGIDKAQEEHERYHSNWRAMASDFNLPPIVAKEIVASCDQCQLKGEAMHGQVDCSPGIWQLDCTHLEGKIILVAVHVASGY
MEAEVIPAETGQETAYFILKLAGRWPVKVIHTDNGSNFTSAAVKAACWWAGIQQEFGIPYNPQSQGVVESMNKELKKIIGQVRDQAE
HLKTAVQMAVFIHNFKRKGGIGGYSAGERIIDIIATDIQTKELQKQITKIQNFRVYYRDSRDPSWKGPAKLLWKGEGAVVIQDNSDIKVV
PRRKAKIIKDYGKQMAGADCVAGRQDED
>Pol_CON_07_BC_2010s
FFRENLALPQGKAREFSSEQTRANSPTRGELQVWGRDNNSISKAGALNFPQITLWQRPLVTIKIGGQLKEALLDTGADDTVLEDMNLP
GKWKPKMIGGIGGFIKVRQYEQIPIEICGHKAIGTVLIGPTPVNIIGRNLLTQLGCTLNFPISPIETVPVKLKPGMDGPKVKQWPLTKEKIE
ALTAICDEMEKEGKITKIGPENPYNTPIFAIKKKDSTKWRKLVDFRELNKRTQDFWEVQLGIPHPAGLKKKKSVTVLDVGDAYFSVPLY
EDFRKYTAFTIPSINNETPGIRYQYNVLPQGWKGSPAIFQSSMTKILEPFRKQNPDIVIYQYMDDLYVGSDLEIGQHRTKIEELRQHLLR
WGFTTPDKKHQKEPPFLWMGYELHPDKWTVQPIQLPVQDSWTVNDIQKLVGKLNWASQIYPGIKVRQLCKLLRGAKALTDIVPLTEE
AELELAENREILKEPVHGVYYDPSKDLIAEIQKQGQDQWSYQIYQEPFKNLKTGKYAKMRTAHTNDVKQLTEAVQKIAMESIVIWGKTP
KFRLPIQKETWETWWTDYWQATWIPEWEFVNTPPLVKLWYQLEKDPIAGVETFYVDGAANRETKIGKAGYVTDRGRKKVVSLTETT
NQKTELHAICLALQDSGSEVNIVTDSQYALGIIQAQPDKSESELVNQIIEQLIKKERVYLSWVPAHKGIGGNEQVDKLVSSGIRKVLFLD
GIDKAQEEHEKYHSNWRAMASDFNLPPIVAKEIVASCDQCQLKGEAMHGQVDCSPGIWQLDCTHLEGKIILVAVHVASGYMEAEVIP
AETGQETAYFILKLAGRWPVKVIHTDNGSNFTSAAVKAACWWAGLQQEFGIPYNPQSQGVVESMNKELKKIIGQVRDQAEHLKTAVQ
MAVFIHNFKRKGGIGGYSAGERIIDIIATDIQTKELQKQITKIQNFRVYYRDSRDPSWKGPAKLLWKGEGAVVIQDNSDIKVVPRRKAKII
KDYGKQMAGADCVAGRQDED
